# Artesunate Dose Escalation for the Treatment of Uncomplicated Malaria in a Region of Reported Artemisinin Resistance: A Randomized Clinical Trial

**DOI:** 10.1371/journal.pone.0019283

**Published:** 2011-05-13

**Authors:** Delia Bethell, Youry Se, Chanthap Lon, Stuart Tyner, David Saunders, Sabaithip Sriwichai, Sea Darapiseth, Paktiya Teja-Isavadharm, Phisit Khemawoot, Kurt Schaecher, Wiriya Ruttvisutinunt, Jessica Lin, Worachet Kuntawungin, Panita Gosi, Ans Timmermans, Bryan Smith, Duong Socheat, Mark M. Fukuda

**Affiliations:** 1 Department of Immunology and Medicine, Armed Forces Research Institute of Medical Sciences (AFRIMS), Bangkok, Thailand; 2 Center for Parasitology, Entomology and Malaria Control, Pnom Penh, Cambodia; Kenya Medical Research Institute - Wellcome Trust Research Programme, Kenya

## Abstract

**Background:**

The emergence of artemisinin resistance has raised concerns that the most potent antimalarial drug may be under threat. The currently recommended daily dose of artesunate (AS) is 4 mg/kg, and is administered for 3 days together with a partner antimalarial drug. This study investigated the impact of different AS doses on clinical and parasitological responses in malaria patients from an area of known artemisinin resistance in western Cambodia.

**Methods:**

Adult patients with uncomplicated *P. falciparum* malaria were randomized into one of three 7-day AS monotherapy regimens: 2, 4 or 6 mg/kg/day (total dose 14, 28 and 42 mg/kg). Clinical, parasitological, pharmacokinetic and *in vitro* drug sensitivity data was collected over a 7-day inpatient period and during weekly follow-up to 42 days.

**Results:**

143 patients were enrolled (n = 75, 40 and 28 to receive AS 2, 4 and 6 mg/kg/day respectively). Cure rates were high in all treatment groups at 42 days despite almost half the patients remaining parasitemic on Day 3. There was no impact of increasing AS dose on median parasite clearance times, median parasite clearance rates or on the proportion of patients remaining parasitemic on Day 3. However at the lowest dose used (2 mg/kg/d) patients with parasitemia >10,000/µL had longer median (IQR) parasite clearance times than those with parasitemia <10,000/µL (63 (48–75) vs. 84 (66–96) hours, p<0.0001). 19% of patients in the high-dose arm developed neutropenia (absolute neutrophil count <1.0×10^9^/L) by Day 14 and resulted in the arm being halted early.

**Conclusion:**

There is no pharmacodynamic benefit of increasing the daily dose of AS (4mg/kg) currently recommended for short-course combination treatment of uncomplicated malaria, even in regions with emerging artemisinin resistance, as long as the partner drug retains high efficacy.

**Trial Registration:**

ClinicalTrials.gov NCT00722150.

## Introduction

The emergence of artemisinin resistant malaria along the Thai-Cambodian border has provoked global alarm that the most valuable and effective antimalarial drug is in danger of being lost, triggering a campaign to identify and eradicate resistant parasite strains [1], [2] [3]. Despite these concerns the artemisinin resistance phenotype has been poorly characterized. Decreased parasite sensitivity to the artemisinin drugs in standardized *in vitro* tests has not been reported to date from clinical trials and a molecular marker for artemisinin resistance remains elusive. The key features of the phenotype appear to be prolonged parasite clearance times and slower than expected parasite clearance rates in patients with adequate plasma drug concentrations [4]. Recently a genetic basis for this clinical phenotype has been proposed [5]. While cases of slow parasitological responses can occur sporadically and at low frequency in any malaria setting, more than 10% of patients in a given population remaining parasitemic after 3 days of artemisinin combination therapy (ACT) treatment has been suggested as a figure to warrant further detailed investigation of parasitological responses to treatment using AS monotherapy [6], [4].

Experimental seven-day AS monotherapy regimens, although impractical and inadvisable for routine, unsupervised use, are valuable research tools and have yielded important scientific information about potentially resistant parasites without the confounding influence of the partner drug and without compromising antimalarial treatment efficacy; a daily dose of 4 mg/kg in a 7-day AS monotherapy regimen was found to have 28-day efficacy of 93% in a previous study conducted in 2007 in Tasanh, western Cambodia [7].

This study formed part of the ARC3 (Artemisinin Resistance: Confirmation, Characterization and planning for Containment) Project, in which 4 sites (Tasanh and Pailin in western Cambodia, Wang Pa in northern Thailand and Bandarban in Bangladesh) were selected to evaluate 7-day artesunate (AS) monotherapy regimens using harmonized protocols and common endpoints [4]. Data from Pailin and Wang Pa have already been published and demonstrated for the first time that parasitological outcomes were significantly different between malaria patients in Pailin and those in Wang Pa, with slower parasite clearance times in western Cambodia [8]. Although PCR-adjusted failure rates were higher in Pailin than Wang Pa the difference was not statistically significant. The principal objective of this study in Tasanh, an area of known emerging artemisinin resistance, was to determine the impact of AS dose on clinical and parasitological responses in patients with uncomplicated falciparum malaria by employing 3 different AS monotherapy regimens and to test the hypothesis that higher doses of AS than are currently recommended (>4 mg/kg) might be effective in eliminating artemisinin resistant strains. An additional objective was to identify and characterize resistant malaria parasite populations and describe the clinical phenotype of drug resistant malaria. Since safety data for this class of drug is limited a further area of focus was to characterize the safety and tolerability of higher cumulative doses of AS.

## Methods

The protocol for this trial and supporting CONSORT checklist are available as supporting information; see Checklist S1 and Protocol S1.

### Design

This was a randomized, open-label comparison of 3 regimens of AS monotherapy given as a single oral dose of 2, 4 or 6 mg/kg/day (AS2, AS4 and AS6) for 7 days (total doses 14, 28 and 42 mg/kg) in otherwise healthy adult patients with acute falciparum malaria. AS2 was selected as the lower limit of what was considered an effective dose to probe for clinical resistance. The ratio of enrollment into the 3 groups was 2∶1∶2 into AS2, AS4 and AS6 respectively; AS4 was intended to serve as a control and act as a bridge to an earlier study performed at the same site in 2006/2007 [7], [9].

### Study site

**Figure 1:**
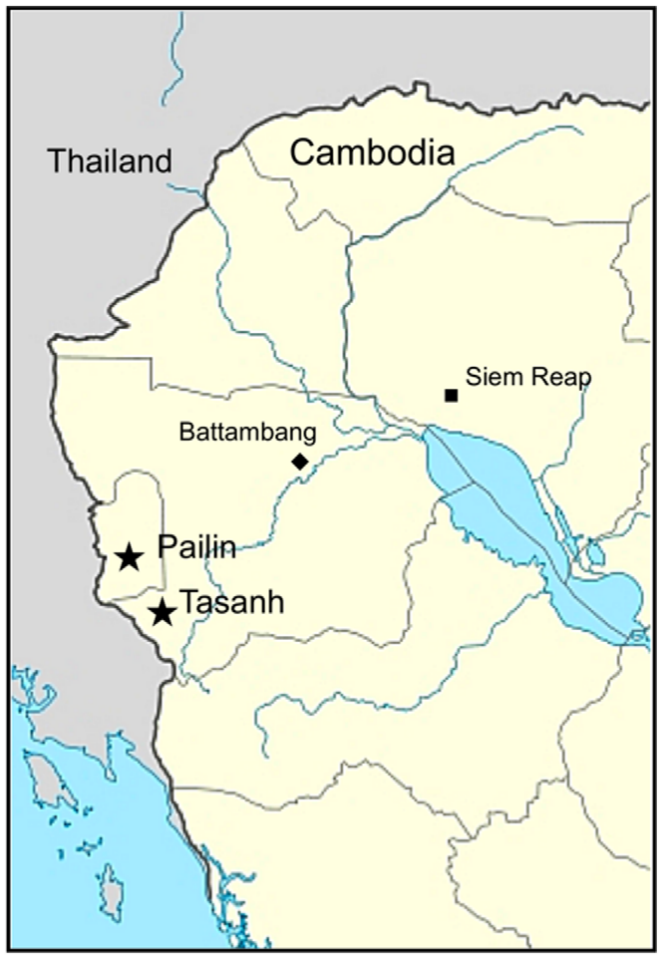
Study location.

Tasanh Health Center is located in Battambang Province in western Cambodia, close to the Thailand border and due south of Pailin (Figure 1); this is an area where reports of increasing rates of ACT failure have emerged over recent years [1]; [8].

### Entry criteria

Consecutive patients were recruited into the study if they fulfilled the following criteria: (1) acute symptomatic *P. falciparum* monoinfection as determined by microscopy with a parasite density of 1000 to 200,000 asexual parasites/µL; (2) fever/history of fever within 48 hours; (3) age 18–65 years; (4) gave written informed consent to participate; (5) otherwise healthy outpatients. Exclusion criteria included the following: (1) pregnancy or unwillingness to use effective contraception if female and of child-bearing age; (2) history of intolerance or hypersensitivity to AS or other artemisinin derivatives; (3) history of any malaria drug therapy within 30 days; (4) history of other significant illness; (5) signs or symptoms indicating a requirement for parenteral antimalarial therapy; (6) signs or symptoms of severe malaria [10].

### Randomization and dosing

Randomization was done by an independent statistician using computer-allocated blocks of 10 and was not stratified; individual treatment allocations were contained inside consecutively numbered sealed envelopes, which were opened sequentially by a study investigator or clinical research coordinator (CRC) after the decision to enroll a subject had been made by the study team. AS doses (using 50 mg tablets, Guilin Pharmaceutical Co. Ltd., Guilin, China; quality-controlled and supplied by the World Health Organization) were calculated individually based on the patient's weight at enrollment and the dose rounded up to the nearest ¼ tablet (12.5 mg). Doses were administered with water and separated from other concomitant medications wherever possible. All treatment was directly observed. The full dose was repeated if vomiting occurred within 30 minutes, and half-dose given if vomiting occurred within 30–60 minutes. Before the study commenced 10 randomly selected tablets of study drug were analyzed in-house for content and weight uniformity according to standard guidelines [11] [12]: the mean AS content was 45.9 mg (range 41.3–50.2 mg), and dihydroartemisinin (DHA) impurity was 1.31% (range 1.03–1.62%).

### Study procedures

Subjects remained as inpatients for the first 7 days then returned for follow-up on days 14, 21, 28, 35 and 42. Before initiating AS therapy blood was drawn to test for *in vitro* drug sensitivity, molecular markers of drug resistance and to distinguish later recrudescence from re-infection by parasite genotyping. Malaria smears were prepared up to 8 times on Day 0, and then 4 times a day until 2 consecutive smears were negative for asexual parasites, then daily until discharge from the ward and then weekly on days 14, 21, 28, 35, and 42, and again if symptoms consistent with malaria appeared during follow-up. Plasma samples for AS and DHA concentrations were collected on Day 0 (pre-dose, 15, 30, 60 minutes and 2, 4, 6 and 8 h), and Day 6 (pre-dose, 2, 4 and 6 h) doses of AS. Additional blood for parasite genotyping and culture was drawn at the time of treatment failure.

### Endpoints

Outcomes at 28 and 42 days, including early treatment failure (ETF), late treatment failure (LTF) and adequate clinical and parasitological response (ACPR) were classified according to standard definitions [13]. The parasite clearance time (PCT_100_) was defined as the time from the start of treatment until the first time the blood smear became negative for asexual parasites and remained negative at 2 consecutive measurements. PCT_90_ and PCT_50_ were times for the parasitemia to reduce to 90 and 50% of baseline value. Parasite reduction ratios (PRR) were calculated as 100 minus percentage reduction from baseline level at 24, 48 and 72 hours. The slope of the log_10_ transformed parasite clearance curves was used as a measure of parasite clearance rate and calculated using TableCurve 2D (Systat Software, San Jose, California). Fever clearance time (FCT) was defined as the time from baseline until the start of the period in which the tympanic temperature remained below 38°C for at least 24 hours.

### Safety

Because the highest dose arm (AS6) involved a total oral AS dose of 42 mg/kg, which is higher than other previously published studies, a number of safety features were built into the study design (Table 1). These included development of specific individual and cohort halting rules, the establishment of an independent chartered Safety Monitoring Committee (SMC), daily assessment of treatment emergent adverse events during the first 7 days, a pause for safety review of AS6 after the first 5 patients had completed treatment, and measurement of complete blood count (CBC) and alanine aminotransferase (ALT) at baseline, before the 4^th^ and 7^th^ AS doses and on Day 14.

**Table 1:** Safety Outcomes and Assessments.

Safety outcome monitored	Assessment	Individual Halting Rule	Cohort Halting Rules
Standard adverse event and serious adverse event monitoring	Reported from time of informed consent until final follow-up visit at D42	-	• >4 individual halts/arm for the same event;• >1 SAE/arm judged probably or definitely related to study drug• By discretion of study PI or SMC• Enrollment pause after first five subjects in AS6 with resumption pending SMC review
Treatment Emergent Adverse Event monitoring daily during artesunate therapy.	Daily physician clinical assessment grading severity by CTCAE criteria	-	
Myelosuppression	CBC with differential at D0, 3, 6 and 14	Neutrophils <1.0×10^9^ cells/L; Hemoglobin <7.0 mg/dL or decreased >3.6 mg/dL from baseline	
Neurologic effects	Standardized neurological examination daily for 7 days and at D14	Obtundation; new or worsening ataxia; >1 seizure	
Gastrointestinal or genitourinary	Patient report or direct observation of stools/urine	Visibly bloody stools or urine, not due to other etiology	
Hepatotoxicity	Daily physical examination for 7 days; Plasma ALT at D0, 3, 6 and 14	-	
Clinical efficacy	Symptom and parasitemia assessments daily for 7 days; efficacy at D 3, 7, 28 and 42.	Alternate (non artemisinin) regimen administered for late treatment failures	>30% clinical failures at D28 in any treatment arm

Key. D = day after start of treatment; CBC = complete blood count; SMC = Safety Monitoring Committee; ALT = alanine aminotransferase; SAE = serious adverse event; CTCAE = Common Terminology Criteria for Adverse Events[25].

### Microscopy

Giemsa-stained thick and thin blood smears were examined by two microscopists blinded to each other's results and to the treatment status of the study subject. Parasite densities were calculated based on a count of parasites per 200 WBCs (thick film) or per 5000 RBCs (thin film). At least 200 oil immersion fields were examined on the thick film before a blood smear was considered negative. The final count was determined by taking the geometric mean of the two microscopists counts. In case of a difference in results (positive/negative; species diagnosis) between the two microscopists, the blood smear was re-examined by a third microscopist independent of the earlier findings and the third reading accepted as the final result.

### Pharmacokinetics

Whole blood was collected into chilled sodium heparin tubes, centrifuged immediately, plasma separated and frozen at approximately −20°C or below. Samples were transferred to Bangkok for analysis by LC-MS [14]. C_max_ and T_max_ were estimated by inspection of data. Pharmacokinetic (PK) parameters area under the plasma concentration-time curve (AUC) and t_1/2_ were calculated for AS and it's major metabolite dihydroartemisinin (DHA) by non-compartmental analysis using PK Solutions Software (Summit Research Services).

### Bioassay

Plasma samples from blood collected into sodium heparin were frozen at −20°C and transported to AFRIMS for analysis. Determination of antimalarial activity in DHA equivalents against laboratory strains of *P. falciparum* (W2) indicating prior use of antimalarial drugs was performed using a previously described *ex vivo* bioassay method [15]; the cut-off value selected was 5 ng/ml (18 nM).

### Clinical labs

CBC samples were collected into EDTA tubes and analyzed using a Beckman Coulter® AcT5diff analyzer. Plasma ALT samples were measured using a Reflotron® Plus analyzer (Roche Diagnostics).

### *In vitro*

Fresh samples, without prior freezing or pre-culturing, were assayed in the histidine-rich protein 2 (HRP2) drug sensitivity *ex vivo* assay for susceptibility to DHA, AS, mefloquine (MQ), quinine (QN), chloroquine (CQ), and lumefantrine (LUM) [16]. Drug-coated plates were stored at 4°C and used within 8 weeks of coating. In order to avoid an innoculum effect, patient specimens were diluted to a parasitemia between 0.2–0.5% before plating to enable analysis of IC_50_ without the confounding effect of baseline parasitemia. Parasite culture and drug sensitivity assays were performed as previously described [17]. The CQ-resistant W2 *P. falciparum* clone was used as a reference and for quality control of drug-coated plates.

### Parasite genotyping

To distinguish recrudescence from new infection polymorphisms in genes encoding the *P. falciparum* merozoite surface proteins MSP-1 (National Center for Biotechnology Information (NCBI) gene ID 813575) and MSP-2 (NCBI gene ID 812660), and the glutamate-rich surface protein (GLURP) (NCBI gene ID 810501) were compared in individual samples from baseline and time of failure according to standard methodology [18] [19]. The polymorphic repetitive regions selected were block 2 of MSP-1 (allelic variants from the MAD20, K1 and RO33 families), block 3 of MSP-2 (allelic variants from the FC27 and the 3D7/IC families) and RII block of GLURP.

### Statistical analysis

Sample size estimation assumed a cure rate of 80% in AS2 and 95% in AS6 and was calculated as 60 patients in each of the two arms with >80% chance of detecting a significant difference in cure rate by uncorrected Chi-squared test at a two-sided 0.1 significance level. Proportions of patients, both adjusted and non-adjusted for new infections and for *P. vivax* parasitemia during follow-up, were compared between treatment groups using the Chi-squared and Fishers exact tests. Values of normally distributed data were expressed as means (95%CI) and non-normally distributed data as geometric means (95% CI) or medians (IQR), as appropriate. Means were compared using ANOVA; otherwise the non-parametric Kruskall-Wallis test was used. Comparison of failure rates between groups and the proportion of patients remaining parasitemic over time, were assessed by modified intention-to-treat (ITT) analysis using Kaplan-Meier methods and differences between groups compared using the Logrank test (WHO 2009). Data were analyzed using Stata 10.0 (College Station, Texas) and reported in accordance with CONSORT methodology [20].

## Results

**Figure 2:**
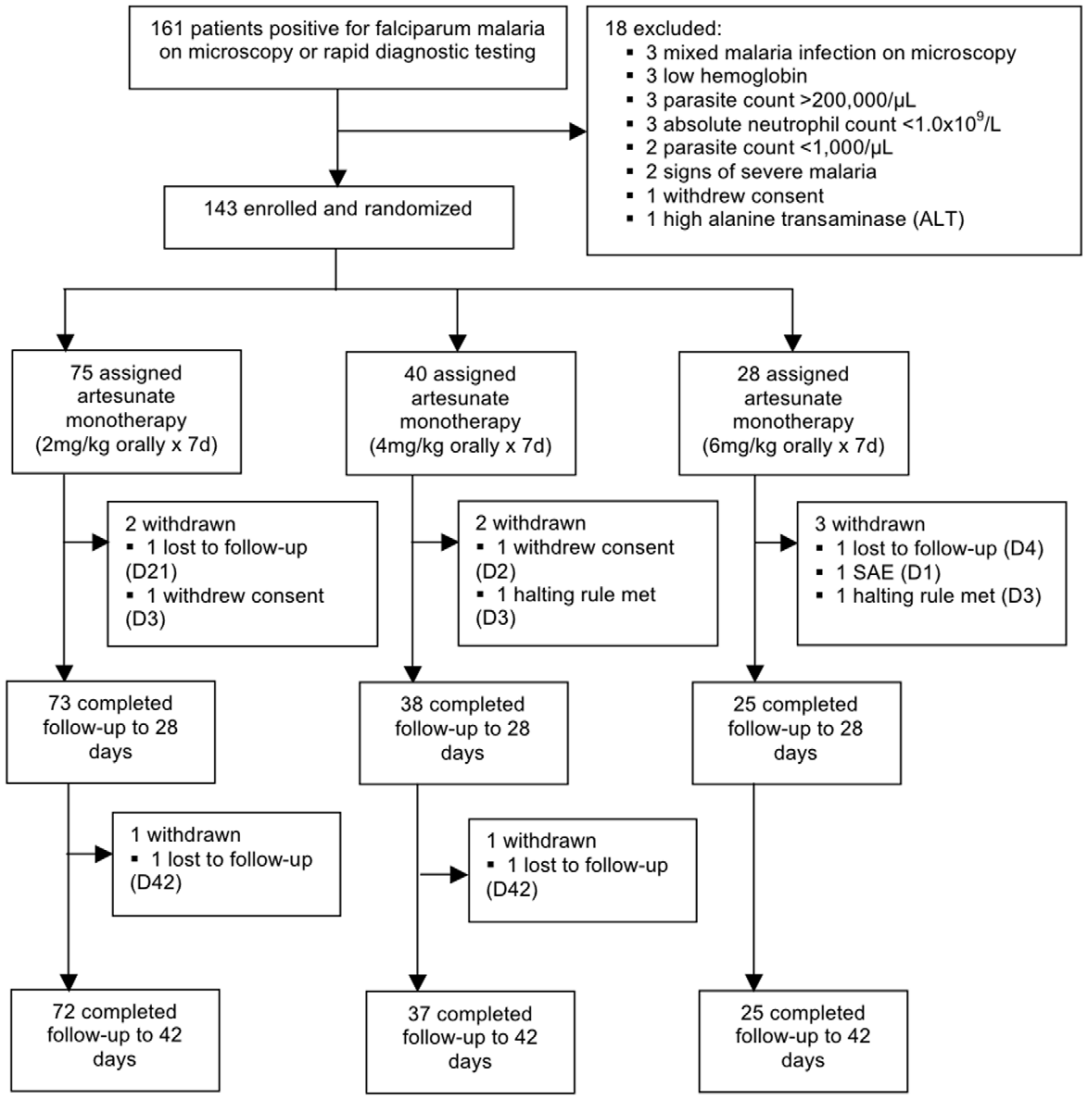
Enrollment, randomization and workflow.

From August 2008 until July 2009 161 patients were screened and 143 enrolled into the study (Figure 2). Seventy-five, 40 and 28 subjects were randomized to AS2, 4 and 6 respectively. Seven subjects were subsequently withdrawn; one (in AS6) deteriorated clinically on Day 1 requiring withdrawal from the study and transfer to another center for parenteral treatment; an additional 6 (2 in each arm) were either unwilling to continue study participation or lost to follow-up. Baseline characteristics were similar in the 3 treatment groups (Table 2). 136 patients completed 7 days of AS monotherapy and were followed to Day 28; 133 completed follow-up to Day 42 (Figure 2, Table 3). Median (range) weight-adjusted doses were 2 (1.9–2.3) mg/kg, 4 (3.9–4.1) mg/kg and 6 (5.9–6.1) mg/kg in the 3 groups respectively. Bioassay results from baseline indicated that only 8/136 (6%) samples had non-specific pre-existing antimalarial activity above the equivalent of 5 ng/mL DHA.

**Table 2:** Baseline Characteristics in 143 Patients; intention to treat analysis.

Parameter*	AS2(n = 75)	AS4(n = 40)	AS6(n = 28)	Significance∧
Male sex, n (%)	62 (83)	30 (75)	20 (71)	0.16/0.38
Age, y, median (IQR)	25 (20 to 35)	22 (20 to 32)	28 (18 to 38)	0.99/0.47
Weight, kg, mean (95% CI)	53 (51 to 54)	51 (48 to 53)	52 (49 to 54)	0.51/0.42
History of previous malaria episode, n (%)	29 (39)	11 (28)	7 (25)	0.24/0.13
Duration of symptoms, median (IQR)	3 (2 to 4)	3 (2 to 4)	3 (3 to 5)	0.10/0.23
Temperature, °C, mean (SD)	38.0 (37.8 to 38.3)	38.4 (38.0 to 38.7)	38.2 (37.9 to 38.6)	0.36/0.31
Parasitemia, geomean (95%CI)	13,077(9,872 to 17,324)	18,266(12,145 to 27,471)	10,923(6,682 to 17,855)	0.39/0.18
Parasite density group, /µL, n (%)≥1000 and ≤10,000>10,000 and ≤100,000>100,000	32 (43)40 (53)3 (4)	15 (38)22 (55)3 (7)	15 (54)11 (39)2 (7)	0.37/0.56
White-cell count, ×10^−3^/mm^3^, median (IQR)	5.8 (4.6 to 7.5)	6.0 (4.9 to 7.3)	5.4 (4.3 to 6.8)	0.20/0.38
Absolute neutrophil count, ×10^−3^/mm^3^, median (IQR)	3.8 (2.7 to 5.1)	4.2 (3.5 to 5.1)	3.8 (3.2 to 5.0)	0.79/0.54
Hemoglobin, g/dl, median (IQR)	12.7 (12.2 to 13.7)	13.3 (12.6 to 14.2)	12.8 (11.1 to 13.7)	0.44/0.21
Hematocrit, median (IQR)	39 (36 to 42)	40 (38 to 43)	37 (33 to 40)	**0.012/0.003**
Platelet count, ×10^−3^/mm^3^, median (IQR)	102,500(74,250 to 140,000)	107,500(58,000 to 151,250)	86,000(66,750 to 148,750)	0.51/0.83
Alanine aminotransferase, U/liter, median (IQR)	21.3 (14.0 to 37.6)	19.5 (13.7 to 30.5)	21.0 (12.0 to 32.6)	0.80/0.76
Bioassay positive, n (%)	4/72 (6)	1/38 (3)	3/26 (12)	0.27/0.30
Presence of Pf gametocytes, n (%)	10 (13)	4 (10)	6 (21)	0.24/0.40
IC_50_ of fresh cultured parasites, nM,geometric mean (95%CI)- AS- DHA- Mefloquine- Quinine- Chloroquine- Lumefantrine	4.3 (3.5–5.3)6.4 (5.0–8.3)40.8 (30.6–54.4)115.7 (89.0–150.3)236.7 (190.2–294.4)6.7 (5.2–8.7)	5.6 (4.4–7.0)7.9 (5.8–10.5)40.1 (26.6–60.5)106.0 (68.4–164.1)257.2 (188.1–351.6)6.1 (4.4–8.4)	7.3 (5.7–9.0)10.1 (8.1–12.5)41.6 (27.6–62.5)160.6 (123.0–209.6)351.1 (230.0–535.8)5.8 (3.8–8.8)	**0.045/**0.22**0.016/**0.110.94/0.960.19/0.41**0.013/0.049**0.51/0.79

Key: Values expressed as median (IQR) unless otherwise specified;

∧X/Y  =  comparison between AS2 and AS6/comparison between AS2, AS4 and AS6; y  =  year; Pf  =  *Plasmodium falciparum*;

**Table 3:** Clinical and parasitological responses in 143 patients with acute *P.falciparum* malaria receiving artesunate monotherapy: intention to treat analysis.

Variable, median (IQR)	AS2(n = 75)	AS4(n = 40)	AS6(n = 28)	Significance between groups¥
Completed 7 doses AS, n (%)	73 (97)	38 (95)	25 (89)	0.12/0.22
Met a halting rule, n (%)- Neutrophil count <1.0×10^9^/L- Hemoglobin drop >3.6 g/dL	000	2 (5)11	5 (18)50	**0.001/0.001**
Parasite clearance time, h- Overall- If parasitemia <10,000/mm^3^- If parasitemia ≥10,000 &<100,000/mm^3^- If parasitemia ≥100,000/mm^3^	74 (54 to 90)63 (48–75)81 (66–90)108 (108–120) ∧	78 (66 to 96)66 (42–78)85 (66–102)84 (72–102) ∧ ∧	78 (66 to 96)72 (66–85)81 (72–102)81 (54–108)	0.28/0.38**0.025**/0.090.52/0.460.37/0.19
Time to 50% clearance of parasites, h	7.8 (4.9 to 9.9)	9.3 (7.2 to 12.9)	8.2 (6.3 to 11.9)	0.21/0.11
Time to 90% clearance of parasites, h	19.7 (14.8 to 27.0)	23.5 (17.5 to 30.5)	20.6 (17.5 to 28.8)	0.30/0.26
Parasite-reduction ratio- At 24 h- At 48 h	7.05 (3.21 to 12.79)0.93 (0.19 to 2.41)	9.49 (4.06 to 15.85)0.93 (0.25 to 2.73)	7.65 (3.50 to 17.55)1.54 (0.33 to 3.77)	0.41/0.600.13/0.34
Slope of curve for log_10_-normalized parasite clearance	0.040(0.036 to 0.054)	0.043(0.036 to 0.055)	0.037(0.032 to 0.044)	0.11/0.13
Remained parasitemic at 72 h, n (%)	37/75 (49)	18/39 (46)	13/27 (48)	0.55/0.97
Fever clearance, h	14 (5–29)	18 (9–29)	20 (11–28)	0.20/0.26
Recurrent Pf parasitemia* during follow-up, n (%)- Baseline to day 28- Day 29 to day 42- Total	213 (4)	112 (5)	202 (7)	0.40/0.71
Pv parasitemia during follow-up, n (%)- Baseline to day 28- from day 29 to 42- overall	6612 (17)	257 (19)	617 (28)	0.22/0.59
Duration of gametocyte carriage, days∼	24 (6–27)	10 (5–17)	17 (14–27)	0.90/0.55

*not PCR-corrected; Kruskall-Wallis test used for comparison of medians;

¥ X/Y  =  comparison between AS2 and AS6/comparison between AS2, AS4 and AS6;

∼ gametocyte carriage documented in 10, 4 and 6 patients in AS2, 4 and 6 respectively;

∧ p<0.0001 for comparison of parasite clearance times for parasitemia groups within AS2;

∧ ∧ p = 0.49 for comparison of parasite clearance times for parasitemia groups within AS4; Pf  =  *Plasmodium falciparum*.

Eight months after the study started a Cohort Halting Rule was triggered when a fifth subject in AS6 developed neutropenia; safety analysis demonstrated significantly lower geometric mean absolute neutrophil counts (ANC) on Days 6 and 14 in patients in AS6 and led to permanent suspension of the arm; this finding has been reported in detail elsewhere [21]. Therefore fewer patients were recruited into AS6 than AS2. There were no clinically apparent adverse events associated with the neutropenia. Patients in AS6 were significantly more likely to meet a halting rule compared to the other 2 treatment arms (odds ratio 5.9 (95% CI 2.3–15.6, p = 0.0003).

### Clinical responses

**Figure 3:**
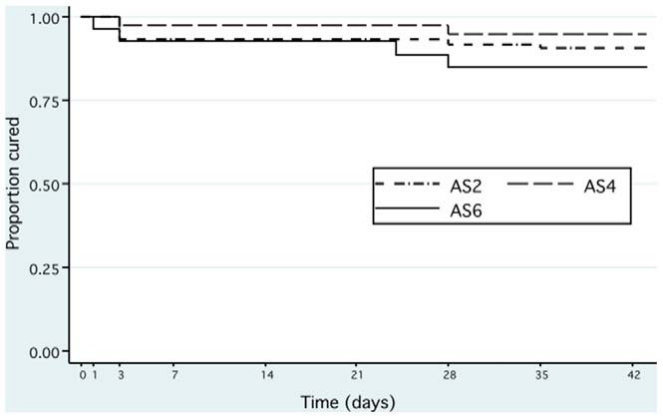
Modified intention-to-treat analysis: outcome measured to 42 days.

By Day 42 133 patients had met a per protocol efficacy endpoint: 8 were classified as ETF, 5 as PCR-adjusted LTF, and 120 as ACPR. Per protocol analysis showed no differences in outcome between treatment groups at Days 28 or 42; all AS regimens achieved cure in a high percentage of patients (Table 4). Modified ITT analysis confirmed no difference in cumulative failure rates between groups (Figure 3). Fever clearance times did not differ between treatment groups with median (IQR) clearance times of 14 (5–29), 18 (9–29) and 20 (11–28) hours in AS2, 4 and 6 respectively. Twenty-two patients required treatment with chloroquine for *P. vivax* infection during the course of the study (10, 5 and 7 in AS2, 4 and 6 respectively); removal of these cases from outcome analyses did not affect the results (Table 5).

**Table 4:** Outcome: per protocol analyses in patients at 28 (n = 136) and 42 (n = 133) days; *P. vivax* cases occurring during follow-up not removed.

Artesunate regimen	Outcome parameter*n (% of total)			Total number
	ETF	LTF*(PCR-adjusted)	ACPR	
**AS2**28 days42 days	5 (7)5 (7)	1 (1)2 (3)	66 (92)64 (90)	7271
**AS4**28 days42 days	1 (3)1 (3)	1 (3)1 (3)	36 (94)34 (94)	3836
**AS6**28 days42 days	2 (8)2 (8)	2 (8)2 (8)	22 (84)22 (84)	2626

Key. Fishers exact test shows no differences between treatment groups at 28 or 42 days (p = 0.41 and p = 0.65 respectively); ETF  =  early treatment failure; LTF  =  PCR-corrected late treatment failure; ACPR  =  adequate clinical and parasitological response [13];

*LTFs were further classified as follows: AS2: 1 × LCF at 28d and 1 × LCF at 35d; AS4: 1 × LPF at 28d; AS6: 1 × LCF at 24d and 1 × LPF at 28d.

**Table 5:** Outcome: per protocol analysis in 109 patients at 42 days; *P. vivax* relapse cases censored from analysis.

Artesunate regimen	Outcome parameter*n (% of total)			Total number
	ETF	LTF(PCR-adjusted)	ACPR	
**AS2**42 days	5 (8)	2 (3)	53 (88)	60
**AS4**42 days	1 (3)	1 (3)	27 (90)	30
**AS6**42 days	2 (11)	2 (11)	15 (79)	19

Key. Fishers exact test shows no differences between treatment groups at 42 days (p = 0.57); ETF  =  early treatment failure; LTF  =  PCR-corrected late treatment failure; ACPR  =  adequate clinical and parasitological response [13].

#### Early treatment failures

Of the ETF cases, one (in AS6) was classified as ETF after a clinical deterioration on Day 1, which required transfer to another center for continued care. The remaining 7 cases had fever plus parasitemia on Day 3, though all were much improved clinically from presentation (Table 6). Six were allowed to continue their randomized AS regimen and all cleared parasites and fever within the following 1–2 days. One of these 6 patients subsequently had reappearance of mixed *P. falciparum/P. vivax* parasites on Day 35, but PCR genotyping indicated a new P. falciparum infection; the other 5 remained parasite-free to Day 42. The 7^th^ patient received rescue treatment with quinine-tetracycline on Day 4 and recovered uneventfully.

**Table 6:** Individual data for patients with reappearance of *Plasmodium falciparum* parasites during follow-up and median (IQR) values for cured subjects in the three dosing arms.

Subject	Agey	Sex	Drug	Baseline Parasitemia (/µL)	C_max_ Day 0		AUC_0-8_ Day 0		IC_50_ (nM)		PCT_50_ h	PCT_90_ h	PCT_100_ h	FCT h	Day of failure	PCR	Outcome
					AS	DHA	AS	DHA	AS	DHA							
1	20	M	AS2	7,779	187	868	154	1869	-	-	6	25	72	0	28	NI	LPF
2	18	M	AS2	72,305	57	396	84	954	4.8	5.1	11	39	96	27	28	R	LCF
3	27	M	AS2	8,689	108	1440	172	3067	20.6	27.2	9	25	84	19	35	R	LCF
4	22	F	AS4	22,863	1206	2055	579	2457	6.9	5.5	8	23	96	18	28	R	LPF
5	39	M	AS4	39,393	895	8461	1424	20837	7.5	14.2	4	10	79	9	42	NI	LPF
6	18	M	AS6	55,513	588	1642	472	5701	4.9	8.9	12	35	108	30	28	R	LPF
7	18	M	AS6	4,776	80	378	308	1893	10.7	16.0	39	43	108	27	24	R	LCF
8*	18	F	AS2	38,567	112	1231	107	2098	4.9	4.5	4	22	96	6	3/35	NI	ETF + LCF
9**	25	M	AS2	3,585	30	311	70	697	7.3	17.6	8	27	84	0	58	R**	VLTFPV D21
Cured AS2n = 64	26 (21–35)	-	AS2	12,748 (5,428–25,294)	80 (42–147)	536 (310–902)	81 (61–143)	1,285 (911–2,028)	4.6 (3.3–6.5)	6.8 (4.2–11.9)	8(5–10)	18(15–27)	72 (54–87)	12(5–27)	-	-	ACPR
Cured AS4n = 34	22 (20–35)	-	AS4	20,999 (6,227–49,406)	195 (117–280)	1,468 (875–2,185)	249 (191–310)	3,453 (2,807–4,833)	6.0 (3.5–8.6)	8.2 (5.2–13.9)	10(7–13)	24(17–31)	76 (60–96)	17(8–28)	-	-	ACPR
Cured AS6n = 22	30 (19–38)	-	AS6	5,571 (3,661–17,010)	327 (213–617)	2,177 (1,680–3,074)	401 (285–637)	6,086 (4,591–7,139)	7.0 (4.7–8.3)	11.1 (6.2–14.7)	7(5–10)	22 (18–28)	75 (66–90)	17 (9–27)	-	-	ACPR

Key. C_max_  =  maximum plasma concentration (nM); AUC  =  area under the plasma concentration – time curve (h.nM); FCT  =  fever clearance time; PCT  =  parasite clearance time; Cured  =  subjects not meeting a parasitological endpoint (ETF, LTF) and completing 42 days follow-up; NI  =  new infection by PCR correction; R  =  recrudescent infection by PCR correction; PV =  vivax parasitemia on blood smear requiring chloroquine treatment; VLTF  =  very late (>42d) treatment failure;

* LTF not included in per protocol analysis as subject had already met an endpoint on Day 3 (ETF);

** not included in per protocol analysis because recrudescence occurred after Day 42.

#### Late treatment failures

*P. falciparum* parasites reappeared during 42 days of follow-up in 8 cases, of whom 5 were confirmed as recrudescence by PCR genotyping (Table 6). A 9^th^ patient returned with *P. falciparum* parasitemia on Day 58, 16 days outside the follow-up period; PCR genotyping confirmed recrudescence; notably this patient had received CQ 25 mg/kg for *P. vivax* infection detected on Day 21, which may have delayed the *P. falciparum* recrudescence. PCR genotyping of MSP-1, MSP-2 and GLURP in baseline samples indicated that 7 of these 9 patients had polyclonal infections.

### Parasitological responses

**Figure 4:**
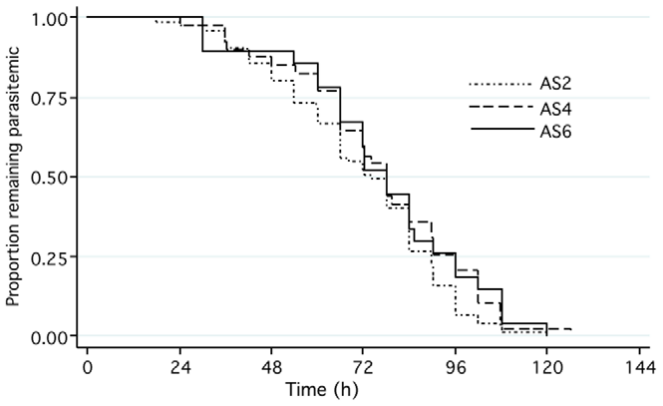
Modified intention-to-treat analysis: proportion of patients remaining parasitemic by treatment arm.

ITT analysis showed no differences between groups in median PCT_100_, PCT_90_, or PCT_50_, or in parasite reduction ratios at 24, 48 or 72 hours (Table 3, Figure 4). However, within AS2 patients with parasitemia >10,000/µL had significantly longer median (IQR) PCT_100_ than those with baseline parasitemia <10,000/µL (63 (48–75) vs. 84 (66–96) hours, p<0.0001); there was a similar but smaller effect seen in AS4 (p = 0.049), but not in AS6 (p = 0.65). This dose-effect was not seen for PCT_90_ or PCT_50_. Comparison of the slope of the log_10_ transformed parasite clearance curves showed no significant difference between treatment groups, and multiple regression analysis using slope as the dependent variable showed no significant association with parasitemia, history of previous malaria, IC_50_ DHA or treatment allocation. However comparison of the parasite clearance slopes between patients with a subsequent per protocol failure and those cured at 42 days showed a small but significant difference (0.037 (0.032–0.039 vs 0.041 (0.034–0.054, p = 0.032). Seventy-two hours after AS treatment commenced almost 50% of patients still had asexual parasites on a peripheral blood smear (49%, 46% and 48% in AS2, 4 and 6 respectively).

Comparison of parasitological responses between recrudescent subjects and those successfully cured at 42 days showed significant differences in PCT_100_ (Table 7). However the median PCT_100_ for cured subjects was still 72 hours, which is similar to the published value from Pailin (Table 8). Only 24 (17%) patients had rapid PCT_100_ (≤48 h) (Table 9). Compared to the 29 patients with PCT_100_ ≥96 h the rapid clearance group had significantly lower median baseline parasitemia and fewer per protocol failures (p<0.001 and p = 0.004 respectively); moreover the median slope of the log_10_ transformed parasite clearance curves was steeper in these rapid clearers than in patients with PCT_100_ ≥96 hours (0.03 vs 0.08, p = 0.0001). Plasma concentrations of AS and DHA could not explain the difference in clearance between these two groups. Compared to patients with PCT_100_ >48 h patients with PCT_100_ ≤48 h were more likely to have a history of previous malaria (58% vs 28%, p = 0.006). For patients remaining parasitemic at Day 3 or with PCT_100_ ≥96 h the odds ratios (95% CI) of per protocol failure were 5.9 (1.2–29.3) and 4.6 (1.3–16.1) respectively. For patients remaining parasitemic at Day 3 or with PCT_100_ ≥96 h the odds ratios (95% CI) of PCR-adjusted recrudescence within 42 days were 4.4 (0.5–41.8) and 16.9 (1.6–176.5) respectively.

**Table 7:** Parasitological, clinical and *in vitro* responses in early treatment failures, recrudescent and cured patients; data expressed as median (IQR) unless otherwise stated.

Parasitological and clinical responses	Cured at 42 days(n = 120)[Table-fn nt115]	Early treatment failure(n = 8)	*Comparison of cured and ETF* ∧	Recrudescent within 42d(n = 5)	*Comparison of cured and LTF* ∧
Baseline parasitemia, /µL, geometric mean (95%CI)	11,406(4,760–30,736)	38,981(21,398–48,294)	*0.07*	22,863(8,689–55,513)	*0.34*
C_max_ DHA Day 0, nM	917(498–1681)	1068(683–2304)	*0.45*	1440(396–1642)	*0.82*
AUC_0-8_ DHA Day 0, h.nM	2283(1212–4291)	2306(1537–6701)	*0.60*	2457(1893–3067)	*0.85*
PCT_50_, h	8 (5–11)	11 (5–18)	*0.32*	11 (9–12)	***0.08***
PCT_90_, h	20 (15–28)	35 (15–43)	*0.12*	35 (25–39)	***0.02***
PCT_100_, h	73 (60–90)	90 (84–96)	***0.02***	96 (96–108)	***0.006***
PRR_24_	7.1 (3.4–13.2)	21.4 (9.1–36.6)	***0.01***	18.9 (12.8–21.7)	***0.03***
PRR_48_	0.94 (0.23–2.61)	2.41 (1.22–5.57)	***0.02***	3.34 (1.87–3.77)	***0.02***
Slope of curve for log_10_-normalized parasite clearance	0.041(0.034–0.054)	0.037(0.030–0.038)	*0.09*	0.037(0.033–0.039)	*0.18*
FCT, h	15 (7–28)	81 (13–84)	***0.005***	27 (19–27)	*0.15*
IC_50_ DHA, nMBaselineRecrudescent	8.0 (5.0–13.5)-	10.6 (4.5–14.0)-	*0.70*	8.9 (5.5–16.0)7.0 (5.1–9.2)	*0.49*
IC_50_ AS, nMBaselineRecrudescent	5.4 (3.4–7.7)-	5.8 (4.1–7.2)-	*0.61*	6.9 (4.9–10.7)4.6 (4.5–7.5)	*0.13*

Cured refers to patients not meeting a parasitological endpoint and completing 42 days follow-up; recrudescent refers to PCR-corrected late treatment failures occurring during the 42 day follow-up; ∧Comparison of non-parametric data using Kruskall-Wallis test.

**Table 8:** Comparison of clinical and parasitological outcomes with other published data (Dondorp, 2009).

	TasanhAll		TasanhParasites ≥10^4^/µL §		PailinParasites ≥10^4^/µL		Wang PaParasites ≥10^4^/µL	
Outcomes	AS2(n = 75)	AS4(n = 40)	AS2(n = 43)	AS4(n = 25)	AS2(n = 20)	AS4*(n = 20)	AS2(n = 20)	AS4*(n = 20)
PCT_100_ (h), overall, median (IQR)if P_0_ < 10^4^/µLif P_0_ ≥10^4^and <10^5^/µLif P_0_ ≥10^5^/µL	74 (54–90)63 (48–75)81 (66–90)108 (108–120)	78 (66–96)66 (42–78)85 (66–102)84 (72–102)	84 (66–96)-81 (66–90)108 (108–120)	84 (72–102)-85 (66–102)84 (72–102)	85.9 (54, 96)-72 (48–96)90 (84–96)	72 (60, 96)-66 (60–84)96 (90–108)	54.1 (42, 72)-54 (42–72)54 (54–72)	48 (30.1, 54)-48 (30–54)60∧ (−)
PCT_50_, h,median (range)	8(0.2–31)	9(1.9–22)	8(1–18)	10(2–16)	11(1–25)	9(1–22)	4(1–24)	3(1–16)
PCT_90_, h,median (range)	20(2.2–58.6)	24(4–50)	20(10–43)	24(10–50)	23(8–39)	21(11–44)	12(3–40)	11(3–27)
PRR_24_,median (range)	7(0–170)	9(0–41)	8(0–31)	10(0–24)	18.7(0.3–97.2)	13.5(0.9–67.6)	1.1(0.0–54.1)	0.03(0.0–15.0)
PRR_48_,median (range)	0.9(0–19.1)	0.9(0–7.2)	0.9(0–7.5)	0.7(0–7.0)	0.5(0.0–11.4)	0.6(0.0–10.0)	0.0(0.0–3.8)	0.0(0.0–0.5)
Parasitemic at 72h, n (%)	37(49)	18(46)	29(67)	14(56)	22/40(55)¥		3/40(8)¥	
ETF, n (%)	5 (7)	1 (3)	4 (11)	1 (4)	3/40 (8)¥		0 (0)	0 (0)
Recrudescence,n (%)	2 (3)	1 (2)	1 (2)	1 (4)	6 (30)	1 (5)	2 (10)	1 (5)
Reinfection,n (%)	1	1 (2)	1 (2)	1 (4)	1 (5)	0 (0)	8 (40)	4 (20)
PV or mixed,n (%)	11/1 (15/1)	7/0 (19/0)	6/1 (14/2)	1/0 (4/0)	5/1 (30/5)	7/0 (35/0)	1/3 (20/15)	0/2 (10/10)
FCT, d,median (IQR)	0.6(0.2, 1.2)	0.8(0.4, 1.2)	0.7(0.3, 1.2)	1.0(0.4, 1.3)	50% had FCT >7d	3(2, -)	2(1, 2)	2(1, 2)
Gam. clearance, d, median (IQR)	24(6–27)	10(5–17)	17(4–27)	-	10(7–19)	18(6–23)	19(7–19)	13(1–19)

§Tasanh patients with baseline parasitemia >10,000 parasites µ/L;

*Pailin and Wang Pa AS4 groups received AS 4 mg/kg ×3 days followed by mefloquine 15 and 10 mg/kg on days 3 and 4;

∧ n = 1 therefore IQR not possible;

¥ Paper does not differentiate this value by treatment arm; P_0_  =  baseline parasitemia; Gam  =  gametocyte; Data are expressed in the units used in the Dondorp paper [8].

**Table 9:** Comparison of clinical, parasitological and laboratory parameters of patients with rapid or slow parasite clearance.

ParametersMedian (IQR)	Long PCT (≥96 h)n = 29	Rapid PCT (≤48 h)n = 24	Comparison between groups
Age, y	24 (20–31)	25 (19–34)	1.0
Weight, Kg	52 (50–55)	52 (46–57)	0.79
Male sex, n (%)	24 (83)	21 (88)	p = 0.47
History of previous malaria, n (%)1 episode2 episodes3 episodes>3 episodes	10 (34)8110	14 (58)*6521	p = 0.07
History of artemisinin use for a previous malaria episode, n (%)	1 (3)	5 (21)**	p = 0.06
History of mefloquine use for a previous malaria episode, n (%)	2 (7)	3 (13)	p = 0.41
Positive bioassay at baseline, n (%)	0/29 (0)	1/23 (4)	P = 0.44
Parasitemia, (/µL), geo.mean (95% CI)< 10,000/µL, n (%)≥10,000 and <100,000/µL, n (%)≥100,000/µL, n (%)	31,884 (21,209–47,930)4/62 (6)20/73 (27)5/8 (63) Δ	8,158 (4,717–14,110)15/62 (24)9/73 (12)0/8 (0) ΔΔ	**p = 0.0005**
Gametocytes at baseline, n (% positive)	3 (10)	1 (3)	p = 0.56
AS regimen, n (% of group)AS2AS4AS6	12 (16)10 (29)7 (25)	15 (20)6 (15)3 (11)	p = 0.33
PCT_100_, h	102 (96–108)	36 (30–42)	**p = 0.0001**
PCT_50_, h	10.7 (9.5–15.1)	5.9 (3.6–8.0)	**p = 0.0001**
PCT_90_, h	31.2 (24.4–37.2)	13.6 (10.1–16.9)	**p = 0.0001**
PRR_24_	14.3 (10.5–21.7)	1.2 (0.1–3.2)	**p = 0.0001**
Slope of curve for log_10_-normalized parasite clearance	0.035 (0.031–0.037)	0.084 (0.057–0.107)	**p = 0.0001**
IC_50_ DHA, geometric mean (95% CI)	9.0 (7.4–11.0)	6.5 (4.6–9.1)	p = 0.09
IC_50_ AS, geometric mean (95% CI)	6.2 (5.2–7.4)	4.0 (2.9–5.5)	**p = 0.04**
IC_50_ Mefloquine, geometric mean (95% CI)	37.4 (23.5–59.5)	44.2 (28.2–69.3)	p = 0.72
IC_50_ Chloroquine, geometric mean (95% CI)	324 (247–425)	290 (228–370)	p = 0.51
OutcomeETF, n (%)Recrudescence, n (%)Any per protocol failure, n (%)	246	000	p = 0.33p = 0.09**p = 0.02**
Vivax infection, n (%)	5/27 (17)	6/23 (26)	p = 0.26
FCT, h,	18.8 (10.5–28.7)	14.2 (0.4–25.6)	*p = 0.12*
C_max_ DHA, nM,	1286 (766–2085)	829 (400–1471)	*p = 0.19*
AUC_0-8_ * DHA, h.nM,	2457 (2007–4760)	1961 (1259–3081)	*p = 0.26*

Δ Patients with prolonged PCT more likely to have higher parasitemia than rest of study patients, p<0.0001;

ΔΔ p = 0.086 for rapid PCT patients compared to rest of study patients;

*p = 0.005 comparing rapid PCT group with rest of study patients; **p = 0.033 for rapid PCT patients compared to rest of study patients.

Significantly more patients remained parasitemic at 72 hours in the second compared to the first 6 months of the study (58 vs 39%, p = 0.016), despite an even distribution of treatment allocation in each period. Patients in the second half of the study were less likely to give a history of a diagnosis of malaria within the previous 12 months (55 vs 77% p = 0.005). They also had a lower occurrence of gametocytes on a baseline peripheral blood smear (7 vs 22%, p = 0.009) and a reduced *P. vivax* relapse rate during follow-up (14 vs 25%, p = 0.08).

### Pharmacokinetics

Pharmacokinetic (PK) profiles were obtained following the first AS dose in all 143 subjects and also around the final dose in the 136 subjects completing 7 days of monotherapy. As expected median C_max_ and AUC_0-8h_ for AS and DHA increased with AS dose but there was wide variation in individual plasma levels. PK values (C_max_ and AUC_0-8h_ for AS and DHA) for PCR-confirmed recrudescence cases were all above the 25^th^ percentile of the median of the respective parameters compared to the 64 subjects cured with AS 2 mg/kg/day. In aggregate the 8 patients classified as ETF had comparable drug levels to those cured (Table 7); however, one case had low AUC and another low C_max_ for both AS and DHA. Comparing PK parameters from this study to recently published data (Table 10), half-life and T_max_ were comparable but overall drug exposure based on C_max_ and AUC expressed in the same units was higher in Tasanh.

**Table 10:** Comparison of baseline and pharmacokinetic parameters with other published data (Dondorp, 2009).

	TasanhAll		TasanhParasites ≥10^4^/µL §		PailinParasites ≥10^4^/µL		Wang PaParasites ≥10^4^/µL	
Baseline characteristics	AS2(n = 75)	AS4(n = 40)	AS2(n = 43)	AS4(n = 25)	AS2(n = 20)	AS4*(n = 20)	AS2(n = 20)	AS4*(n = 20)
Age, y,mean (95% CI)	28.9(26.4, 31.3)	27.0(23.4, 30.5)	28.7(25.4, 32.0)	25.6(21.3, 29.9)	26.6(20.5, 32.7)	21.6(16.4, 26.9)	31.4(27.8, 34.9)	29.7(25.5, 33.9)
Weight, kg,median (IQR)	53.0(47.5, 56.3)	50.5(47.4, 54.9)	52.7(47.7, 55.7)	51.5(47.8, 55.1)	46.5(20.5, 60)	47.0(21, 62)	55(35, 66)	51(39, 59)
Male sex, n (%)	62 (83)	30 (75)	36 (84)	19 (76)	15 (75)	16 (80)	19 (95)	20 (100)
Hemoglobin, g/dL,mean (95% CI)	13.0(12.6, 13.3)	13.2(12.6, 13.8)	12.4(12.1, 14.0)	13.7(12.7, 14.8)	11.8(10.8, 12.8)	12.0(10.9, 13.1)	12.5(11.7, 13.3)	12.4(11.6, 13.3)
Platelets, ×10^3^/µL,median (IQR)	103(74–140)	108(58–151)	96(70–152)	84(48–127)	82(17–357)	113.5(40–383)	120.5(47–410)	104(29–357)
Parasitemia, /µL, geometric mean (95% CI)	13077(9872, 17324)	18266(12145, 27471)	31174(24934, 38977)	42258(31702, 56327)	64166(39155, 105153)	65299(40813, 104474)	37214,(26391, 52477)	22746(13888, 37252)
Gametocytes,n (% positive)	10 (13)	4 (10)	6 (14)	0 (0)	5 (25)	6 (30)	6 (30)	5 (25)
**Pharmacokinetics**								
C_max_ AS, nM,median (range)	219(29–2500)	578(198–3141)	196(49–2500)	556(197–3141)	248(28–897)	290(34–1620)	186(35–544)	219(50–1560)
T_max_ AS, h,median (range)	0.5(0.25–4.0)	0.5(0.25–2.0	0.5(0.25–4.0)	0.5(0.25–1.0)	0.5(0.25–2.0)	1.0(0.25–2.0)	0.38(0.25–0.98)	0.50.25–3.0)
AUC** AS, h.nM,median (range)	219(60–1140)	716(336–3708)	218(56–1141)	718(386–3707)	159(53–342)	310(122–738)	139(70–252)	264(93–553)
T_1/2_ AS, h,median (range)	0.46(0.12–2.7)	0.45(0.17–5.4)	0.46(0.12–2.1)	0.48(0.17–5.4)	0.29(0.13–1.42)	0.33(0.18–1.05)	0.37(0.14–3.00)	0.55(0.13–1.13)
C_max_ DHA, nM,median (range)	1908(507–5801)	5560(1697–29792)	1998(505–5804)	6193(1696–11764)	737(142–2780)	1460(771–4680)	937(162–1766)	1300(511–3500)
T_max_ DHA, h,median (range)	1.0(0.5–6.0)	1.0(0.5–4.0)	1.0(0.5–6.0)	1.0(0.5–4.0)	1.0(0.5–4.0)	1.5(0.42–3.0)	1.0(0.48–2.98)	1.01(0.48–3.0)
AUC** DHA, h.nMmedian (range)	4556(827–11975)	12944(5493–73370)	4529(826–11975)	13125(5491–73370)	1270(490–4030)	3780(1710–5200)	1430(604–2900)	3240(1480–6220)
T_1/2_ DHA, hmedian (range)	0.8(0.3–3.2)	0.9(0.4–3.5)	0.7(0.4–3.2)	0.9(0.4–3.5)	0.8(0.5–1.4)	0.7(0.5–1.5)	0.7(0.4–1.3)	0.8(0.5–1.4)

§ Tasanh patients with baseline parasitemia >10,000 parasites µ/L;

*Pailin and Wang Pa AS4 groups received AS 4 mg/kg ×3 days followed by mefloquine 15 and 10 mg/kg on days 3 and 4;

**AUC is 0–8 h for Tasanh and 0–24 hours for Pailin/Wang Pa; P_0_  =  baseline parasitemia; Gam  =  gametocyte; Data are expressed in the units used in the Dondorp paper [8].

### *In vitro* drug-sensitivity testing

**Figure 5:**
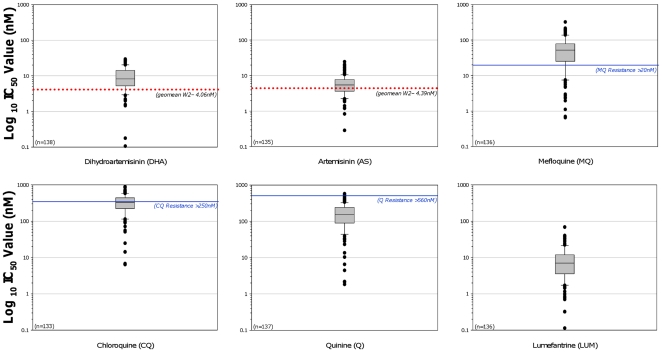
IC_50_ values at baseline using HRP2 methodology in fresh cultured parasite isolates from 131 patients.

IC_50_ data are shown in Figure 5. Sigmoidal dose response curves were obtained from DHA and AS coated HRP2 ELISA plates in 131 of 143 (92%) subjects; 67/75 in AS2; 39/40 in AS4, and 25/28 in AS6. Median (IQR) IC_50_ values for DHA were 8.19 (IQR 5.13–14.0) nM and for AS were 5.66 (3.57–7.81) nM, more than double the values measured at the same site using the same methodology 3 years previously ^1^. Individual IC_50_ values for DHA and AS did not correlate well with most parasitological parameters, and did not differentiate between cured and recrudescent patients (Table 7). In contrast, the median (IQR) DHA IC_50_ for patients remaining parasitemic at 72 hours was 9.60 (6.82–14.7) nM, significantly higher than the median IC_50_ of 6.26 (4.22–13.4) nM for subjects clearing parasites within 72 hours (p = 0.013). There was no significant difference observed between AS IC_50_ for patients who were and were not parasitemic at 72 hours (5.98 vs. 5.38 nM, p = 0.067).

## Discussion

This is the largest reported clinical trial to characterize clinical and parasitological responses to AS monotherapy in a region of artemisinin resistant malaria and evaluate a public health strategy to overcome these slow responses. Although impractical and inadvisable for routine, unsupervised use, AS monotherapy regimens are important research tools and allow detailed analysis of the dose response to AS without the confounding influence of a partner drug. Despite high treatment efficacy almost 50% of patients in this study population remained parasitemic 72 h after commencing AS, suggesting an alarming trend towards artemisinin resistance in the parasite population [4], [6]. Moreover PCTs were similar to those reported from Pailin, previously the best characterized area of emerging resistance, despite a 4-fold lower median parasitemia in Tasanh [8].

This study was designed to investigate whether resistant *P. falciparum* strains may require higher doses of AS to achieve the same clinical and pharmacodynamic effects as fully sensitive strains. However, because per protocol cure rates in Tasanh were high in all dosing groups, a finding in keeping with some other recent studies of AS monotherapy, we did not demonstrate improved 42-day efficacy using high-dose treatment. Even if the high-dose arm had recruited its planned allocation of subjects we would not have been able to demonstrate a dose-dependent effect between groups in terms of clinical outcome. This contrasts with the 30% recrudescence rate and prolonged fever clearance seen in Pailin; there, the high failure rate might in part be explained by the much higher median parasitemia at baseline (due to differing entry criteria), and lower apparent drug concentrations (Table 10) as well as a smaller sample size. Even the higher parasitemia patients in Tasanh did not have this clinical failure rate. Interestingly, the proportion of patients in Tasanh remaining parasitemic at 72 hours noticeably increased as the study progressed, which could indicate the emergence of more resistant clones in the parasite population [5], as well as reflect the changing epidemiology of malaria in western Cambodia as evidenced by fewer patients reporting a history of malaria diagnosed within the preceding 12 months. Median parasite clearance times in Tasanh were still considerably longer than those reported in Wang Pa in northern Thailand, despite lower median parasitemias in Tasanh, demonstrating that the phenomenon of slow parasite clearance is not simply dictated by the baseline parasitemia.

We did not demonstrate any improvement in pharmacodynamic responses when higher doses of AS were given. Within AS2 however we did observe that parasitemia >10,000/µL is associated with significantly prolonged median PCT_100_. This is likely to be explained by plasma drug concentrations being much closer to the MIC than when higher AS doses were administered. Interestingly this dose effect was not observed for PCT_90_ or PCT_50_; one explanation is that the more resistant clone(s) in a polyclonal infection may require more parasite lifecycles before complete killing occurs, and that PCT_50_ and PCT_90_ values reflect killing of the more sensitive clones. Only 17% patients in this study were rapid parasite clearers (PCT_100_ ≤48 h); these patients had a significantly lower median parasitemia and a higher reported history of previous malaria episodes indicating some degree of prior immunity; none failed treatment during 42d. The influence of both parasitemia and immunity on parasite clearance times has been well described previously [22]. The only significant dose-dependent adverse effect that was seen was the finding of myelotoxicity in AS6 which we have reported in detail elsewhere [21]. Clearly, dose escalation of the artemisinin component of an ACT cannot be considered a safe public health strategy until the effects of the drug on the neutrophil in malaria patients have been better defined, particularly if other factors known to be myelosuppressive, including partner antimalarial drugs, are present.

The lack of a clear difference in median IC_50_ for DHA and AS between failures and cures was surprising, but is in keeping with previous reports from this region [8]. One explanation is that, since some *P. falciparum* infections are known to be polyclonal, the wild-type, non-artemisinin resistant clone may have a selective advantage in the environment over the artemisinin resistant clone. Although the degree of polyclonality of malaria infections in Tasanh has not yet been demonstrated, PCR genotyping in the subset of patients from this study with reappearance of *P. falciparum* parasites indicated that 7 of 9 (78%) had polyclonal infections at baseline. Moreover analysis of both Thai and African isolates by Druihle et al revealed 33 and 34 clones respectively in primary specimens [23]; although many of these isolated clones were related genetically, they exhibited high degrees of diversity including their *in vitro* responses to antimalarial drugs. Our *in vitro* technique involves culture of fresh isolates in the field (“ex-vivo”) to mitigate losing clones, perhaps artemisinin resistant clones, which may not survive cryopreservation. Even so, this technique still does not allow easy differentiation of small clonal populations from within a polyclonal primary sample. The finding of lower median IC_50_ DHA and AS in recrudescent isolates at baseline might be explained by the very low parasitemias seen at recrudescence. HRP2 is produced by parasites and there is a strong relationship between parasite count and HRP2 level; in high parasitemias this is corrected for by dilution of the fresh sample prior to plating, but the converse is not possible with current methodologies. Additional work is needed to standardize *in vitro* methodologies as well as optimize strategies for isolating resistant clones from polyclonal infections.

A crucial observation was that 5 of 6 cases meeting ETF criteria of fever and parasitemia on Day 3 who continued AS monotherapy went on to clear parasites and remained cured to Day 42 without a requirement for rescue treatment; the 6^th^ patient had a mixed infection on Day 35 but the *P. falciparum* component was a new infection. This indicates that prolonged courses of AS were still effective in curing the malaria infection despite slow clinical and parasitological responses. Two of these ETF cases had sub-optimal plasma drug concentrations despite receiving weight-based dosing and directly observed therapy. The huge variability seen in individual pharmacokinetic profiles in malaria patients, even in the highly controlled environment of a clinical research trial, emphasizes the importance of controlling unregulated use of poor quality drugs, which likely plays a significant role in inducing resistance in low transmission settings [24].

In summary this study demonstrates that the parasitological response to antimalarial therapy is a complex interaction of factors including parasitemia, immune status, plasma drug concentrations and innate parasite resistance to the antimalarial drug. Increased doses of AS did not impact on clinical or pharmacodynamic outcomes in this setting of emerging artemisinin resistant malaria and were toxic at the highest cumulative dose evaluated. Since completion of this clinical trial, Tasanh has been at the center of a high profile containment program; only time will tell if these efforts have been successful or whether artemisinin resistant clones have already spread. Artemisinin resistance remains a significant threat to the malaria-endemic world and this study highlights that simple dose escalation is not a viable strategy to overcome it. Continued coordinated public health strategies are necessary to keep this threat at bay.

## Supporting Information

Checklist S1 CONSORT Checklist
(DOC)

Protocol S1 Trial Protocol
(DOC)
