# Supplementary material for: Artesunate Dose Escalation for the Treatment of Uncomplicated Malaria in a Region of Reported Artemisinin Resistance: A Randomized Clinical Trial
Source: PLoS One. 2011 May 13;6(5):e19283. doi: 10.1371/journal.pone.0019283 (PMC3094355; doi:10.1371/journal.pone.0019283)
Supplement: Protocol S1 — Trial Protocol (DOC) [file pone.0019283.s002.doc]

**ARTEMISININ RESISTANCE IN CAMBODIA II**

**WRAIR Protocol Number:**

*WRAIR #1396.*

*HSRRB Log Number A-14479*

**Principal Investigators:**

Dr. Youry Se

Dr. Duong Socheat

LTC Mark Fukuda

**Version Number:** *Version 1.6*

**23 May 2008**

SIGNATURE PAGE

The signatures below document the approval of this protocol and the attachments, and provide the necessary assurances that this study will be conducted according to all stipulations of the protocol, including all statements regarding confidentiality and according to local legal and regulatory requirements.

| Investigators: | | | |
| --- | --- | --- | --- |
| Signed: |  | Date: |  |
|  | *Name:*  Dr Youry Se |  |  |
|  | | | |
| Signed: |  | Date: |  |
|  | *Name:* Dr Duong Socheat |  |  |
|  | | | |
| Signed: |  | Date: |  |
|  | *Name:*  Dr. Mark Fukuda |  |  |
|  | | | |
| Signed: |  | Date: |  |
|  | *Name:* Dr Sea Darapiseth |  |  |

| Signed: |  | Date: |  |
| --- | --- | --- | --- |
|  | *Name:*  Dr Sok Peou |  |  |
|  | | | |
| Signed: |  | Date: |  |
|  | *Name:* Dr. Bryan Smith |  |  |
|  | | | |
| Signed: |  | Date: |  |
|  | *Name:* Dr Chanthap Lon |  |  |
|  | | | |
| Signed: |  | Date: |  |
|  | *Name:* Dr Kurt Schaecher |  |  |
|  | | | |
| Signed: |  | Date: |  |
|  | *Name:* Dr. Paktiya Teja-Isavadharm |  |  |
|  | | | |
| Signed: |  | Date: |  |
|  | *Name:* Dr. Delia Bethell |  |  |
|  | | | |
| Signed: |  | Date: |  |
|  | *Name:* Dr. Wiriya Rutvisuttinunt |  |  |
|  | | | |
| Signed: |  | Date: |  |
|  | *Name:* Dr. David Saunders |  |  |
|  | | | |
| Signed: |  | Date: |  |
|  | *Name:* Mr Ses Sarim |  |  |

Statement of Compliance 1

Signature Page 1

List of Abbreviations 5

Protocol Summary 7

[2 Background Information and Scientific Rationale](../ARC2%20ver%201%205%2023%20April.doc) [16](#__RefHeading___Toc127958843)

[2.1 Background Information](../ARC2%20ver%201%205%2023%20April.doc) [16](#__RefHeading___Toc127958844)

[2.2 Rationale](../ARC2%20ver%201%205%2023%20April.doc) [29](#__RefHeading___Toc127958845)

[2.3 Potential Risks and Benefits](../ARC2%20ver%201%205%2023%20April.doc) [31](#__RefHeading___Toc127958846)

[2.3.1 Potential Risks](../ARC2%20ver%201%205%2023%20April.doc) [31](#__RefHeading___Toc127958847)

[2.3.2 Potential Benefits](../ARC2%20ver%201%205%2023%20April.doc) [31](#__RefHeading___Toc127958848)

[3 Objectives](../ARC2%20ver%201%205%2023%20April.doc) [33](#__RefHeading___Toc127958849)

[4 Study Design](../ARC2%20ver%201%205%2023%20April.doc) [34](#__RefHeading___Toc127958850)

[5 Study Population](../ARC2%20ver%201%205%2023%20April.doc) [36](#__RefHeading___Toc127958851)

[5.1 Selection of the Study Population](../ARC2%20ver%201%205%2023%20April.doc) [36](#__RefHeading___Toc127958852)

[5.2 Study Site 36](#_Study_Site)

[5.3 Inclusion Criteria](../ARC2%20ver%201%205%2023%20April.doc) [36](#__RefHeading___Toc127958853)

[5.4 Exclusion Criteria](../ARC2%20ver%201%205%2023%20April.doc) [37](#__RefHeading___Toc127958854)

[5.5 Randomization Procedures](../ARC2%20ver%201%205%2023%20April.doc) [38](#__RefHeading___Toc127958855)

[6 Study Procedures/Evaluations](../ARC2%20ver%201%205%2023%20April.doc) [39](#__RefHeading___Toc127958856)

[6.1 Study Procedures](../ARC2%20ver%201%205%2023%20April.doc) [39](#__RefHeading___Toc127958857)

[6.2 Laboratory Evaluations](../ARC2%20ver%201%205%2023%20April.doc) [39](#__RefHeading___Toc127958858)

[6.2.1 Laboratory Evaluations/Assays](../ARC2%20ver%201%205%2023%20April.doc) [39](#__RefHeading___Toc127958859)

[6.2.2 Special Assays or Procedures](../ARC2%20ver%201%205%2023%20April.doc) [40](#__RefHeading___Toc127958860)

[6.2.3 Specimen Collection, Preparation, Handling and Shipping](../ARC2%20ver%201%205%2023%20April.doc) [41](#__RefHeading___Toc127958861)

[7 Study Schedule](../ARC2%20ver%201%205%2023%20April.doc) [44](#__RefHeading___Toc127958862)

[7.1 Screening](../ARC2%20ver%201%205%2023%20April.doc) [44](#__RefHeading___Toc127958863)

[7.2 Follow-up and Final Visits](../ARC2%20ver%201%205%2023%20April.doc) [44](#__RefHeading___Toc127958864)

[7.3 Compensation 45](#_Compensation)

[7.4 Criteria for Discontinuation or Withdrawal of a Subject](../ARC2%20ver%201%205%2023%20April.doc) [45](#__RefHeading___Toc127958865)

[7.5 Reseach-related Injuries](../ARC2%20ver%201%205%2023%20April.doc) [45](#__RefHeading___Toc127958865)

[8 Assessment of Outcome Measures](../ARC2%20ver%201%205%2023%20April.doc) [46](#__RefHeading___Toc127958866)

[8.1 Specification of the Appropriate Outcome Measures](../ARC2%20ver%201%205%2023%20April.doc) [46](#__RefHeading___Toc127958867)

[8.1.1 Primary Outcome Measures (Clinical Outcomes)](../ARC2%20ver%201%205%2023%20April.doc) [46](#__RefHeading___Toc127958868)

[8.1.2 Primary Outcome Measures (Laboratory Outcomes)](../ARC2%20ver%201%205%2023%20April.doc) [47](#__RefHeading___Toc127958869)

[8.1.3 Secondary Outcome Measures](../ARC2%20ver%201%205%2023%20April.doc) [47](#__RefHeading___Toc127958870)

[9 Safety assessment and reporting](../ARC2%20ver%201%205%2023%20April.doc) [48](#__RefHeading___Toc127958871)

[9.1 Definition of Adverse Event (AE)](../ARC2%20ver%201%205%2023%20April.doc) [48](#__RefHeading___Toc127958872)

[9.2 Definition of Serious Adverse Event (SAE)](../ARC2%20ver%201%205%2023%20April.doc) [48](#__RefHeading___Toc127958873)

[9.3 Safety Reporting Procedures](../ARC2%20ver%201%205%2023%20April.doc) [49](#__RefHeading___Toc127958874)

[9.3.1 Serious Adverse Event Detection and Reporting](../ARC2%20ver%201%205%2023%20April.doc) [49](#__RefHeading___Toc127958875)

[9.3.2 Type and Duration of the Follow-up of Subjects After Adverse Events](../ARC2%20ver%201%205%2023%20April.doc) [51](#__RefHeading___Toc127958876)

[9.4 Halting Rules](../ARC2%20ver%201%205%2023%20April.doc) [51](#__RefHeading___Toc127958877)

[10 Clinical Monitoring Structure](../ARC2%20ver%201%205%2023%20April.doc) [54](#__RefHeading___Toc127958878)

[10.1 Site Monitoring Plan](../ARC2%20ver%201%205%2023%20April.doc) [54](#__RefHeading___Toc127958879)

[10.2 Protocol Deviations](../ARC2%20ver%201%205%2023%20April.doc) [54](#__RefHeading___Toc127958880)

[11 Statistical Considerations](../ARC2%20ver%201%205%2023%20April.doc) [55](#__RefHeading___Toc127958881)

[11.1 Study Outcome Measures and Analysis](../ARC2%20ver%201%205%2023%20April.doc) [55](#__RefHeading___Toc127958882)

[11.2 Sample Size Considerations](../ARC2%20ver%201%205%2023%20April.doc) [55](#__RefHeading___Toc127958883)

[11.3 Participant Enrollment and Follow-Up](../ARC2%20ver%201%205%2023%20April.doc) [56](#__RefHeading___Toc127958884)

[12 Access to Source Data/Documents](../ARC2%20ver%201%205%2023%20April.doc) [57](#__RefHeading___Toc127958885)

[13 Quality Control and Quality Assurance](../ARC2%20ver%201%205%2023%20April.doc) [59](#__RefHeading___Toc127958886)

[14 Ethics/Protection of Human Subjects](../ARC2%20ver%201%205%2023%20April.doc) [60](#__RefHeading___Toc127958887)

[14.1 Declaration of Helsinki](../ARC2%20ver%201%205%2023%20April.doc) [60](#__RefHeading___Toc127958888)

[14.2 Institutional Review Board](../ARC2%20ver%201%205%2023%20April.doc) [60](#__RefHeading___Toc127958889)

[14.3 Informed Consent](../ARC2%20ver%201%205%2023%20April.doc) [61](#__RefHeading___Toc127958890)

[14.3.1 Informed Consent Process](../ARC2%20ver%201%205%2023%20April.doc) [62](#__RefHeading___Toc127958891)

[14.4 Subject Confidentiality](../ARC2%20ver%201%205%2023%20April.doc) [62](#__RefHeading___Toc127958892)

[14.5 Future Use of Stored Specimens](../ARC2%20ver%201%205%2023%20April.doc) [63](#__RefHeading___Toc127958893)

[14.6 Compensation](../ARC2%20ver%201%205%2023%20April.doc) [63](#__RefHeading___Toc127958893)

[15 Literature References](../ARC2%20ver%201%205%2023%20April.doc) [64](#__RefHeading___Toc127958894)

SUPPLEMENTS/APPENDICES

A: Study Schedule

B: Roles and Responsibilities

C: Safety Monitoring Committee Charter

ABBREVIATIONS

| ACPR | Adequate Clinical and Parasitological Response |
| --- | --- |
| ACT | Artemisinin-based Combination Therapy |
| AE | Adverse Event |
| ANC | Absolute neutrophil count |
| AS | Artesunate |
| AV | Atrioventricular |
| CFR | Code of Federal Regulations |
| CIOMS | Council for International Organizations of Medical Sciences |
| CRF | Case Report Form |
| CRO | Clinical Research Organization |
| CRR | Continuing Review Report |
| DHA | Dihydroartemisinin |
| DHSP | Division of Human Subjects Protection |
| DNA | Deoxyribonucleic Acid |
| DOT | Directly Observed Therapy |
| EKG | Electrocardiogram |
| ETF | Early Treatment Failure |
| FCT | Fever Clearance Time |
| FDA | Food and Drug Administration |
| FWA | Federal-Wide Assurance |
| GCP | Good Clinical Practice |
| GCT | Gametocyte Clearance Time |
| GLP | Good Laboratory Practice |
| GMP | Good Manufacturing Practice |
| Hb | Hemoglobin |
| hCG | Human Chorionic Gonadotropin |
| HIV | Human Immunodeficiency Virus |
| HRP2 | Histidine-rich Protein II |
| HRPO | Human Research Protection Office |
| HSRRB | Human Subjects Research Review Board |
| HURC | Human Use Review Committee |
| IC | Inhibitory Concentration |
| ICF | Informed Consent Form |
| ICH | International Conference on Harmonization |
| IEC | Independent or Institutional Ethics Committee |
| IRB | Institutional Review Board |
| ISM | Independent Safety Monitor |
| IV | Intravenous |
| LCF | Late Clinical Failure |
| LPF | Late parasitological failure |
| LTF | Late treatment failure |
| M5AS | Mefloquine 1250 mg plus artesunate |
| MEF | Mefloquine |
| MOP | Manual of procedures |
| MTD | Maximum tolerated dose |
| N | Number (typically refers to subjects) |
| NIH | National Institutes of Health |
| ORP | Office of Research Protection |
| P | Plasmodium |
| PCR | Polymerase chain reaction |
| PCT | Parasite Clearance Time |
| Pf | Plasmodium falciparum |
| PK | Pharmacokinetic |
| PI | Principal Investigator |
| QC | Quality Control |
| QT | Q-T Interval |
| RBC | Red Blood Cell |
| RNA | Ribonucleic Acid |
| SAE | Serious Adverse Event |
| SMC | Safety Monitoring Committee |
| SOP | Standard Operating Procedure |
| SSP | Study Specific Procedure |
| WBC | White Blood Cell |
| WHO | World Health Organization |

**Protocol Summary**

**Title**: Artemisinin Resistance in Cambodia II

**Protocol Identifier**: WRAIR #1396, HSRRB Log Number A-14479, RPC252

**Sponsor**: Armed Forces Research Institute of Medical Sciences

**Funding:** Bill and Melinda Gates Foundation Program Grant through WHO/Geneva and the US DoD Global Emerging Infections Surveillance (GEIS) Program

**Rationale**: Once it develops and spreads, resistance to artemisinin derivatives, currently the most essential antimalarial drugs for the treatment of *Plasmodium falciparum* malaria, could very well be the most devastating event in the history of malaria control in the 21^st^ century. There is an urgent need for early detection and to investigate recent reports of treatment failures with advanced artemisinin combination therapies as well as artesunate monotherapy along Cambodia’s western borders. Recent data indicate reduced overall sensitivity of *P. falciparum* to artemisinin derivatives both *in vivo* as well as *in vitro* along the Cambodian-Thai border. In our recent ARC1 study (“Artemisinin Resistance in Cambodia 1”, WRAIR #1296, HSRRB A-13922) conducted in 2006/7 at the same study location as this current protocol, individual isolates were detected that are highly suggestive of resistance to artemisinins. ARC1, which compared an experimental regimen of 4 mg/Kg of oral artesunate for 7 days (28 mg/Kg total dose) versus a standard comparator regimen of oral quinine and tetracycline, found 4 patients in the artesunate arm who had re-emergence of *P. falciparum* parasites during 28 days of follow-up; 3 were classified as LPF and 1 as a LCF. This study also found that a worrying 22% of subjects in the artesunate arm were still parasitemic after 72 hours; the median PCT in the 4 patients who later recrudesced was 97.6 hours compared to 52.2 hours in the patients who were cured. Malaria parasites collected from the two subjects who were ultimately found to meet all of the *a priori* criteria for artemisinin resistance were also found to have a reduced susceptibility to DHA *in vitro* with IC_50_s of 14.0 and 14.4 nM as compared to a mean value of 3.34 nM for the other isolates. This protocol is a follow-up study to ARC1. The aim of this project is to determine whether regimens with increased artesunate doses can overcome the problem of reduced drug sensitivity to artemisinins and to determine whether these experimental regimens are safe and well tolerated.

**Objectives**: PRIMARY:

- To determine the impact of varying doses of artesunate on treatment outcome and whether higher doses of artesunate can overcome the problem of compromised artemisinin sensitivity in the region.
- To determine the safety and tolerability of this previously untested experimental high dose (6 mg/Kg/D X 7 day, total 42 mg/Kg) artesunate monotherapy regimen.

SECONDARY:

- Validate treatment response parameters (PCT, FCT, GCT and the proportion of patients still parasitemic on Day 3) for their role in predicting failures.
- To further evaluate current malaria *in vitro* drug susceptibility in this area.
- To validate potential genetic markers of artemisinin resistance and the role of identification of low level parasitemia by PCR

**Study Design**: Randomized, open label study

**Population**: 150 evaluable subjects randomized in 3 groups (3 artesunate monotherapy arms, ratio 2:1:2). Otherwise healthy *P. falciparum* infected malaria patients aged 18 to 65 years.

**Study Location**: Battambang Province, Cambodia

**Study Site**: Tasanh Health Center

**Study Drugs**: Artesunate monotherapy for 7 days.

| Treatment Group | Sample Size | Artesunate | |
| --- | --- | --- | --- |
|  |  | Dosing  mg/kg/day | Total Dose/kg |
| 1 ”Standard” artesunate regimen | 60 | 2 | 14 |
| 2 ARC 1 artesunate regimen | 30 | 4 | 28 |
| 3 Experimental, ‘high-dose’ artesunate regimen | 60 | 6 | 42 |

**Study Duration**: Approximately 18 months from enrollment of the first subject. The study may be repeated the following year after amendment of the protocol. An amendment will have to be reviewed and approved by all reviewing IRBs.

**Subject Duration**: Duration per subject is 42 days

**Endpoints**: Primary clinical outcome is cure (Adequate Clinical and Parasitological Response – ACPR as defined by WHO criteria) on Day 28 and 42. Secondary outcome measures are time until parasite, fever, and gametocyte clearance (PCT, FCT, and GCT).

**Abstract**: A total of 150 volunteers with acute uncomplicated falciparum malaria will be randomly assigned one of 3 arms to be treated with artesunate monotherapy for 7 days at a ratio of 2:1:2. The study design will be based on the WHO recommendations for the ‘Assessment and Monitoring of Antimalarial Drug Efficacy for the Treatment of Uncomplicated Falciparum Malaria’ (WHO, 2003). Study participants will be otherwise healthy malaria patients aged 18 to 65 years with uncomplicated falciparum malaria recruited in Battambang Province, Cambodia.

The artesunate will be administered orally (a single dose of 2, 4, or 6 mg/kg/day) over a total duration of 7 days (Day 0-6) with directly observed therapy.

Patients will be admitted to the hospital for the duration of study drug administration or until all signs and symptoms of malaria have disappeared, whichever comes first. After Day 6 they will be followed as Outpatients until Day 42 with scheduled follow-up visits on Day 14, 21, 28, 35, and 42.

Primary clinical outcome is cure (Adequate Clinical and Parasitological Response - ACPR) on Day 28 and 42 and the safety and tolerability of this previously untested experimental high dose (6 mg/Kg/D X 7 day, total 42 mg/Kg) artesunate monotherapy regimen. Secondary outcome measures are time until parasite, fever, and gametocyte clearance (PCT, FCT, and GCT). Parasite genotyping will be used to distinguish recrudescence from reinfection by PCR. Subjects will be monitored for clinical adverse events throughout the study duration.

Blood will be drawn on the day of admission (before initiating therapy) for *in vitro* drug sensitivity testing and for PCR (markers of drug resistance and to distinguish recrudescence from reinfection by genotyping). Malaria smears will be prepared regularly until parasite clearance and again at follow up visits on Days 14, 21, 28, 35, and 42 and whenever symptoms consistent with malaria appear. Plasma samples for determining drug levels will be obtained on the first and last day of therapy. Study participation for each individual will be 42 days.

# Key Roles

**Individuals**: **Principal Investigators:**

*Mark Fukuda, M.D., LTC, MC*

*Dept. of Immunology and Medicine*

*Armed Forces Research Institute of Medical Sciences (AFRIMS)*

*315/6 Rajvithi Road, Bangkok 10400, Thailand*

*Tel. 66-2-644-5775, Fax 66-2-644-4784*

*Email: mark.fukuda@afrims.org*

*Duong Socheat, M.D.*

*National Center for Parasitology, Entomology and Malaria Control*

*Office: #372, Monivong Blvd.*

*Phnom Penh, Cambodia*

*P.O. box 1062*

*Tel.: 855 23 211 926, Fax: 855 23 211 926*

*Email: socheatd@cnm.gov.kh*

*Youry Se, M.D., M.P.H.M.*

*Armed Forces Research Institute of Medical Sciences (AFRIMS)*

*No. 2. Kim Yl Sung Boulevard*

*Khan Tuol Kork, Phnom Penh, Cambodia*

*Tel: 855 (0) 12 992-029*

*Email: youry@online.com.kh*

**Investigators:**

*Sok Peou M.D.*

*Technical Bureau Staff of CNM*

*National Center for Parasitology, Entomology*

*and Malaria Control Program,*

*Ministry of Health, Cambodia (CNM)*

*Nº 27 B2 , St 656, Khan Toul Kork,*

*Phnom Penh, Cambodia*

*Tel: 855 12 889 189*

*Email: sokpeou6@yahoo.com*

*Sea Darapiseth M.D.*

*Technical Officer of CNM*

*National Center for Parasitology, Entomology*

*and Malaria Control Program,*

*Ministry of Health, Cambodia (CNM)*

*Office: #372, Monivong Blvd.,,*

*Phnom Penh, Cambodia*

*Tel: 855 16 804525*

*Email: seadarapiseth@gmail.com*

*Bryan Smith, M.D., LTC, MC*

*Dept. of Immunology and Medicine*

*Armed Forces Research Institute of Medical Sciences (AFRIMS)*

*315/6 Rajvithi Road, Bangkok 10400, Thailand*

*Tel. 66-2-644-5775, Fax 66-2-644-4784*

*Email: bryan.smith@afrims.org*

*and Division of Experimental Therapeutics, WRAIR, Washington D.C.*

*Kurt Schaecher, Ph.D., MAJ, MS*

*Dept. of Immunology and Medicine*

*Armed Forces Research Institute of Medical Sciences (AFRIMS)*

*315/6 Rajvithi Road, Bangkok 10400, Thailand*

*Tel. 66-2-644-5775, Fax 66-2-644-4784*

*Email: schaecher.kurt@afrims.org*

*Paktiya Teja-Isavadharm, Ph.D.*

*Dept. of Immunology and Medicine*

*Armed Forces Research Institute of Medical Sciences (AFRIMS)*

*315/6 Rajvithi Road, Bangkok 10400, Thailand*

*Tel. 66-2-644-5775, Fax 66-2-644-4784*

*Email: paktiyat@afrims.org*

*Delia Bethell, B.M. BCh, M.A., M.R.C.P.C.H.*

*Dept. of Immunology and Medicine*

*Armed Forces Research Institute of Medical Sciences (AFRIMS)*

*315/6 Rajvithi Road, Bangkok 10400, Thailand*

*Tel. 66-2-644-5775, Fax 66-2-644-4784*

*Email: delia.bethell@afrims.org*

*Wiriya Rutvisuttinunt, Ph.D.*

*Dept. of Immunology and Medicine*

*Armed Forces Research Institute of Medical Sciences (AFRIMS)*

*315/6 Rajvithi Road, Bangkok 10400, Thailand*

*Tel. 66-2-644-5775, Fax 66-2-644-4784*

*Email: wiriyar@afrims.org*

*Lon Chan Thap, M.D., M.C.T.M.*

*Armed Forces Research Institute of Medical Sciences (AFRIMS)*

*No. 2. Kim Yl Sung Boulevard*

*Khan Tuol Kork, Phnom Penh, Cambodia*

*Tel: 855 92 666 959*

*Email: chanthapl@afrims.org*

*David Saunders, M.D.*

*Dept. of Immunology and Medicine*

*Armed Forces Research Institute of Medical Sciences (AFRIMS)*

*315/6 Rajvithi Road, Bangkok 10400, Thailand*

*Tel. 66-2-644-5775, Fax 66-2-644-4784*

*Email: david.saunders@afrims.org*

*Ses Sarim*

*Director HC Tasanh*

*Or Tortem, Tasanh Commune,*

*Sam Lot District,*

*Battambang, Cambodia*

*Tel: 855 12 412 749*

**Medical Monitor:**

*Koy Lenin, M.D.*

*Battambang Referral Hospital*

*Emergency and ICU unit*

*Dongkorteap village, Tuol Ta Ek Commun,*

*Battambang district, Battambang province*

*Tel: 85 53 730 100*

*Fax: 86 53 953 223*

**Site Monitor:**

*Ms. Mali Ittiveerakul, RN*

*Quality Assurrance Personnel*

*Dept. of Immunology and Medicine*

*Armed Forces Research Institute of Medical Sciences (AFRIMS)*

*315/6 Rajvithi Road, Bangkok 10400, Thailand*

*Tel. 66-2-644-5775, Fax 66-2-644-4784*

*Email: malii@afrims.org*

**Clinical Research Coordinator:**

*Miss Sabaithip Sriwichai, RN*

*Dept. of Immunology and Medicine*

*Armed Forces Research Institute of Medical Sciences (AFRIMS)*

*315/6 Rajvithi Road, Bangkok 10400, Thailand*

*Tel. 66-2-644-5775, Fax 66-2-644-4784*

*Email: sabaithips@afrims.org*

**Laboratory Coordinator:**

*Mr. Sittidech Surasri*

*Medical Technologist*

*Dept. of Immunology and Medicine*

*Armed Forces Research Institute of Medical Sciences (AFRIMS)*

*315/6 Rajvithi Road, Bangkok 10400, Thailand*

*Tel. 66-2-644-5775, Fax 66-2-644-4784*

*Email: sittidechs@afrims.org*

*Mr Montri Arsanok*

*Medical Technologist*

*Dept. of Immunology and Medicine*

*Armed Forces Research Institute of Medical Sciences (AFRIMS)*

*315/6 Rajvithi Road, Bangkok 10400, Thailand*

*Tel. 66-2-644-5775, Fax 66-2-644-4784*

*Email: montria@afrims.org*

**Institutions**: *Armed Forces Research Institute of Medical Sciences (AFRIMS)*

*Dept. of Immunology and Medicine*

*315/6 Rajvithi Road, Bangkok 10400, Thailand*

*Tel. 66-2-644-5775; Fax 66-2-644-4784*

*National Center for Parasitology, Entomology and Malaria Control*

*#372, Monivong Blvd., Phnom Penh, Cambodia*

*Tel.: +855 23 211 926; Fax.: +855 23 996 202*

**Laboratories**: *Armed Forces Research Institute of Medical Sciences (AFRIMS)*

*Dept. of Immunology and Medicine*

*315/6 Rajvithi Road, Bangkok 10400, Thailand*

*Tel. 66-2-644-5775; Fax 66-2-644-4784*

*AFRIMS-CNM Study Site Laboratory Center*

*Tasanh Health Center*

*Or Tortem, Tasanh Commune*

*Sam Lot District, Battambang, Cambodia*

*Tel: 855 12 889 189, 855 12 532 225*

*Center for Vaccine Development
University of Maryland School of Medicine
685 West Baltimore Street, HSF1-480
Baltimore, MD 21201, USA
Tel: 1-410-706-2491 / 3082
Fax: 1-410-706-1204*

*University of South Florida*

*3720 Spectrum Blvd*

*Tampa, FL 33612, USA*

*Tel: 1-813 974 1273*

*Fax: 1-813 974 0992*

**Institutional Review Boards:**

*WRAIR IRB*

*Walter Reed Army Institute of Research*

*Office for Human Research Protections (OHRP)*

*Tel: +1301 619-2165, Fax: +1301 619-7803*

*E-mail: hsrrb@det.amedd.army.mil*

*National Ethics Committee for Health Research*

*Hlth IRB# 1 (FWA# 00010451, IRB # 00003143)*

*#2 Kim Il Sung Blvd, Khan Tuol Kok, Phnom Penh. Cambodia*

*Tel.: 855 23 880-345, Fax: 855 23 880-346*

*E-mail: research03@nchads.org.*

*Research Ethics Review Committee of the World Health Organization*

*Avenue Appia 20, 1211 Geneva 27, Switzerland*

*Tel +41 22791 2111, Fax. +41 22791 3111*

*http://www.WHO.INT/RPC/RESEARCH_ETHICS*

# Background Information and Scientific Rationale

## Background Information

In 2006/2007 we conducted the ARC1 study (Artemisinin Resistance in Cambodia 1, WRAIR #1296, HSRRB A-13922), which used an integrated *in vivo – in vitro* approach with the aim of investigating reports of emerging artemisinin resistance in Cambodian patients with uncomplicated falciparum malaria. Preliminary data from this study suggest that along parts of the Cambodian-Thai border there are individual *P. falciparum* isolates, which are be highly resistant to artemisinins. Although the prevalence of these isolates was low, the overall sensitivity of the parasite isolates was significantly reduced as compared to western Thailand, an area with relatively high levels of antimalarial drug resistance, but at the same time an area from which so far there are no reports of increased numbers of failures with artemisinin-based combination therapies (ACTs). In ARC1 some individual isolates were associated with greatly increased parasite clearance times, treatment failures despite 7 days of artesunate monotherapy (4mg/kg), and very high inhibitory concentrations for artemisinins *in vitro*.

In that study the 28-day cure rate in the 4 mg/Kg/Day X 7 days artesunate monotherapy arm (n=60) was 93.3 % (95% CI: 83.8-98.2) vs. 100% (89.7-100%) in the control group (quinine – tetracycline for 7 days following the national treatment guidelines in Cambodia). Mean PCT in the 4 patients who failed artesunate monotherapy was almost twice that of those who were cured (97.6 vs. 52.2 hrs). *In vitro* drug susceptibility tests indicate significantly higher geometric mean IC_50_s for artemisinins as compared to western Thailand and Bangladesh. Patients who failed therapy had IC_50_ values up to 5 times higher than the overall mean. Drug levels measured 90 and 150 minutes after drug intake on day 0 were used to define individual DHA levels. Of the 4 patients who failed therapy, two had satisfactory drug levels while the other two did not. Although some failures may therefore be linked to inadequate drug levels, at least 2 patients (3.3%; 95% CI: 0.4-11.5) with the highest artemisinin IC_50_s and PCTs of 133 and 95 hrs failed therapy in spite of adequate drug levels suggesting clinically significant resistance to artemisinins.

Once it develops and spreads, resistance to artemisinin derivatives, currently the most essential antimalarial drugs for the treatment of *Plasmodium falciparum* malaria, could very well be the most devastating event in the history of malaria control in the 21st century (Noedl 2005). Forty countries have officially adopted artemisinin-based combination therapy (ACT) for the treatment of malaria since 2001. Twenty alone have adopted the combination of artemether and lumefantrine as their first- or second-line treatment for *P. falciparum* malaria. Artemisinin and its derivatives are the most potent and rapidly acting antimalarial drugs. They reduce the infecting malaria parasite biomass by roughly 10^4^ per cycle (White et al. 1999).

Currently used ACTs are starting to fail on both sides of the Cambodian-Thai border. In a recent study the efficacy of the combination of artesunate and mefloquine was reduced to only 79.3% with a 42-day follow-up in Pailin Province, in close proximity to our study site (Denis et al. 2006). Recent reports also suggest increasing numbers of failures with artemisinin combination therapies along the eastern borders of Thailand (Trat Province – data not PCR corrected), potentially due to artemisinin resistance (Vijaykadga et al. 2006). The authors report 21.4% failures in Trat Province with M5AS, a combination of 25 mg/kg mefloquine and 12 mg/kg of oral artesunate (as compared to only 3.4% in Tak province, along the Thai-Myanmar border, an area that is infamous for its high levels of drug resistance).

A possible explanation for higher failure rates in Thailand as compared to many other countries using artemisinin-based combination therapies could be the administration of artesunate as short course over the first 2 days of treatment only. Although the total dose of artesunate (600 mg in adults) used in Thailand is adequate and comparable to most other malaria-endemic nations the drug is administered over only 2 days. Together with the short half-life of artesunate (and its main metabolite dihydroartemisinin) this results in a shorter exposure of the parasites to the drug. However, the effectiveness of artemisinins in reducing the parasite biomass is proportional to the parasite generations that are exposed to the study drug. As the life cycle of P. falciparum is approximately 48 hrs, a 2-day regimen results in only one generation of parasites being exposed to the drug and naturally reduces the efficacy of the drug. However, this cannot explain higher failure rates in eastern as compared to western Thailand as seen in these recent studies.

New ACTs are constantly being developed for the treatment of uncomplicated falciparum malaria. However, even recently introduced ACTs (such as the combination of artemether and lumefantrine) in this region seem to be affected by high failure rates, either due to absorption problems or drug resistance (Denis et al. 2006b).

The existence of artemisinin resistance is a much-discussed issue. Suspected clinical artemisinin resistance was reported from Thailand, India, and Sierra Leone as early as the late 1990s (Luxemburger et al. 1998; Gogtay NJ,et al. 2000; Sahr et al. 2001). Isolated *in vitro* resistance has been reported from numerous countries, particularly in and around Southeast Asia, and strains showing decreased *in-vitro* susceptibility have been obtained in laboratories by intermittently exposing malaria parasites in culture to rising drug concentrations (Meshnick 2002). However, the significance of many studies reporting artemisinin resistance may be questionable as most reports are limited to either clinical or in vitro data. Generally measuring the clinical impact of antimalarial drug resistance is difficult, and resistance might not be recognized until it is severe. This is partly because routine health information systems may grossly misjudge the magnitude of the problem (Breman 2001). In spite of the fact that few regional drug resistance meetings pass without at least one country reporting either *in vitro* or *in vivo* artemisinin resistance, so far there is no convincing evidence for its existence.

Further evaluation of the suspected cases of artemisinin resistance will require a combined *in vivo - in vitro* strategy and dose ranging to assess whether reduced drug sensitivity can be overcome by increased doses of artesunate. The only reasonable approach is a careful analysis of clinical treatment response parameters, not just cure rates, combined with *in vitro* drug sensitivity data. The latest generation of *in vitro* assays is highly sensitive and permits the testing of almost any fresh *Plasmodium falciparum* sample directly from the patient, without major selection bias, cryopreservation, or pre-culturing, procedures that could significantly alter the intrinsic drug sensitivity pattern. One of the biggest problems in distinguishing artemisinin resistance from resistance to the partner drugs is the fact that artemisinins are generally used in combination with drugs that have a longer half life. The reason is that even in areas with high sensitivity to artemisinin, due to their short half life, artemisinin derivatives would have to be administered for at least 6-7 days to achieve close to 100% cure. This would make compliance in a routine outpatient setting very difficult.

# Artesunate for the treatment of malaria

Artesunate has the chemical name (3R,5aS,6R,8aS,9R,10S,12R,12aR)-Decahydro-3,6,9-trimethyl-3,12-epoxy-12H-pyrano[4,3-j]1,2-benzodioxepin-10-ol,hydrogen succinate. Artesunate is a semisynthetic, water soluble, derivative of artemisinin, an antimalarial compound isolated from the Chinese herb Qinghao (Artemisia annua). Artemisinin is a sesquiterpene lactone that bears a peroxide grouping and, unlike most other antimalarials, lacks a nitrogen-containing heterocyclic ring system. Artesunate is the hemisuccinate ester, synthesized by reacting dihydroartemisinin (DHA) and succinic acid anhydride in alkaline medium. This type of reaction invariably yields an ester linkage in alpha configuration. Artesunate is rapidly metabolized to DHA, which is also an active antimalarial, in the body.

As early as 1979, there were several reports on the efficacy of artemisinin against *P. falciparum* and *P. vivax* in over 2000 Chinese patients. It was shown to produce more rapid parasite clearance as compared to other antimalarials and to be highly effective also in chloroquine resistant strains of *P. falciparum*. However, the rate of early recrudescence was high, which was claimed to be due partly to its poor solubility in water and oils and finally led to the development of better soluble derivatives such as the methyl ether derivative (artemether) and the hemisuccinate ester (artesunate) (Karbwang & Na-Bangchang 1993). Chinese physicians tested artemisinin in 6000 patients in the course of the 1970s and the summaries of these studies were finally published in 1982 by the China Cooperative Research Group. At the same time they also reported the first studies with the most important artemisinin derivatives artemether and artesunate in an animal model (Li et al., 1994). It is licensed for use in oral or intravenous preparations throughout Southeast Asia and many African and South American countries, and is first-line therapy for treatment of multi-drug resistant falciparum malaria. The oral drug formulation is on the WHO Essential Drug list. Artesunate is produced by Guilin Pharmaceutical Factory, Guangxi, China and repackaged by Atlantic Laboratories Corp., Ltd., Bangkok, Thailand. The latter is produced under GMP standards. Many million doses have been administered to humans worldwide.

**Artesunate: Animal Studies**

Artesunate was shown to be highly active against a number of malaria species in animal models. The activity of artesunate and DHA was measured against the erythrocytic forms of *P. falciparum, P. berghei, P. knowlesi, and P. coatneyi* in mice or monkeys. In a preliminary study conducted in immunocompromised mice infected with the T24 strain (chloroquine and quinine resistant) of *P. falciparum*, a complete clearance of parasites from the blood was observed on Day 2 of treatment with DHA (50 mg/kg for 2 days) by the oral route. Microscopic observations at 24 hours showed predominantly pycnotic forms (76%), some altered trophozoites (10%), few trophozoites (1%), and schizonts (3%). At 48 hours, only pycnotic forms were observed. Chloroquine and quinine were not effective in clearing the parasitemia in mice infected with the T24 strain. Treatment with chloroquine induced minimal alterations in morphology (11 - 16% pycnotic forms). However, against a chloroquine sensitive strain (NF54) morphological changes after treatment with chloroquine were similar to that of DHA against the chloroquine-resistant strain. The activity of artesunate was not measured.

In mice infected with *P. berghei* (173N strain) and treated with intravenous artesunate or chloroquine, a 50% and 90% reduction in parasitemia was observed by 18 to 24 hours, and 24 to 30 hours, respectively. Recrudescence was observed on Day 28 in mice treated with artesunate (110 mg/kg) for 5 days by the intravenous route. However, chloroquine (14.9 mg/kg for 5 days) completely cured the mice on Day 28. In another study, mice infected with another strain of *P. berghei* (ANKA strain) showed complete cure on Day 60 after intramuscular treatment with 56 mg/kg artesunate. Complete clearance of the parasitemia was observed within 2 days of treatment. Similar observations were made with DHA. The variation in the activity of artesunate in the different studies may be due to the different strains of *P. berghei* used for infection, the route of drug administration or severity of infection.

In monkeys infected with *P. knowlesi*, the intravenous administration of artesunate (10 mg/kg for 7 days) reduced the parasitemia by 90% at 13 hours. The parasite clearance time (PCT) was 42 hours and all 3 monkeys remained negative for 28 days of observation. However, at the lower dose (3.16 mg/kg) the PCT was 40 hours and, 1/3 monkeys showed recrudescence on Day 15. At a higher dose (31.6 mg/kg), the mean parasite clearance time was 56 hours and no recrudescence was observed. Quinine at 10 mg/kg dose was effective in reducing the parasitemia by 50% in 3.3 hours, however, a 90% reduction of parasitemia was not attained. At a higher dose (31.6 mg/kg), the mean time to parasite clearance was 104 hours and recrudescence was observed 2 to 10 days after parasite clearance.

In another study, normal and splenectomized monkeys infected with *P. coatneyi* were treated with artesunate. The clearance of parasitemia was slower in splenectomized animals compared to normal controls. The reduction in parasitemia at 24 hours in the splenectomized and non-splenectomized animals was 86% and 99%, respectively. Infected erythrocytes showed ultrastructural changes such as enlargement of food vacuoles and ribosomal clumping 4 hours after administration of artesunate. Recently, splenectomized rhesus macaques were tested in a severe malaria model of *P. coatneyi* infection with intravenous artesunate at AFRIMS (RS Miller, unpublished data 2006). Rapid parasite clearance was achieved with optimal dosing noted at 8mg/kg (equivalent to 2.4 mg/kg IV in humans), but recrudescence occurred after many days.

In animal tests, artemisinin compounds are less toxic than quinoline antimalarials. In an oral artesunate study in rats submitted to the FDA for review of rectal artesunate, 3- and 7-day regimens (with a total human equivalent doses of 36 mg/kg) produced no deaths or neurohistologic lesions (M. Gomez, personal communication 2006). Lethal doses of these compounds in rodents cause multiple system toxicity with bone marrow depression, diarrhea and hemoglobinuria. Sublethal doses produce transient depression of reticulocyte count. 7-day toxicity studies in rhesus macaques found a no effect dose of 8 mg/kg when artesunate was administered for 7 days intravenously. Diarrhea occurred in a dose-dependent fashion at higher doses, usually started after 4-5 days of drug administration. Hemoglobinuria was occasionally seen at 32 mg/kg/day. In lab tests, transient reversible reticulocytopenia was noted at all doses, and elevated alkaline phosphatase was noted at higher doses. Segment 1 reprotoxicity studies in mice reveal no effects on fertility. Segment II studies reveal fetal loss and resorption, particularly during organogenesis in the first trimester.

Animal studies have revealed neurotoxicity in some members of the artemisinin class. Administration of high doses (20 mg/kg/d) of artemether and arteether produces neurotoxicity in rats, dogs and rhesus (gait disturbances, loss of spinal pain responses, restlessness, tremor and incoordination, followed by respiratory depression, convulsions and cardiac arrest) with characteristics brainstem lesions seen on neurohistopathology (Brewer 1994). Results from recent studies demonstrate distinct differences in the ability of artemisinin derivatives to produce neurotoxicity, with fat-soluble derivatives (arteether and artemether) showing much greater propensity to cause these effects. Whereas arteether (25 mg/kg) treated mice showed distinct behavioral changes due to neurotoxicity, behavioral performance was not significantly affected in any rats treated with artesunate (31 mg/kg) (Genovese 2000). The significantly different safety profiles of these drugs may also have to do with the route of administration. Recent studies show that there was no pathologic evidence of neuronal death in mice receiving either oral artemether, or oral or intramuscular artesunate, in doses up to 300 mg/kg/day (Nontprasert et al. 2002). The WHO GLP rat study, IV doses as high as 20 times human equivalent doses, did not produce any clinical neurotoxicity or histologic neuropathology. Likewise, the rhesus study of IV artesunate in Thailand did not reveal any neuropathologic lesions at doses up to 128 mg/kg IV for 7 days (up to 18 times usual human doses). In conclusion, the favorable chemical and pharmacokinetic properties of artesunate suggest that neurotoxicity is not a major concern with this compound.

**Artesunate: *In Vitro***

A number of studies conducted in Southeast Asia show that *P. falciparum* parasites remain highly susceptible to artesunate and DHA *in vitro* in most areas, resulting in mean 50% inhibitory concentrations (IC_50_) for artesunate of 1.24 ng/ml in Vietnam (Wongsrichanalai et al. 1997), 0.35 ng/ml in the Philippines (Bustos et al. 1994), and 0.98 ng/ml in Thailand respectively (Noedl et al. 2004). Although the IC50s found in different regions of the world showed considerable variations in their levels of sensitivity, until very recently there was little evidence towards a developing artemisinin-resistance (Wongsrichanalai et al., 1999; Ringwald et al., 1999). Recent *in vitro* as well as *in vivo* data from Thailand and other Southeast Asian countries however suggest increasing numbers of failures and higher IC50s.

# Artesunate: Efficacy and Safety Profile

Artesunate (AS) has been licensed for the treatment of malaria in many Southeast Asian countries since the 1990s. The drug is well absorbed, and rapidly hydrolyzed in the liver to the more active metabolite, dihydroartemisinin. Its half-life is less than 1 hour, so that all biological activity is gone after an oral dose within 12 hours (Teja-Isavadharm et al., 2001). While this drug is currently still considered to be the most potent blood schizonticide available, this rapid clearance requires repeated dosing of artesunate in order to clear all metabolically susceptible parasites. Generally artemisinin derivatives (artesunate and artemether) are extremely well tolerated when used at therapeutic dosage (Price et al. 1999).

Data from 23 trials with 1891 patients (Hien and White 1993) comparing artemisinin derivatives with other antimalarials showed shortening of fever clearance time compared to intravenous quinine and parasite clearance time by 17 and 32% respectively. Artesunate appeared to have more rapid action than the other derivatives. No serious toxicity was observed in these trials. 600 mg at 0 h and 4 h followed by 400 mg at 24, 32, 48 and 56 h was compared with oral quinine 1500 mg daily in 3 divided doses over 14 consecutive days, in patients with acute malaria. Shorter parasite and fever clearance times occurred in the artemisinin group but 50% of patients recrudesced compared with 23% in the quinine group. Monotherapy with artesunate is ineffective when given for 3 days or less. Li (1994) summarized the early Chinese data, reporting a recrudescence rate of 51% when artesunate monotherapy was administered for 3 days (total dose 280 mg-400mg) to 65 adults with uncomplicated falciparum malaria. Shorter regimens (total dose AS 600mg) given over 1-2 days did not cure any volunteers in Thailand (Bunnag et al. 1991). A subsequent randomized controlled trial by Li (1997) showed 39% recrudescence rate at day 28 with 400mg given over 3 days.

Extending the duration of treatment with artesunate has significantly improved the efficacy. Li (1994) reported a recrudescence rate at day 28 of 5 % when artesunate (Guilin) was given for 5 days (total dose of 440mg-600mg) to 144 patients with uncomplicated falciparum malaria in China, and 7% recrudescence in his subsequent study (600 mg over 5 days). Studies in Thailand using 600mg over 5 days confirmed these findings with 84% cure rates at day 28 in 167 volunteers (Looareesuwan et al. 1994). A study conducted by the same group suggests cure rates of 98 to 100% for 7 and 5 days respectively of artesunate treatment when using overall doses of 1200 to 1600 mg of artesunate (Looareesuwan et al. 1997). The treatment was well tolerated and found to be safe. These studies led to more extensive combination testing with other antimalarials, which now have become standard practice. In our ARC1 study, conducted in 2006/7 in Battambang Province Cambodia, artesunate 4 mg/kg/day for 7 days (total dose 1400 mg) was well tolerated with no artesunate toxicity demonstrated and no SAEs.

## Artesunate Safety Data

Price and colleagues (1999) reviewed the cumulative experience of the Shoklo Malaria Research Unit, Thailand, with over 3500 cases of artemisinin derivatives, mostly oral artesunate. Vomiting occurred in 2.2% of those receiving artemisinins alone the first day, with a significant risk associated with prior vomiting or nausea before enrollment (OR=2.8 and 2.1, respectively). The incidence of vomiting fell with subsequent days.

No seizures were reported in artesunate monotherapy groups, but 15 seizures following treatment with mefloquine and artesunate/artemether (incidence 177/100,000). No urticaria, hemoglobinuria, or neuropsychiatric reactions occurred in AS monotherapy treated persons (n=836). Lab tests showed no significant changes in WBC or neutrophil counts, although platelets did drop in 2 of 154 volunteers (AS + MEF) during treatment, with resolution by day 14. No renal dysfunction occurred in any patient, but 4.5% experienced some increase in liver transaminases with no clinical hepatic dysfunction (all co-treated with mefloquine). No significant changes were seen on EKG during therapy, including tests 1 hour after artesunate administration. Case reports with artesunate in persons with malaria have rarely recorded bradycardia, 1^o^ AV block or QT prolongation, but all are flawed by concurrent malaria which can cause these findings due to hypocalcemia.

A neurologic exam (consisting of fine-finger dexterity, heel-toe walk, hearing assessed by tuning fork, eye exam for nystagmus and Rombergs test) was performed on admission, day 2, day 7 and day 28 in 1,664 volunteers receiving artesunate (Price et al. 1999). Dizziness was the most common complaint, with exam evidence of disturbed balance in 5 of 690 treated with artemisinins alone. All signs and symptoms resolved by day 7. No one developed deafness or permanent neurologic injury. A multicentre trial conducted in Africa in 941 children (age 10 years or older) with randomly assigned amodiaquine plus artesunate or amodiaquine and placebo suggests that artesunate combination therapy is also safe in children (Adjuik et al. 2002). A combination of artesunate with sulfadoxine-pyrimethamine was found to be safe, well tolerated, and efficacious in children with uncomplicated falciparum malaria in The Gambia (Doherty et al. 1999; von Seidlein et al.2000). A case-control study from Thailand failed to detect any evidence of significant neurotoxicity in 79 patients treated previously with oral artemether or artesunate for acute malaria (Van Vugt 2000), and Phase IV monitoring by the WHO has failed to reveal neurotoxicity in any humans treated with artesunate (M Gomes, personal communication 2006).

A prospective trial conducted in Thailand in 461 pregnant women suggested that artesunate was well tolerated and that birth outcomes did not differ significantly to community rates for abortion, stillbirth, congenital abnormality, and mean gestation at delivery (McGready et al. 2001). An earlier study conducted by the same group found no congenital abnormality in any of the newborn children whose mothers were treated with artesunate for falciparum malaria during pregnancy, and that all children followed for more than one year developed normally (McGready et al.1998). Nonetheless, based on the animal data, the drug is contraindicated in the first trimester of pregnancy.

Below is a summary of human and animal safety, toxicity, and tolerability data obtained from studies of higher artesunate doses than those that have been typically used in routine clinical practice. To date, there has been one reported death that was due to a symptom complex that could have been consistent with an artesunate overdose of one 18 Kg child who received 22 mg/Kg of artesunate rectally for 4 days (total 88 mg/Kg) for a *P. vivax* infection (Campos 2008). While the symptom complex was consistent with possible artesunate toxicity, it is important to point out that other potential etiologies, including primaquine toxicity, were not conclusively ruled out; also that the pharmacodynamic properties of rectally dosed artesunate are extremely variable and that it is possible that a depot-like effect accentuated the artesunate duration of effect and toxicity.

*Table 1*: Summary of human and animal safety, toxicity, and tolerability data with Artesunate.

| **Species/**  **Study** | **Route of admin** | **Dose, mg/kg/day** | **Length of treatment, days** | **Total dose, mg/kg** | **HED- IV/day** | **Disease Status** | **Toxicity** |
| --- | --- | --- | --- | --- | --- | --- | --- |
| **Human** |  |  |  |  |  |  |  |
| ARC 2 | PO | 6 | 7 | 42 | 3.6 | Pf | Unknown |
| AS overdose case report (child)* | PR | 22 | 4 | 88 | ??? | Pv | Ataxia, confusion, multisystem failure, death |
| ARC 1 (#1296) | PO | 4 | 7 | 28 | 2.4 | Pf | No sign. |
| IV AS 1b | IV | 8 | 3 | 24 | 8 | Healthy | No sign., mild ⇓ ANC |
| DRT (#1263) | IV | 4.8 | 3 | 14.4 | 4.8 | Pf | No sign. |
| **Rhesus** |  |  |  |  |  |  |  |
|  | IV | 128 | 1 | 128 | 32 | Healthy | Transient neuro/hemo toxicity= MTD |
|  | IV | 16 | 7 | 112 | 4 | Healthy | Mild diarrhea and ⇓ rbc |
|  | IV | 32 | 7 | 224 | 8 | Healthy | ⇓ Activity, RBC, ANC; mild diarrhea |
|  | IV | 128 | 7 | 512 | 32 | Healthy | Moderate ⇓ activity, hematuria, bloody diarrhea⇒ death |
|  |  |  |  |  |  |  |  |
| **Beagle** | IV | 20-50 | 14 | 20-50 | 10-25 | Healthy | Transient ⇓ RBC |
|  |  |  |  |  |  |  |  |
| **Rat** | IV | 240 | 1 | 240 | 34 | Healthy | MTD |

Key: DRT = IV artesunate dose-ranging trial, HED = human-equivalent dosing, ANC = absolute neutrophil count, RBC = red blood cells, MTD = maximum tolerated dose, PV = *Plasmodium vivax* malaria, PR = per rectum

References: *Campos et al, 2008; IB for IV Artesunate for Severe Malaria, IND 64,769, ver 2.0

**Laboratory Safety Data from the ARC 1 (1296) Study**

Laboratory safety data collected from the ARC 1 study consisted of Complete Blood Counts by the manual method and a finger stick blood glucose. The results of these test showed:

**Table 2.1: Red Blood Cell (RBC) in 10^6^ cells/µl of both arms**

|  | Artesunate | | |  | Quinine and Tetracycline | | |
| --- | --- | --- | --- | --- | --- | --- | --- |
|  | Day 0 | Day 3 | Day 7 |  | Day 0 | Day 3 | Day 7 |
| N | 73 | 73 | 71 |  | 35 | 35 | 35 |
| Mean | 4.31 | 4.50 | 4.25 |  | 4.41 | 4.49 | 4.58 |
| SD | 0.75 | 0.76 | 0.68 |  | 0.78 | 0.75 | 0.68 |
| 95%CI(u) | 4.14 | 4.33 | 4.09 |  | 4.14 | 4.23 | 4.34 |
| 95%CI (l) | 4.49 | 4.68 | 4.41 |  | 4.67 | 4.75 | 4.81 |
| Median | 4.15 | 4.35 | 4.2 |  | 4.44 | 4.6 | 4.47 |
| IQR (u) | 4.8 | 5.15 | 4.8 |  | 4.95 | 4.95 | 5.25 |
| IQR (l) | 3.8 | 3.95 | 3.76 |  | 3.75 | 3.8 | 4.05 |
| Minimum | 3.02 | 2.7 | 2.53 |  | 2.52 | 2.5 | 3.35 |
| Maximum | 6.1 | 6.23 | 5.85 |  | 5.8 | 5.85 | 5.69 |

**Table 2.2: Comparison RBC between treatment arms**

Comparisons between treatment arms: parametric (t-test) / non-parametric (Kruskall-Wallis):

|  | | Quinine and Tetracycline | | |
| --- | --- | --- | --- | --- |
|  |  | Day 0 | Day 3 | Day 7 |
| Artesunate | Day 0 | 0.55 / 0.43 |  |  |
|  | Day 3 |  | 0.95 / 1.0 |  |
|  | Day 7 |  |  | 0.02 / 0.03 |

**Table 2.3: White Blood Cell (WBC) in cells/µl of both arms**

|  | Artesunate | | |  | Quinine and Tetracycline | | |
| --- | --- | --- | --- | --- | --- | --- | --- |
|  | Day 0 | Day 3 | Day 7 |  | Day 0 | Day 3 | Day 7 |
| N | 73 | 73 | 71 |  | 35 | 35 | 35 |
| Mean | 6364 | 6190 | 6894 |  | 6476 | 6077 | 7853 |
| SD | 2216 | 1962 | 1995 |  | 1746 | 1624 | 1859 |
| 95%CI(u) | 5847 | 5732 | 6422 |  | 5877 | 5519 | 7214 |
| 95%CI (l) | 6881 | 6648 | 7366 |  | 7076 | 6635 | 8491 |
| Median | 6325 | 5850 | 6600 |  | 6375 | 6050 | 7600 |
| IQR (u) | 7600 | 7500 | 8510 |  | 7500 | 7000 | 8510 |
| IQR (l) | 4800 | 4700 | 5250 |  | 5550 | 4500 | 6300 |
| Minimum | 2500 | 3050 | 3000 |  | 3050 | 4000 | 4100 |
| Maximum | 7500 | 12600 | 11450 |  | 10300 | 10100 | 11000 |

**Table 2.4: Comparison WBC between treatment arms**

Comparisons between treatment arms: parametric (t-test) / non-parametric (Kruskall-Wallis):

|  | | Quinine and Tetracycline | | |
| --- | --- | --- | --- | --- |
|  |  | Day 0 | Day 3 | Day 7 |
| Artesunate | Day 0 | 0.79 / 0.52 |  |  |
|  | Day 3 |  | 0.77 / 0.95 |  |
|  | Day 7 |  |  | 0.02 / 0.02 |

**Table 2.5: Hematocrit (Hct in %) of both arms**

|  | Artesunate | | |  | Quinine and Tetracycline | | |
| --- | --- | --- | --- | --- | --- | --- | --- |
|  | Day 0 | Day 3 | Day 7 |  | Day 0 | Day 3 | Day 7 |
| N | 73 | 73 | 71 |  | 35 | 35 | 35 |
| Mean | 37 | 36 | 36 |  | 38 | 38 | 39 |
| SD | 5.3 | 5.1 | 5.3 |  | 5.3 | 4.9 | 4.5 |
| 95%CI(u) | 35.8 | 35.3 | 34.6 |  | 36.2 | 35.8 | 37.7 |
| 95%CI (l) | 38.2 | 37.7 | 37.1 |  | 39.8 | 39.2 | 40.8 |
| Median | 38 | 36 | 35 |  | 38 | 37 | 39 |
| IQR (u) | 41 | 40 | 40 |  | 42 | 42 | 43 |
| IQR (l) | 33 | 33 | 32 |  | 35 | 33 | 36 |
| Minimum | 26 | 24 | 23 |  | 25 | 30 | 29 |
| Maximum | 47 | 46 | 53 |  | 48 | 47 | 49 |

**Table 2.6: Comparison Hct between treatment arms**

Comparisons between treatment arms: parametric (t-test) / non-parametric (Kruskall-Wallis):

|  | | Quinine and Tetracycline | | |
| --- | --- | --- | --- | --- |
|  |  | Day 0 | Day 3 | Day 7 |
| Artesunate | Day 0 | 0.35 / 0.37 |  |  |
|  | Day 3 |  | 0.32 / 0.40 |  |
|  | Day 7 |  |  | 0.002 / 0.001 |

**Table 2.7 blood glucose of both arms**

|  | Artesunate | | |  | Quinine and Tetracycline | | |
| --- | --- | --- | --- | --- | --- | --- | --- |
|  | Day 0 | Day 3 | Day 7 |  | Day 0 | Day 3 | Day 7 |
| N | 73 | 73 | 73 |  | 35 | 35 | 35 |
| Mean | 97.15 | 84.39 | 85.54 |  | 95.43 | 82.60 | 73.17 |
| SD | 19.36 | 15.84 | 14.01 |  | 20.81 | 18.63 | 16.16 |
| 95%CI(u) | 92.63 | 80.67 | 82.20 |  | 88.28 | 76.20 | 67.62 |
| 95%CI (l) | 101.67 | 88.11 | 88.87 |  | 102.58 | 89.00 | 78.72 |
| Median | 97 | 83 | 84 |  | 93 | 79 | 70 |
| IQR (u) | 106 | 89 | 92 |  | 110 | 94 | 78 |
| IQR (l) | 85 | 76 | 77 |  | 79 | 69 | 61 |
| Minimum | 61 | 58 | 65 |  | 64 | 57 | 53 |
| Maximum | 189 | 180 | 160 |  | 141 | 129 | 119 |

**Table 2.8: Comparison blood glucose between treatment arms**

Comparisons between treatment arms: parametric (t-test) / non-parametric (Kruskall-Wallis):

|  | | Quinine and Tetracycline | | |
| --- | --- | --- | --- | --- |
|  |  | Day 0 | Day 3 | Day 7 |
| AS | Day 0 | 0.67 / 0.59 |  |  |
|  | Day 3 |  | 0.61 / 0.37 |  |
|  | Day 7 |  |  | 0.0001/ 0.0001 |

The results of these laboratory assessments showed that over 7 days of treatment the mean HCT fell by 1, the mean RBC count fell by 0.06 and the mean WBC rose by 530 in AS-treated patients. In QT-treated patients the mean HCT rose by 1, the mean RBC count rose by 0.17 and the mean WBC rose by 1377. None of these changes were significantly different from baseline. There are no differences in any of the laboratory parameters between AS and QT treatment groups on Days 0 or 3. The mean RBC, WBC and HCT in AS-treated patients were significantly lower on Day 7 than those in QT-treated patients on Day 7. QT-treated patients had significantly lower mean plasma glucose levels on Day 7 compared to AS-treated patients.

None of the observed laboratory findings were clinically significant nor led to discontinuation of the study drug. However since all patients had uncomplicated malaria the observed differences between treatment groups seen on Day 7 were probably a drug effect. AS is known to depress the compensatory rise in reticulocytes in response to acute malaria and this may have led to the observed differences in HCT and RBC seen on Day 7. Similarly, quinine is known to have an insulin-like hypoglycemic effect that may account for the observed lower mean plasma glucose values seen on Day 7.

**Standards of health care for acute uncomplicated *P. falciparum* malaria**

Standard of care (first-line therapy) for uncomplicated *P. falciparum* malaria is the combination of artesunate and mefloquine (Table 3).

*Table 3:* The combination of artesunate with mefloquine is the official first line therapy for adults in Cambodia; A+ M5 (artesunate + mefloquine, for 3days: artesunate 50 mg tablets and mefloquine 250 mg tablets (August 2007).

| Weight | Age | Number of Tablets | | | | | |
| --- | --- | --- | --- | --- | --- | --- | --- |
|  |  | Artesunate (50mg) | | | Mefloquine (250mg) | | |
|  |  | Day 1 | Day 2 | Day 3 | Day 1 | Day 2 | Day 3 |
| >35kg | >15 years | 4 | 4 | 4 | 2 | 2 | 1 |

The combination of quinine with tetracycline is the official second line therapy for adults in Cambodia. Both drugs are administered in split dose every 8 hours over 7 days (Table 4). In spite of the known side effects, particularly of quinine, this regimen is considered to be safe and highly efficacious.

*Table 4: Dosage of quinine (30mg/kg/24h) 300 mg tablets and tetracycline (25mg/kg/24h) 250 mg capsules for 7 days*

| Age | Weight  (kg) | Quinine | | | Tetracycline | | |
| --- | --- | --- | --- | --- | --- | --- | --- |
|  |  | Dose/8h | Total/day | Total/7days | Dose/8h | Total/day | Total/7days |
| <6 months | < 7 | 1/4 | 3/4 | 5^1/4^ | - | - | - |
| 6m–2 years | 7-15 | 1/2 | 1^1/2^ | 10^1/2^ | - | - | - |
| 2-8 years | 16-30 | 1 | 3 | 21 | - | - | - |
| 9-15 years | 31-45 | 1^1/2^ | 4^1/2^ | 31½ | 1^1/2^ | 4^1/2^ | 31½ |
| >15 years | >45 | 2 | 6 | 42 | 2 | 6 | 42 |

**Study area**

In the year 2003, 71,258 malaria cases were reported from Cambodia to the World Health Organization reported (WHO 2005). However, the real number of cases could well be significantly higher. Malaria is still a major threat in Cambodia, particularly in the hilly forested environments and forest fringes. The number of reported malaria cases in Cambodia has decreased gradually between 1993 and 2003. However, in 2003 the reported number of treated cases, severe cases and deaths as well as the case fatality rate slowly started to increase again. This may at least in part be explained by the rising numbers reports of treatment failures with advanced artemisinin combination therapies.

Of particular concern in Cambodia is the high level of multidrug resistance present in affected areas. Strains of *P. falciparum* are resistant to most antimalarial drugs, and the quality and usage pattern of antimalarial drugs are suboptimal. Recent studies show that counterfeit and substandard drugs are frequent in Cambodia, especially quinine and artesunate. Furthermore, a survey of antimalarial drug use in 2002 showed problems of delayed treatment-seeking behavior, widespread use of many antimalarial drugs for the same malaria episode and non-adherence to malaria treatment (WHO 2005).

**Study site**

Tasanh Health Center is located in Battambang Province in western Cambodia in close proximity to the Thai border. Battambang Province is an area particularly affected by malaria. In 2007 Battambang reported more malaria cases than any other province in Cambodia. Tasanh Health Center alone reported around 600 cases of laboratory confirmed malaria in 2007 (Tables 5 and 6). The catchment area of Tasanh Health Center is around 20 km surrounding Tasanh town and includes two smaller health centers that refer patients to Tasanh Health Center. Critically, the Tasanh Health Center and its referral health centers stands in the middle of the crucial area of the growing reports of increasing rates of ACT failure and the possible emergence of artemisinin tolerance or resistance. It is literally only a few kilometers from the Thai-Cambodia border and south of the crucial town of Pailin where other similar studies are underway. The number of staff at the health center is 25 and includes personnel trained by AFRIMS in research and GCP that participated in the earlier, highly successful ARC1 study in 2006/2007. In this study, 111 patients with acute *P. falciparum* malaria were enrolled, of whom two thirds received artesunate monotherapy (4mg/kg daily x 7 days) and one third received quinine-tetracycline therapy; subjects stayed at the study site for 21 days under supervision of the study team; there were no deaths and no SAEs reported. To our knowledge, there is no better-trained or equipped staff and facility within this critical geographic area of intense interest that would be more capable of carrying out this research effort. If required, intensive care and emergency services are available and have been coordinated for at the Battambang Provincial Hospital, approximately 1.5 hours drive from Tasanh. Potentially necessary medical supplies will be purchased and pre-positioned within the ICU, the hospital Director and staff will be briefed on this research effort and its results, and the Medical Monitor for this study is the Director of Emergency and ICU services for the hospital.

Table 5: Malaria cases treated at Tasanh Health Center, 2007 Cambodia (Outpatient department)

| Cases | 0-4 yrs | 5-14 yrs | 15-49 yrs | > 50 | Total |
| --- | --- | --- | --- | --- | --- |
| Malaria | 62 | 121 | 365 | 42 | 590 |

Table 6: Malaria cases treated at Tasanh Health Center, 2007 Cambodia (Inpatient department)

|  | 0-4 yrs | | 5-14 yrs | | 15-49 yrs | | > 50 yrs | | Total | |
| --- | --- | --- | --- | --- | --- | --- | --- | --- | --- | --- |
|  | Cases | Deaths | Cases | Deaths | Cases | Deaths | Cases | Deaths | Cases | Deaths |
| Uncomplicated Malaria | 33 | - | 80 | - | 217 | - | 25 | - | 355 | - |
| Severe Malaria | 4 | 0 | 7 | 0 | 10 | 0 | 2 | 0 | 23 | 0 |
|  |  |  |  |  |  |  |  |  |  |  |
| Total Hospital Admissions | 158 | 2 | 111 | 1 | 783 | 2 | 167 | 1 | 1219 | 6 |
| Percentage Malaria | 20.89% | 0.00% | 72.07% | 0.00% | 27.71% | 0.00% | 14.97% | 0.00% | 29.12% | 0.00% |

**Target study population**

Tasanh Health Center (Samlot District, Battambang Province)

- Ethnic composition: Khmer 98-99% and Vietnamese 1-2 %
- Typical living condition: relatively poor, majority farmers (corn, bean or peanut plantations
- Occupation: majority farmers (95%) and loggers.
- There are two different population groups living in the area:

a) Long term residents (living in the area for more than 5 years. Most of the own the land they are working on.

b) New residents, approximately 5-7% of the population in Samlot District. Most of them moved to the area in the past 1- 2 years, coming from other Eastern provinces in Cambodia, (such as Kampong Cham, Takeo, and Kandal Province). New residents mostly live on forestry, hunting or laborers. This is the group most affected by malaria. They also have poorer access the health care system and develop severe malaria more frequently than long term residents.

- The average annual income for an individual in Cambodia is approximately 1,800 USD (CIA, 2008).
- Level of education: Mostly primary and secondary school only, according to statistics most of the population over 18 years can read and write (95%).

**Access, availability and cost of medical care**

Access to medical care: the majority (98%) of the population in Samlot District will attend the Tasanh Health Center or other nearby health centers. Unlike the larger towns in Cambodia there are few private medical facilities. The Ministry of Public Health provides basic medical care at Tasanh Health Center and similar health care facilities for a small charge. There is a 0.12$ charge per visit for the OPD and a 5$ charge for In-patient care. Particularly poor patients may be treated free of charge. Neither private nor government health insurance is available, particularly not in rural areas.

For pregnant women antenatal health checks, with tetanus immunization and iron supplements if indicated, are offered monthly free of charge from 7 months. In Battambang Province the proportion of women delivering at a health care facility is 36%. The standard care for malaria pregnancy for uncomplicated *P. falciparum* malaria is quinine alone for 7 days (30 mg/kg/day in 3 divided doses, but not more than 1800mg = 6 tablets of 300mg) and the combination of artesunate and mefloquine (A+M5) for 2^nd^ and 3^rd^ trimester as in Table 1.

## Rationale

Recent data indicate reduced overall sensitivity of *P. falciparum* to artemisinin derivatives both *in vivo* as well as *in vitro* along the Cambodian-Thai border. In our recent ARC1 study (Artemisinin Resistance in Cambodia I) in this region individual isolates were detected that were be highly resistant to artemisinins. Reports from the Ministries of Public Health on both sides of the Thai-Cambodian border indicate increasing numbers of treatment failures with artemisinin-based combination therapies, currently the last line of defense against the spread of multidrug resistant malaria (Denis et al. 2006a; Vijaykadga et al. 2006). **This study will be conducted in the area and with the population most affected by treatment failures with artemisinin combination therapies.** Although failures with combination regimens do not necessarily indicate resistance to both combination partners, both drugs can contribute to the number of failures. Our data suggest that there may be individual parasite isolates highly resistant to artemisinins.

The proposed study will assess whether these failures are dose-dependent and can be overcome with increased doses of artesunate. Only the combination of clinical (*in vivo* studies) data with modern laboratory assays (*in vitro* drug sensitivity assays and PCR) allows for a clear distinction of the role of each individual drug for the reported treatment failures.

Currently the existence of artemisinin resistance as well as the potential impact and possibly strategies to overcome artemisinin resistance are much-discussed issues. Suspected clinical artemisinin resistance was reported from Thailand, India, and Sierra Leone as early as the late 1990s (Luxemburger et al. 1998; Gogtay NJ,et al. 2000; Sahr et al. 2001). Isolated *in vitro* resistance has been reported from numerous countries, particularly in and around Southeast Asia, and artemisinin-resistant strains have been obtained in laboratories by intermittently exposing malaria parasites in culture to rising drug concentrations (Meshnick et. al. 2002). However, the significance of many studies reporting artemisinin resistance is questionable as most reports are limited to either clinical or *in vitro* data.

Detecting and quantifying artemisinin resistance in its early stages will require a combined *in vivo - in vitro* strategy. The only reasonable approach is a careful analysis of clinical treatment response parameters, not just cure rates, combined with *in vitro* drug sensitivity data. The latest generation of *in vitro* assays is highly sensitive and permits the testing of almost any fresh *Plasmodium falciparum* sample directly from the patient, without major selection bias, cryopreservation, or pre-culturing, procedures that could significantly alter the intrinsic drug sensitivity pattern. One of the biggest problems in distinguishing artemisinin resistance from resistance to the partner drugs is the fact that artemisinins are generally used in combination with drugs that have a longer half life. The reason is that even in areas with high sensitivity to artemisinin, due to their short half life, when used alone artemisinin derivatives would have to be administered for at least 6-7 days to achieve close to 100% cure. This would make compliance in a routine Out-patient setting very difficult as patients tend to stop taking the drug once they start feeling better.

Historically artemisinin derivatives have been used alone to treat uncomplicated falciparum malaria with high cure rates. However, this requires artemisinin derivatives to be used for durations that exceed the time span for which a reasonably high compliance can still be expected (a maximum of 3 to 4 days). In a field setting artemisinin derivatives should not be used as a monotherapy, partly because monotherapy treatment regimens must be approximately twice as long as combination therapies to be effective and will therefore inevitably lead to compliance issues. The three artemisinin monotherapy arms will provide data on dose-dependent efficacy.

The principal aim of this study is therefore to assess whether treatment failures with artemisinins show a dose-dependent trend and whether they may be overcome with increased doses of artesunate.

## Potential Risks and Benefits

### Potential Risks

The subject may experience a brief moment of physical discomfort or pain during the finger prick procedure and the venipuncture and there is a possibility of bruising and/or infection at the site of the finger-prick or venipuncture.

Subjects may experience ‘transient bitter taste’ as a possible side effect from the study drug. Artesunate is generally very well tolerated but may occasionally cause mild abdominal symptoms and dizziness and changes in the laboratory and EKG parameters. While still well below the total dose that has caused significant side effects in animal safety testing, the 6 mg/Kg artesunate dose regimen is an experimental dose that has not been previously administered to humans. It is therefore not possible to rule out the possibility of toxicity or adverse effects at this dose. A Safety Monitoring Committee will be convened prior to study execution and will monitor closely the AE profiles of artesunate-treated patients with special emphasis on the 6 mg/kg cohort.

There is a risk that malaria may recur. All subjects will therefore be closely monitored and quickly treated should any signs or symptoms of malaria be detected.

### Potential Benefits

As subjects will be hospitalized for at least the first 7 days and then closely monitored for any signs and symptoms of malaria, they will have the benefit of close medical supervision. All doses of the study drugs will be administered under direct supervision by medical/nursing staff trained in drug administration, and any change in the course of their infection or any adverse experiences will be recognized and treated more rapidly than would normally occur if they were treated as outpatients in a government facility.

Subjects will be immediately treated for any reappearance of parasitemia that occurs. Subjects who fail initial therapy, based on parasitological parameters, will be treated with a standard regimen following the national treatment guideline that is known to be effective. In addition, while admitted to the hospital the subjects may be examined and treated for other concurrent illnesses. Subjects will also receive medical attention and appropriate standard medical care or referral should they become ill during the study.

An early detection of artemisinin resistance and the knowledge whether failures can be overcome by increasing the dosage of artemisinins will directly benefit the malaria control program and the population of Cambodia and will allow for adequate countermeasures to be taken to avert a global disaster. The National Malaria Program of Cambodia will benefit from human resource capacity building, access to modern laboratory methodology (particularly novel techniques for *in vitro* drug sensitivity testing of antimalarial drugs) and GCP training for government staff.

# Objectives

**3.1 PRIMARY OBJECTIVE:**

- The principal aim of this project is to determine the role of varying doses of artesunate for treatment outcome and whether higher doses of artesunate could overcome the problem of reduced artemisinin sensitivity in the region.
- To determine the safety and tolerability of this previously untested experimental high dose (6 mg/Kg/D X 7 day, total 42 mg/Kg) artesunate monotherapy regimen.

**3.2 SECONDARY OBJECTIVES:**

- Validate treatment response parameters (PCT, FCT, GCT, and the proportion of patients parasitemic on Day 3) for their role in predicting failures.
- To further evaluate the current malaria *in vitro* drug sensitivity situation along the Cambodian-Thai border.
- To validate potential genetic markers of artemisinin resistance and the role of identification of low level parasitemia by PCR

# Study Design

This is an open label, randomized study. The study design is largely based on the WHO recommendations for the ‘Assessment and Monitoring of Antimalarial Drug Efficacy for the Treatment of Uncomplicated Falciparum Malaria’ (WHO, 2003). Patients with acute uncomplicated falciparum malaria will be randomly enrolled in 3 arms: All patients in arm 1, 2, and 3 will receive artesunate monotherapy for 7 days (Table 7). The ratio of enrollment into the 3 groups will be 2:1:2. Arm 2 serves as a control and will serve as a bridge to the ARC 1 study performed in 2006/2007. Patients in Arm 1 will receive a relatively low “standard” dose, and patients in Arm 2 will receive the intermediate dose of 4 mg/kg that was used in the ARC1 study. Patients in Arm 3 will receive an experimental “high-dose” regimen. Currently available safety data extends to subjects who have received the 28 mg/Kg total dose over 7 days and to another study administering 8 mg/Kg/day for 3 days (total dose 24 mg/Kg). Subjects randomized into this study’s ‘high-dose’ Arm 3 will, therefore, receive a total dose that is higher than has been previously studied in humans.

Table 7: Artesunate dosing for treatment groups 1-3.

| Treatment Group | Sample Size | Artesunate | |
| --- | --- | --- | --- |
|  |  | Dosing  mg/kg/day x 7 days | Total Dose/kg |
| 1 (Artesunate, “standard” dose) | 60 | 2 | 14 |
| 2 (Artesunate, ARC1 dose) | 30 | 4 | 28 |
| 3 (Artesunate, experimental “high” dose) | 60 | 6 | 42 |

Patients will be admitted to the hospital to receive directly observed therapy and close safety monitoring for the duration of study drug administration (7 days) or until all signs and symptoms of malaria have disappeared, whichever is longer. Thereafter patients will regularly report for follow-up (at least on Days 14, 21, 28, 35, and 42). Blood will be drawn on the day of admission (before initiating therapy) for *in vitro* drug sensitivity testing and for PCR (markers of drug resistance and to distinguish recrudescence from reinfection by genotyping). Malaria smears will be prepared up to 8 times on Day 0, and then up to 4 times a day thereafter until parasite clearance, and on Days 14, 21, 28, 35, and 42 or whenever symptoms consistent with malaria appear. Plasma samples for determining anti-malarial drug levels will be obtained on Days 0 and 6. Over the entire study, up to approximately 59 ml of blood may be drawn by venipuncture from patients who do not fail treatment. An additional approximately 26 ml of blood will be drawn in case of treatment failure. The study duration for each individual patient will be 42 days.

All patients in group 1, 2, and 3 will receive artesunate monotherapy for 7 days for the treatment of their *P. falciparum* infection. Patients who develop early treatment failures, who fail to completely clear parasites by the end of the 7^th^ day or who have a recurrence of parasitemia any time until Day 42 will be treated with 1^st^ or 2^nd^ line therapy following the national treatment guidelines of Cambodia.

**Study drug:**

Artesunate

| Chemical name: | Artesunate has the chemical name (3R,5aS,6R,8aS,9R,10S,12R,12aR)-Decahydro-3,6,9-trimethyl-3,12-epoxy-12H-pyrano[4,3-j]1,2-benzodioxepin-10-ol,hydrogen succinate |
| --- | --- |
| Generic name: | Artesunate |
| Trade name: | Artesunate |
| Dosage form: | Tablet |
| Strength: | 50 mg |
| Batch/lot number | 071201 |
| Manufactory date and expiry date | Mfg. date: 07/12/2007  Exp.date: 12/2010 |
| Manufacturer: | Guilin Pharmaceutical Co,. Ltd.  Add: No. 17 Shanghai Road, Guilin Guangxi, China  Post code: 541002  Tel: (773) 3832783  Fax: (773) 3833116  [http://www.guilinpharma.com](http://www.guilinpharma.com/) |
| Quality: | WHO pre-qualify http://www.guilinpharma.com/english/magazine/Empolder_list.asp?Action=0&id=192 |
| Source: | Supplied by the World Health Organization |

# Study Population

## Selection of the Study Population

Subjects will be male and non-pregnant female patients (age 18 - 65 years), recruited by active or routine passive case detection at Tasanh Health Center and other health centers in Battambang Province, Cambodia, who present with acute uncomplicated falciparum (Pf) malaria. Uncomplicated falciparum malaria is defined as falciparum malaria in the absence of applicable available signs or symptoms consistent with severe malaria as defined by WHO (WHO 2000). Eighteen years is the age at which individuals in Cambodia can start to provide their own consent. No additional recruitment material will be used in patient recruitment. As part of the Cambodian malaria control program malaria cases are routinely detected by active case detection (i.e. teams that collect diagnostic blood smears in the field) and referred to the nearest treatment facility. Subjects diagnosed with uncomplicated Pf malaria upon evaluation will be verbally notified about the ongoing trial, and asked to participate if interested. Study personnel will screen all persons reporting that they want to take part in the study, give study information and informed consent to the subject and screen for inclusion/exclusion criteria. The patients will be given the choice of either receiving standard of care treatment (artemisinin-based combination therapy) for their malaria or participating in the study. If potential volunteers cannot read the consent form, study personnel will verbally review the study information/consent form with them and answer all questions.

## Study Site

The study will be performed in Battambang Province, Cambodia. In-patient care will be provided at Tasanh Health Center under direct supervision of the investigators. The local personnel are trained and have experience in recruiting and providing in-patient care to study subjects.

## Inclusion Criteria

Male and female subjects with a diagnosis of acute falciparum malaria meeting all criteria listed below may be included in the study:

1. Acute symptomatic falciparum malaria infection as determined by malaria smear with a parasite density of 1000 to 200,000 asexual parasites/μL as determined on the thick/thin screening smear with fever (defined as ≥37.5ºC), or reported history of fever within the last 48 hours.
2. Age: 18-65 years old
3. All females between the age of 18 and 50 are required to have a negative human chorionic gonadotropin (hCG) pregnancy test (urine). All females of childbearing potential (not surgically sterile, or less than two years menopausal) are required to use an acceptable method of contraception, such as implant, injectable, or oral contraceptive(s), if possible with additional barrier contraception, intrauterine device, sexual abstinence, or vasectomized partner, throughout the study.
4. Written informed consent obtained
5. Willing to stay under close medical supervision for the study duration of 42 days
6. Otherwise healthy Out-patients

## Exclusion Criteria

Subjects presenting with any of the following will not be included in the study:

1. Pregnant women, nursing mothers, or women of childbearing potential who do not use an acceptable method of contraception (as described in Inclusion Criteria, #3)
2. Mixed malaria infection on admission by malaria smear
3. A previous history of intolerance or hypersensitivity to the study drug artesunate or to drugs with similar chemical structures, such as artemether, artemisinin or dihydroartemisinin
4. History of malaria drug therapy administered in the past 30 days
5. Previous participation in this trial, or participation in any other studies involving investigational or marketed products, concomitantly or within 30 days prior to entry in the study
6. History of significant cardiovascular, liver or renal functional abnormality or any other clinically significant illness, which in the opinion of the investigator would place them at increased risk.
7. Symptoms of severe vomiting (no food or inability to take food during the previous 8 hours).
8. Signs or symptoms of severe malaria (adapted from WHO recommendations (2003): prostration, impaired consciousness, respiratory distress, convulsions, systolic blood pressure < 70 mm Hg, abnormal bleeding, severe anemia with hemoglobin <8 g/dL or HCT <24%, hyperparasitemia at >4% parasitized red blood cells).
9. Unable and/or unlikely to comprehend and/or follow the protocol

## Randomization Procedures

Subjects will be assigned unique identification codes (ARC08 followed by a 3 digit number) in order of inclusion and randomly assigned to either one of the artesunate groups. The ratio of enrollment into the three groups is 2:1:2. Randomization will not be stratified and will be done using random number tables in blocks of 10. A randomization list will be created prior to beginning the trial. Sealed individual envelopes that will be opened only on enrollment of each subject will be used. The study personnel enrolling the subject will therefore not know which group the patient will be assigned to until opening the envelope.

# Study Procedures/Evaluations

## Study Procedures

The detailed design is outlined below and in the Appendices. Patients will be admitted to the Tasanh Health Center for the duration of study drug administration or until all signs and symptoms of malaria have disappeared, whichever comes later. Thereafter patients will regularly report for follow-up at least on Days 14, 21, 28, 35, and 42.

At the screening/baseline visit, the study will be fully explained to the subjects and written informed consent obtained. Subjects will be assessed as to whether they meet the inclusion/exclusion criteria. Demographic details (age, sex, weight, height) and medical history and concomitant medication details will be recorded. Vital signs (pulse, blood pressure, respiratory rate, and temperature) will be taken. A physical exam will be performed and clinical signs and symptoms will be evaluated. Blood smears to confirm *P. falciparum* malaria will be performed. Blood samples will be collected following the schedule outlined below.

Patient response to therapy will be monitored by assessing clinical parameters at baseline (Day 0) and daily at least until peripheral blood smears are negative for parasites. Specific detailed attention will be paid to daily (or more frequently if required) specified evaluation of any emerging adverse events including known potential toxicities that have been observed in human or animal studies with artemisinins. During hospitalization patients will have malaria smears performed up to 8 times on Day 0 and then every 6 hours until the smears are negative on 2 successive occasions. Thereafter blood smears will be performed on Days 14, 21, 28, 35, and 42. A final physical examination and blood testing may be requested from subjects who decide to withdraw from the study.

## Laboratory Evaluations

### Laboratory Evaluations/Assays

- Hematology (hematocrit, RBCs, WBCs, platelets) on days 0, 3, 6, and 14 (or whenever clinically warranted) (2 ml per blood draw).
- Glucose on days 0 (or whenever clinically warranted) (2 drops per blood draw).
- ALT on days 0, 3, 6, and 14 (or whenever clinically warranted) (1 ml per blood draw)
- Urine pregnancy test: Urine beta-HCG test. All female subjects will undergo a pregnancy test at baseline. Pregnant women will not be eligible for entry into the study.
- Urine (approximately 5ml) will also be collected from all participants to be frozen for probable antimalarial drug screening on the day 0 sample at a later date.
- Peripheral blood smears up to 8 times on Day 0 from blood drawn for other reasons or by finger prick blood smear at 12 and 18 hours and then 4 times daily until negative on 2 successive occasions. Thereafter blood smears will be performed on Days 14, 21, 28, 35, and 42 :
  - To determine species of parasite
  - To quantify parasitemia

### Special Assays or Procedures

- Blood samples for parasite DNA molecular marker characterization, parasite transcriptional expression profiling, and *in vitro* drug sensitivity will be collected on Days 0 and in case of re-emergence of parasitemia.
  - Samples for parasite DNA molecular marker characterization (8 ml each) will be collected to determine re-infection from recrudescence and to define molecular markers of drug resistance.
  - Samples for existing standard as well as novel new compounds in various stages of the drug development process *in vitro* drug sensitivity testing (8 ml each) will be collected and will follow the routine established at AFRIMS (Noedl 2004, 2005, and novel methodology and compounds).
  - Samples (5 ml each) for parasite total RNA will also be collected on Days 0 and in case of failure.
  - Samples for real time PCR analysis (up to 4 drops of blood per sample) to detect parasite densities below the microscopic detection limit will be collected every time a blood smear is made. These samples will be used to quantify parasitemia below the current level of detection of microscopy.
- Plasma samples for determining antimalarial drug levels (2 ml of whole blood per blood draw) will be collected from all patients on Day 0 just before and 15, 30, 60 (±10 minutes), 2, 4, 6 and 8 hours (±30 minutes) after drug intake, and again on Day 6 just before and 2, 4 and 6 hours after the final dose of artesunate.
- Plasma samples for bioassay (2ml) to evaluate any significant pre-study plasma antimalarial activity will be collected on admission prior to dosing and again if parasites reappear within the 42 day follow-up period.

### Specimen Collection, Preparation, Handling and Shipping

#### Instructions for Specimen Preparation, Handling, and Storage

Stained thick and thin blood smears will be examined by two microscopists who are blinded to each other’s results and to the treatment status of the study subject. Two blood smears will be made for every assessment. Slide 1 will be stained immediately and examined by a microscopist. The slide will then be stored together with the unstained slide 2 to be reexamined at a later stage. Parasite densities will be calculated based on a count of parasites per 200 WBCs (thick film) or per 5000 RBCs (thin film). A total of 200 oil immersion fields will be examined on the thick film before a blood smear is considered negative. The final count will be determined by taking the geometric mean of the two counts. In case of a difference in results (positive/negative; species diagnosis) between the two microscopists, the blood smear will be re-examined by a third microscopist and the third reading will be accepted as the final result.

Blood samples for parasite DNA molecular marker characterization will be collected in EDTA tubes and stored frozen at approximately -20ºC or below.

Blood for parasite RNA will be collected into PaxGene tubes and stored at room temperature or below.

Blood samples for *in vitro drug* sensitivity testing will be stored refrigerated if needed and the *in vitro* drug sensitivity assays will be performed directly at the study site. The remaining blood will be stored in liquid nitrogen for future drug sensitivity analysis.

The whole blood drawn for the measurement of drug levels (PK) will be collected, centrifuged, separated plasma and frozen at approximately -20ºC or below until testing. The frozen plasma will be stored at the study site and will be transported to AFRIMS laboratory in Thailand for analysis.

Specimens collected during the study will be labeled with the participants study ID number. Specimens will be stored for approximately 20 years at the Armed Forces Research Institute of Medical Sciences in Bangkok.

#### Specimen Shipment

The tests will be performed on site (*in vitro* drug sensitivity assay, microscopy, hematology, ALT, glucose, and urine pregnancy test) or at AFRIMS in Bangkok (PCR, drug screening testing, bioassay and pharmacokinetics) or at Center for Vaccine Development University of Maryland School of Medicine for parasite DNA molecular marker characterization and parasite transcriptional expression profiling and University of South Florida recovered parasite culture and *in vitro* drug testing. Specimens will be assigned new ID number by AFRIMS laboratory coordinator before shipping to the both collaborating laboratories for analysis.

## Rescue Treatment and Emergent *P. vivax* Infections

### Rescue Treatment

Subjects who fail to complete their randomly assigned artesunate dosing regimen for safety, tolerability, or other reasons, will receive treatment according to the following regimens to ensure adequate treatment of their *P. falciparum* malaria:

• If <3 doses of artesunate successfully given, patients will receive quinine/tetracycline in the following manner:

• If 3-5 doses of artesunate have been successfully administered, the patients will receive either mefloquine 15 mg/Kg orally + a second Mefloquine 10 mg/Kg oral dose administered 12 to 24 hours later, or Quinine/Tetracycline to complete at least 7 days of adequate antimalarial therapy if there is a contraindication to MQ (i.e. possible neurologic toxicity as the reason for stopping the artesunate). This will complete a standard ACT regimen (artesunate-mefloquine) or alternate adequate treatment regimen (quinine-tetracycline) for these subjects.


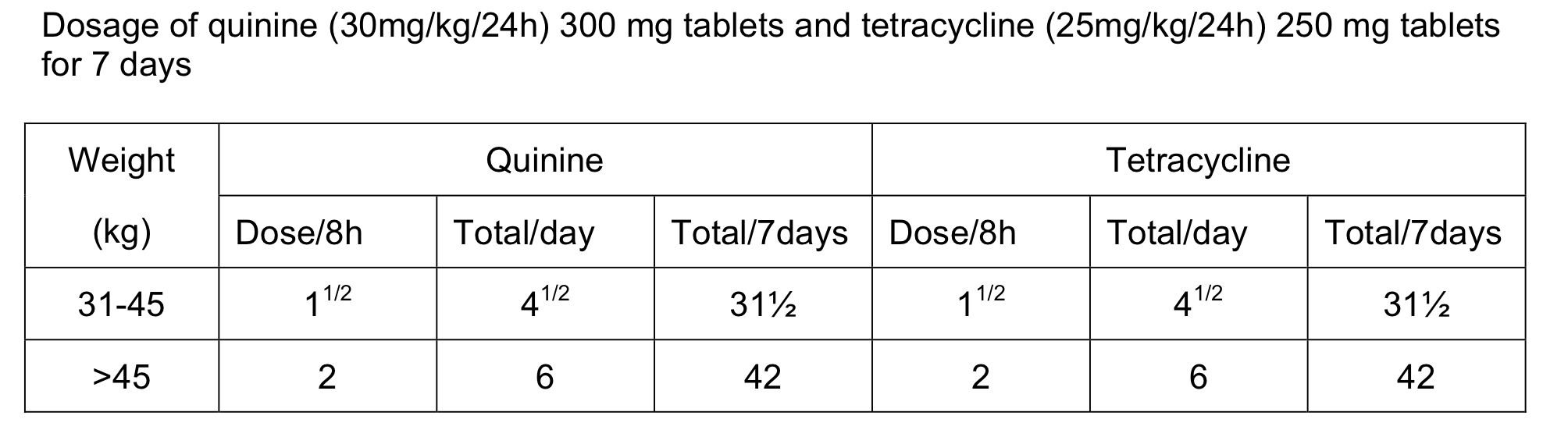


• If 6 or more doses of artesunate have been successfully administered, no further treatment is required.

All subjects receiving rescue treatment will continue to be followed until completion of their study periods (42 days) to ensure adequacy of treatment and appropriate safety follow-up.

### Treatment for emergent *P. vivax* infections

Enrolled subjects who develop *P. vivax* infection during the course of study follow-up will receive the usual Cambodian standard treatment for *P. vivax* consisting of oral chloroquine 25 mg/Kg divided over 3 days as well as treatment of *P. falciparum*. Currently primaquine is not recommended for radical cure within the Kingdom of Cambodia. These subjects will continue to be followed in the study until completion of their 42-day follow-up period for safety and efficacy.

# Study Schedule

Appendix A *Schedule of Procedures/Evaluations* contains the detailed schedule of observations and assessments to take place during the study.

## Screening

At the screening/baseline visit, the study will be fully explained to the subjects and written informed consent obtained from the subjects.

Subjects will be assessed as to whether they meet the inclusion/exclusion criteria. Patient who meet all criteria will be enrolled. Demographic details (age, sex, weight, height) and medical history and concomitant medication details will be recorded. Vital signs (pulse, blood pressure, respiratory rate, and temperature) will be taken. A physical exam will be performed and clinical signs and symptoms will be evaluated. Blood smears to confirm *P. falciparum* malaria will be performed. A blood sample will be taken for hematology, ALT and blood glucose analysis and a urine sample provided.

All female subjects between the age of 18 and 50 years will undergo a urine pregnancy test at baseline. They should either abstain from sexual relations or practice an acceptable method of contraception, such as an implant, injectable, or oral contraceptive(s). if possible with additional barrier contraception, intrauterine device, sexual abstinence, or vasectomized partner, throughout the study. Should pregnancy be suspected during the study the study subjects are advised to immediately notify the investigator. Pregnant women will not be eligible for entry into the study.

Blood samples will be drawn for parasite DNA molecular marker characterization and *in vitro* drug sensitivity analysis. Patient response to therapy will be monitored by assessing clinical parameters at baseline (Day 0) and daily at least until peripheral blood smears are negative for parasites. Plasma samples for determining drug levels will be collected from all patients at baseline and on Day 6*.*

## Follow-up and Final Visits

Patients will be admitted to the hospital (Tasanh Health Center) for the duration of study drug administration and followed closely with directly observed therapy and at least daily clinical assessment of their clinical condition and any potential emerging adverse events. They will remain in hospital for the full 7 days or until all signs and symptoms of malaria and/or any new gastrointestinal disturbances have disappeared, whichever is longer. Patients must regularly report for follow-up (at least on Days 14, 21, 28, 35, and 42) thereafter.

During hospitalization patients will have malaria smears performed up to four times a day until the smears are negative on 2 successive blood smears. Thereafter blood smears will be performed on Days 14, 21, 28, 35, and 42 or whenever signs and symptoms consistent with malaria reappear.

On Day 3, 6 and 14 hematology and ALT will be repeated (or whenever clinically warranted). Total RNA, *in vitro* and PCR samples will be collected in case of reemergence of parasitemia.

The time windows for the follow-up visits on Days 14, 21 and 35 are -2 to +6 days. Those on Days 28, and 42 are 0 to +6 days. Patients who do not attend the study site for follow-up will be contacted at their homes by study staff.

To maximize the scientific integrity of the study and patient safety, every effort will be made by study staff to contact patients missing follow up appointments. This may include telephone contact with subjects or visits to patient homes. Sample size calculations take into account a projected loss rate based on AFRIMS previous experience. Data and samples accrued from subjects subsequently lost to follow up will be stored and handled in the same fashion as the larger database including data from participants completing the study.

Patients who are found to have blood smears positive for *P. falciparum* malaria during follow-up who have received 6 or more artesunate doses will be treated with quinine/tetracycline according to national treatment guidelines for second-line treatment. These subjects will continue to be followed in the study until completion to ensure adequacy of treatment and completeness of their safety and tolerability follow-up.

## Compensation

During the first week patients will be provided with food, accommodation, and a daily compensation of 16,000 Riel (4000 Riel = 1 US Dollar). For the visits on Day 14, 21, 28, 35 and 42 patients will be provided 20,000 Riel for each visit and on Day 42 they will receive another 20,000 Riel for completing the entire study plus 4000 Riel for every day spent in the hospital. Patients will also be reimbursed for actual transportation costs for returning to the Health Center for follow-up patients. Patients completing the entire study and follow-up will receive a compensation of 260,000 Riel **(approximately $65)** plus reimbursement of actual transportation expenses. **This total reimbursement of about $5 per day is not felt to be high given that the subjects total time commitment to the study and follow-up leads to 13 days of lost work and compensation.**

## Criteria for Discontinuation or Withdrawal of a Subject

Any subject may be discontinued from the study at any time upon direction of the SMC, at the discretion of the investigator if he/she feels it is in the best interest of the subject (see Individual and Cohort Halting Rules, Sec 9.4.1-2) or if in the judgment of the investigator continuing in the study would be harmful and/or inappropriate for the subject (e.g. patients not tolerating the study drug, development of SAEs that require referral to another hospital) or if a patient cannot be followed thereby not permitting adequate safety assessment. Any patient who is terminated due to an SAE or unexpected AE will be reported to the medical monitor for review. Women who become pregnant during the trial will be taken off the study medicine and will be treated following the national treatment guidelines of Cambodia. If a woman becomes pregnant after the first 7 days she will be withdrawn form the study and will be followed for safety.

## Research-related Injuries

Medical care in case of research-related injury (including long-term medical care) will be provided free of charge according to local standard of care at the Tasanh Health Center. Subjects will only be treated for injuries that are directly caused by the research study. They will also be compensated for transportation to and from the hospital or clinic.

# Assessment of Outcome Measures

## Specification of the Appropriate Outcome Measures

Outcome measures for the clinical study is based on the criteria set forth by WHO in the “Assessment and Monitoring of Antimalarial Drug Efficacy for the Treatment of Uncomplicated Falciparum Malaria” for low to moderate transmission areas (WHO, 2003).

### Primary Outcome Measures (Clinical Outcomes)

PCR-corrected clinical outcome will be assessed for Day 28 and 42.

Classification of clinical outcomes:

- **Adequate Clinical and Parasitological Response (ACPR)**

• Absence of parasitemia on Day 28 and 42 irrespective of temperature without previously meeting any of the criteria of Early Treatment Failure or Late Clinical Failure or Late Parasitological Failure.

- **Early Treatment Failure (ETF)**

• Development of danger signs (e.g. impaired consciousness, convulsions, respiratory distress) or severe malaria on Day 1, Day 2 or Day 3, in the presence of parasitemia

• Parasitemia on Day 2 higher than Day 0 count irrespective of temperature

• Parasitemia on Day 3 with temperature ≥ 37.5 °C

• Parasitemia on Day 3 ≥ 25 % of count on Day 0.

- **Late Treatment Failure (LTF)**
  - Late Clinical Failure (LCF)

• Development of danger signs or severe malaria after Day 3 in the presence of parasitemia, without previously meeting any of the criteria of Early Treatment Failure

• Presence of parasitemia and temperature ≥ 37.5 °C (or history of fever) on any day from Day 4 to Day 28/42 without previously meeting any of the criteria of Early Treatment Failure

- - Late Parasitological Failure (LPF)

• Presence of parasitemia on any day from Day 7 to Day 42 and temperature < 37.5 °C, without previously meeting any of the criteria of Early Treatment Failure or Late Clinical Failure

### Primary Outcome Measures (Laboratory Outcomes)

Classification of laboratory outcomes.

- **Inhibitory Concentrations (ICs)**

• Inhibitory concentrations will be calculated by nonlinear regression analysis. The outcome is a continuous variable.

**• Complete Blood Counts**

• Hemoglobin and white blood cell counts with differential to determine the absolute neutrophil count will be compared at designated time points between dosing arms.

### Primary Outcome Measures (Safety and Tolerability)

• Occurrence of treatment emergent adverse events both in total and in comparison of dosing arms will be determined.

### Secondary Outcome Measures

The secondary clinical endpoints are time until full clearance of parasites (parasite clearance time, PCT), gametocytes (gametocyte clearance time, GCT), and to disappearance of fever (fever clearance time, FCT: the time from start of treatment until the temperature drops to below 37.5 °C and remains below this temperature during the next 48 hours). The event time for clearance times is defined as the time until the first in a series of negative tests occurs. PCT, GCT, and FCT are continuous variables. The proportion of subjects still parasitemic on Day 3 (72 hours) will be calculated and compared with the other study sites.

# Safety assessment and reporting

An independent Safety Monitoring Committee (SMC) will be established. The prime responsibility of the SMC will be to assess and ensure the safety of patients in the high-dose cohort in comparison to the other two dosing arms,; its charter is given in Appendix C.

Adverse event assessment will be done throughout the study duration of 42 days. Treatment-emergent AEs for each patient will be documented daily on a standard form during the 7 days of in-patient artesunate treatment in addition to other routine AE assessments. These forms will be made available to the SMC for monitoring and review.

## Definition of Adverse Event (AE)

An AE is defined as any untoward medical occurrence in a subject undergoing a study related procedure and believed reasonably to be caused by that study related procedure.

## Definition of Serious Adverse Event (SAE)

An SAE is any untoward medical occurrence regardless of cause or relationship to study drug that:

- Results in death.
- Is life-threatening. Any adverse experience that places the subject, in the view of the investigator, at immediate risk of death from the reaction as it occurred (i.e., it does not include a reaction that, had it occurred in a more serious form, might have caused death).
- Requires in-patient hospitalization (excluding the hospitalization required by the study) or prolongation of existing hospitalization.
- Results in persistent or significant disability or incapacity.
- Is a congenital anomaly/birth defect.
- An event that required intervention to prevent permanent impairment or damage.
- Important medical events that do not result in death, are not life-threatening, or do not require hospitalization may be considered serious adverse events when, based upon appropriate medical judgment, they might jeopardize the subject and might require medical or surgical intervention to prevent one of the outcomes listed above.

## Safety Reporting Procedures

All observed or volunteered adverse events regardless of treatment group or suspected causal relationship to study drug will be recorded on the adverse event pages of the case report form. Events involving adverse drug reactions, illnesses with onset during the study, or exacerbations of pre-existing illnesses other than malaria should be recorded. Exacerbation of pre-existing illness, including the disease under study, is defined as a manifestation (sign or symptom) of the illness that indicates a significant increase in the severity of the illness as compared to the severity noted at the start of the trial. It may include worsening or increase in severity of signs or symptoms of the illness, increase in frequency of signs and symptoms of an intermittent illness, or the appearance of a new manifestation/complication. Exacerbation of a pre-existing illness should be considered when a patient/subject requires new or additional concomitant drug or non-drug therapy for the treatment of that illness during the trial. Lack of or insufficient clinical response, benefit, efficacy, therapeutic effect, or pharmacologic action should not be recorded as an adverse event. The investigator must make the distinction between exacerbation of pre-existing illness and lack of therapeutic efficacy. In addition, clinically significant changes in physical examination findings and abnormal objective test findings (e.g., laboratory) should also be recorded as adverse events. The criteria for determining whether an abnormal objective test finding should be reported as an adverse event are as follows:

- test result is associated with accompanying symptoms, and/or
- test result requires additional diagnostic testing or medical/surgical intervention, and/or
- test result leads to a change in study dosing or discontinuation from the study, significant additional concomitant drug treatment or other therapy, and/or
- test result leads to any of the outcomes included in the definition of a serious adverse event, and/or
- test result is considered to be an adverse event by the investigator

Merely repeating an abnormal test, in the absence of any of the above conditions, does not meet conditions for reporting as an adverse event.

Any abnormal test result that is determined to be an error does not require reporting as an adverse event, even if it did meet one of the above conditions except for condition #4.

**9.3.1.** **Serious Adverse Event Detection and Reporting**

All serious adverse events will be recorded on the appropriate serious adverse event case report form, followed through resolution by a study physician, and reviewed by a study physician.

All unanticipated problems involving risk to subjects or others, serious adverse events related to participation in the study and subject deaths related to participation in the study should be promptly reported by phone, fax, or email to the following:

Division of Human Subjects Protection (DHSP)

Walter Reed Army Institute of Research (WRAIR)

(301) 319-9940 (Tel)

(301) 319 -9163 (Fax)

dhsp@wrairdhsp.com

U.S. Army Medical Research and Materiel Command

Office of Research Protections, Human Research Protection Office

(301) 619-2165 (Tel)

(301) 619-7803 (Fax)

hsrrb@det.amedd.army.mil

A complete written report will follow the initial notification. In addition to the methods above, the complete report will be sent to the Walter Reed Army Institute of Research Division of Human Subjects Protection, 503 Robert Grant Avenue, Silver Spring, MD 20910 and to the U.S. Army Medical Research and Materiel Command, ATTN: MCMR-ZB-PH, 504 Scott Street, Fort Detrick, Maryland 21702-5012, and also to the WHO Ethical Review Committee, Geneva, Switzerland, and to the National Ethics Committee for Health Research, Cambodia.

All serious adverse events regardless of treatment group or suspected relationship to study drug must be reported within 24 hours of the event by telephone to the medical monitor

Dr *Koy Lenin*

*Battambang Referral Hospital*

*Emmergency and ICU unit*

*Dongkorteap village, Tuol Ta Ek Commun,*

*Battambang district, Battambang province*

*Tel: 053 730 100; Fax: 053 953 223*

who will review and report all serious and unexpected adverse events to higher headquarters within the U.S. Army Medical Research and Materiel Command (USAMRMC).

The medical monitor is required to review all unanticipated problems involving risk to subjects or others, serious adverse events and all subject deaths associated with the protocol and provide an unbiased written report of the event. At a minimum, the medical monitor must comment on the outcomes of the event or problem and in case of a serious adverse event or death, comment on the relationship to participation in the study. The medical monitor must also indicate whether he/she concurs with the details of the report provided by the principal investigator. Reports for events determined by either the investigator or medical monitor to be possibly or definitely related to participation and reports of events resulting in death must be promptly forwarded to the WRAIR IRB, the USAMRMC ORP HRPO and the Ethical Review Committee of WHO.

The study clinician will complete a Serious Adverse Event Form within the following timelines:

- All deaths, whether associated or not associated, will be recorded on the Serious Event Form and sent by fax within 24 hours of site awareness of the death.
- Serious adverse events other than death, regardless of relationship, will be reported via fax by the site within 72 hours of becoming aware of the event.

All SAEs will be followed until satisfactory resolution or until the Principal Investigator or Sub-investigator deems the event to be chronic or the patient to be stable.

ICH GCP 6, Section 4.11 require that an investigator notifies the sponsor, regulatory authority(ies) and the local IRB immediately of any serious adverse event, deaths, or life-threatening problems that occur in the study. The SMC will also be informed.

### 9.3.2. Type and Duration of the Follow-up of Subjects After Adverse Events

Adverse events will be followed by the study team until resolved or considered stable. If by Day 42 the AE has not resolved the patient will be followed after the normal observation period. The investigators will collect pregnancy information on any female subject, who becomes pregnant while participating in this study. The investigators will record pregnancy information on the appropriate form. The subject will also be followed to determine the outcome of the pregnancy. Generally, follow-up will be no longer than 6 to 8 weeks following the estimated delivery date. Any premature termination of the pregnancy will be reported. While pregnancy itself is not considered to be an AE or SAE, any pregnancy complication or elective termination of a pregnancy for medical reasons will be recorded as an AE or SAE. A spontaneous abortion is always considered to be an SAE and will be reported as such.

## Halting Rules

### Individual Halting Rules

Individual study subjects will be withdrawn from their randomized dosing arm and receive no further artesunate doses if any of the following values or criteria (representing possible artesunate toxicity) are met:

• **Hematologic**

**•** hemoglobin <7 mg/dL (WHO criteria cut-off for severe anemia in severe malaria**)**

• decrease in hemoglobin from baseline of > 3.6 mg/dL (two standard deviations from the Day 0 hemoglobin from uncomplicated falciparum patients enrolled into the previously completed DRT WRAIR Protocol #1263). This may be an over-cautious cut-off and may need to be modified since the Day 0 CBC value is often artificially elevated due to hemoconcentration secondary to dehydration which is often observed in acute malaria patients, and also a drop in blood count is an expected occurrence in patients recovering from malaria especially those with higher baseline parasitemias.

 • absolute neutrophil count < 1 x 10^9^ /L

**• Neurologic**

- obtundation,
- new or worsening ataxia,
- > 1 seizure

**• Gastrointestinal or Genitourinary**

- visibly bloody stools, not due to another etiology
- visible hematuria or hemoglobinuria, not due to another etiology

### Cohort Halting Rules

• more than 30% failures in one of the arms detected by Day 28 in a preliminary efficacy analysis which will be performed after enrollment of 50% of the subjects into this arm.

• >4 individual halts in the same arm for the same adverse event

• >1 SAEs in any enrolment arm

• This study or part of this study may be terminated or suspended at any time at the discretion of the PI or SMC.

If a study or part of a study is terminated or suspended for any reason, the investigators will promptly inform the institutions and the regulatory authorities, which will be informed and provided the reason(s) for the termination or suspension by the investigator/institution/SMC. The patients should be notified, with IRB guidance.

### Enrolment Pause in Arm 3 for Safety Monitoring

New subject enrolment will be temporarily suspended after the first 5 subjects have been enrolled into the higher dose (Arm 3, 6 mg/Kg/day) regimen, pending a safety review by the SMC

# Clinical Monitoring Structure

AFRIMS will monitor this study. Regular monitoring visits will be performed by AFRIMS staff trained in GCP and study monitoring under supervision of Mrs. Mali Ittiverakul RN. Training in protocol and procedures will be provided prior to study initiation. AFRIMS will provide pre-study visits, regular monitoring visits, and a post-study visit. Reports will be generated after each visit and sent to PIs and QA Officer at AFRIMS, Thailand.

Medical Monitor (as required by AR 40-7) will be a licensed physician based at the Battambang Hospital, Battambang, Cambodia. His duties include monitoring the study subjects from a medical perspective, reviewing and reporting all serious and unexpected adverse events, ensuring medical care for any such events should they occur, and reporting serious events to higher headquarters. He will perform on-site visits approximately every 3 months and as needed.

## Site Monitoring Plan

The site will be monitored regularly to ensure the human subject protection, study procedures, laboratory, and data collection processes are of high quality and meet GCP/ICH and regulatory guidelines. Monitoring will consist of a pre-study visit, regular monitoring visits, and a post-study visit. A minimum of 10% of all CRFs will be reviewed by the monitors.

## Protocol Deviations

Protocol deviations will be reported to the local IRB and the WRAIR IRB and Human Research Protection Office (HRPO) as part of the annual report. Any deviations that fit the category of “unanticipated problems involving risks to volunteers or others” should be promptly reported. Any deviation to the protocol that may have an effect on the safety or rights of the subject or the integrity of the study must be reported to the WRAIR IRB and to the USAMRMC ORP HRPO as soon as the deviation is identified.

# Statistical Considerations

## Study Outcome Measures and Analysis

The primary outcome for the clinical part of the study will be cure rates in arms 1 and 3. Cure rates will be analyzed separately for Day 28 and 42.

The primary outcome of this study is a nominal variable: the treatment will be characterized as successful or unsuccessful/resistant. Cure rates will be summarized along with 95% confidence intervals (exact based on binomial distribution). The resulting categorical frequency data between arm 1 and 3 will be compared in Chi^2^-tests.

The secondary endpoints of this study are continuous variables. Overall group differences between secondary endpoints of parasite clearance time (PCT), gametocyte clearance time (GCT), and fever clearance time (FCT) will be compared using the Mann-Whitney U test. In vitro data will be analyzed by nonlinear regression models. The alpha level for tests used to compare differences between treatment groups will be 5%.

## Sample Size Considerations

Enrollment target for arms 1 and 3 will be 60 evaluable subjects (those completing study endpoints). Arm 2 will be used as a comparator for the 4 mg/kg study cohort tested in the study conducted in 2006. Subjects will be enrolled in the 3 groups at a ratio of 2:1:2. The dropout rate is expected to be approximately 25%. Any dropouts (e.g. withdrawals, lost-to-follow-ups etc.) will be replaced in the same arm. Therefore 190 persons may be enrolled to achieve 150 evaluable subjects. In case the enrollment target of 150 evaluable subjects cannot be met within the approved study duration an application for extension of the study duration will be filed.

Arm 2 serves as a control and will serve as a bridge to the study performed in 2006/2007. The sample size in this arm is lower as results from 60 evaluable patients are available from the earlier study conducted at the same site.

The sample size for detecting a significant difference between the 2 study arms by uncorrected Chi^2^-test is calculated based on an expected cure rate (i.e. early and late treatment failures) of 80% in arm 1 and 95 % in arm 3:

α 0.1

1 – β 0.8

p_0_ 0.8

p_1_ 0.95

Calculated sample size per arm 59

With 60 patients in arm 1 and 60 patients in arm 3 there will be a >80% chance of detecting a significant difference by uncorrected Chi^2^-test at a two sided 0.1 significance level. This assumes that the response rate in arm 1 is 0.8 and the response rate in arm 3 is 0.95.

Figure 1: Case sample size relative to power for α = 0.1 (Dupont and Plummer 1997)


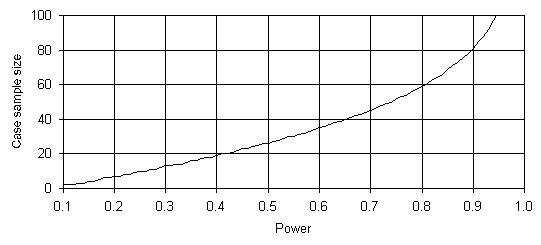


## Participant Enrollment and Follow-Up

190 persons may be enrolled to achieve 150 evaluable subjects. Study participants will be randomly assigned to one of three treatment groups and followed for 42 days.

# Access to Source Data/Documents

The study site will maintain appropriate medical and research records for this trial until completion of the study, in compliance with Section 4.9 of ICH E6 GCP, and regulatory and institutional requirements for the protection of confidentiality of subjects. The database will be kept at AFRIMS. Data will be shared with the investigators in Cambodia. The site will permit authorized representatives of AFRIMS and regulatory agencies, the MRMC, Human Research Protection Office (HRPO), and WRAIR IRB to examine (and when required by applicable law, to copy) clinical records for the purposes of quality assurance reviews, audits and evaluation of the study safety and progress.

CRF data will be entered into an electronic database. Data entry will start while the study is being conducted. Single data entry by trained AFRIMS staff will be used and all data will be crosschecked. The database will be maintained at AFRIMS for up to 25 years. The database will only contain information collected through the CRFs and laboratory data and will be password protected to limit access to the data. No reference will be made in the database that will allow for a direct identification of the subjects. The patient enrollment log containing subject-specific information will be kept separately in a secure place at AFRIMS and will not be accessible for data analysis. The QC Unit at the Department of Immunology and Medicine will hold the link between the unique ID numbers in the database and personal identifying information. After data entry source documents will be stored in a safe place at the National Center for Parasitology, Entomology and Malaria Control in Phnom Penh under supervision of the PI. Coded data will be shared with the Cambodian investigators.

This study will not involve the collection of data on sensitive matters such as sexual behavior or criminal activities. No HIV or human genetic testing will be pefomed on any samples collected during this study. This protocol does not involve audio or videotaping of research subjects. All subject records and CRFs will be carefully designed to limit the personal information to be acquired to that, which is essential. Data that could reveal a subject’s identity will be stored in files accessible only to authorized staff. As early as feasible, the data will be coded to remove identifying information.

Source data are all information, original records of clinical findings, observations, or other activities in a study necessary for the reconstruction and evaluation of the trial. Examples of these original documents and data records include, but are not limited to, hospital records, clinical and office charts, laboratory notes, memoranda, or evaluation checklists, pharmacy dispensing records, recorded data from automated instruments, copies or transcriptions certified after verification as being accurate and complete, microfiches, photographic negatives, microfilm or magnetic media, x-rays, and subject files and records kept at the pharmacy, at the laboratories, and medico-technical departments involved in the clinical study. Source data will be maintained at the National Center for Parasitology, Entomology and Malaria Control, Phnom Penh, under supervision of the principal investigator for at least 2 years after formal completion of the trial.

# Quality Control and Quality Assurance

Quality control (QC) will be performed by AFRIMS. If not otherwise specified the procedures will follow AFRIMS SOPs/SSPs for quality management. Data will be evaluated for compliance with protocol and accuracy in relation to source documents. The study will be conducted in accordance with procedures identified in the protocol.

Protocol compliance, ethical standards, regulatory compliance, data quality and proper storage and handling of samples will be assured by quality control and monitoring.

A QC officer and nurse with GCP and CRO training from AFRIMS will be assigned to perform QC. AFRIMS has monitored several regulated clinical trials. He/she will have ready access to the study patients and their records. He/she will help ensure compliance with GCP requirements and resolve problems with CRF completion. Furthermore, he/she will file copies of all monitoring visits at AFRIMS.

# Ethics/Protection of Human Subjects

## Declaration of Helsinki

The investigator will ensure that this study is conducted in full conformity with the current revision of the Declaration of Helsinki (Fifth revision October 2000), or with the International Conference for Harmonization Good Clinical Practice (ICH-GCP) regulations and guidelines, whichever affords the greater protection to the subject.

## Institutional Review Board

The protocol and informed consent documents will be provided for the review and approval.

The protocol will require scientific review and approval by the committee at AFRIMS. The protocol will undergo ethical review and require approval by the U.S. Army Medical Research and Materiel Command's Human Research Protection Office (HRPO), WRAIR IRB, the National Ethics Committee for Health Research Health IRB# 1 (FWA# 00010451, IRB # 00003143), Phnom Penh, Cambodia and the Ethical Review Committee of the World Health Organization, Geneva, Switzerland (FWA#00007093, IRB#00003055).

Any modifications that could potentially increase risk to subjects must be submitted to WRAIR DHSP and the USAMRMC ORP HRPO for approval prior to implementation. All amendments must be submitted for review and approval by all institutional review boards. Amendments will be submitted to all institutional review boards at the time of the needed change.

If modifications are required, they will be submitted in writing to the USAMRMC Office of Research Protections, the WRAIR Division of Human Subjects Protection and the National Ethics Committee Health Research, Phnom Penh, Cambodia and the Ethical Review Committee of WHO. A revised consent form will accompany any request for modification that changes any issue addressed in the currently approved consent form. All amendments to the protocol and informed consent form, which require regulatory and/or IRB/IEC approval/favorable opinion, must be reviewed and approved by IRB/IEC and/or local authorities before being implemented. Amendments should not be implemented until all necessary approvals have been obtained, except where necessary to eliminate an immediate hazard(s) to study subjects. All amendments to the protocol must be submitted to the Human Research Protection Office (HRPO) and the WRAIR IRB after review and approval at the local level, but before implementation. This amendment will receive either full Human Research Protection Office (HRPO) review and WRAIR IRB review or expedited review by the Acting Chair, as appropriate. The amendment may be implemented after WRAIR Commander final authorization.

A copy of the continuing review report and the local IRB approval notification will be submitted to the WRAIR IRB. After the continuing review report is approved by the WRAIR IRB, the approved CRR, as well as the local and WHO IRB approval notificaiton will be submitted to the USAMRMC ORP HRPO. The final study report and local IRB approval notification will be submitted to the WRAIR IRB. Once approved, a copy of the final study report and local IRB approval notification will be submitted to the USAMRMC ORP HRPO.

The knowledge of any pending compliance inspection/visit by the FDA, OHRP, or other government agency concerning clinical investigation or research, the issuance of Inspection Reports, FDA Form 483, warning letters or actions taken by any Regulatory Agencies including legal or medical actions and any instances of serious or continuing noncompliance with the regulations or requirements will be reported immediately to WRAIR DHSP and the USAMRMC ORP HRPO.

## Informed Consent

Freely given informed consent will be obtained from every subject prior to study participation. Informed consent must take place before any study specific procedure, prior to the initiation of non-routine study-related tests, and prior to administration of study drug. Signed and dated, informed consent will be obtained from each subject in accordance with GCPs and with local regulatory and legal requirements. The completed informed consent form must be retained by the investigator as part of the study records and a copy will be provided to study subjects. The investigators, or a person designated by the investigators, will fully inform the subject of all pertinent aspects of the trial including the written information given approval/favorable opinion by the IRB/IEC. Neither the investigator, nor the trial staff, will coerce or unduly influence a subject to participate or to continue to participate in the study.

In obtaining and documenting informed consent, the investigators will comply with the applicable regulatory requirement(s), and will adhere to GCP and to the ethical principles that have their origin in the Declaration of Helsinki (5th revision, 2000). Prior to the beginning of the trial, the investigators will have the IRB/IEC's written approval/favorable opinion of the written informed consent form and any other written information to be provided to subjects.

The written informed consent form and any other written information to be provided to subjects will be revised whenever important new information becomes available that may be relevant to the subject’s consent. Any revised written informed consent form, and written information will receive the IRB/IEC's approval/favorable opinion in advance of use. The subject will be informed in a timely manner if new information becomes available that may be relevant to the subject’s willingness to continue participation in the trial. The communication of this information will be documented.

### Informed Consent Process

Informed consent is a process that is initiated prior to the individual’s agreeing to participate in the study and continuing throughout the individual’s study participation. Extensive discussion of risks and possible benefits of participation in this study will be provided to the subjects and their families. Consent forms describing in detail the study procedures and risks are given to the subject and written documentation of informed consent is required prior to enrolling in the study. Consent forms will be IRB approved and the subject will be asked to read and review the document. If the patient cannot read the content of the consent form will be read and explained to him by study personnel. Upon reviewing the document, the study personnel will explain the research study to the subject and answer any questions that may arise. The subjects will sign the informed consent document prior to being enrolled in the study. One witness will sign and date the consent form in the presence of the participant attesting that the requirements for informed consent have been satisfied and that consent is voluntary and freely given by the subject without any element of force, fraud, deceit, duress, coercion, or undue influence. The subjects should have the opportunity to discuss the study with their surrogates or think about it prior to agreeing to participate. The subjects may withdraw consent at any time throughout the course of the study. Following ICH guidelines a signed copy of the informed consent document will be given to the subjects for their records. The rights and welfare of the subjects will be protected by emphasizing to them that the quality of their medical care will not be adversely affected if they decline to participate in this study.

## Subject Confidentiality

All personal study subject data collected and processed for the purposes of this study should be managed by the investigators and his/her staff with adequate precautions to ensure the confidentiality of those data, and in accordance with applicable national and/or local laws and regulations on personal data protection.

Monitors, auditors and other authorized agents, the United States Army Medical Research and Materiel Command, and the ethics committees approving this research will be granted direct access to the study subjects’ original medical records for verification of clinical trial procedures and/or data, without violating the confidentiality of the subjects, to the extent permitted by the law and regulations. In any presentations of the results of this study at meetings or in publications, the subjects’ identity will remain confidential.

Subject names will also be added to the Volunteer Registry Database as required by the US Army Medical Research and Materiel Command (USAMRMC) whenever human volunteers are used in research studies. This database is maintained only for patient safety and will be kept in a secure location at AFRIMS in Bangkok. It is the policy of USAMRMC that data sheets are to be completed on all volunteers participating in research for entry into the U.S. Army Medical Research and Materiel Command Volunteer Registry Database. The information to be entered into this confidential database includes name, address, social security number (if applicable), study name, and dates. The information will be stored at the AFRIMS for a minimum of 75 years.

## Future Use of Stored Specimens

After the study is completed residual specimens will be stored and only used for purposes mentioned in the consent form (particularly drug sensitivity assays on cryopreserved parasite isolates). During the trial specimens will be stored in a safe place at the research laboratory at the study site, which is only accessible to study staff. They will be regularly transferred to AFRIMS where they may be stored for up to 20 years. No one will have access to the specimens without the explicit permission of the PIs. Samples may only be used for purposes not mentioned in the protocol after approval of the IRB of record, WRAIR IRB and HRPO.

**14.6 COMPENSATION**

Compensation will be provided throughout the study, and subjects will receive partial compensation if they leave the study before completion. The estimated compensation for completion of the trial will be approximately 260,000 Riel (current exchange rate, 1USD: approximately 4,000 Riel). This compensation takes into consideration lost earnings, food, transportation and discomfort from phlebotomy. During the first week study subjects will be provided a daily compensation of 16,000 Riel/day; For the visits on Day 14, 21, 28 and 35 patients will be provided 20,000 Riel plus reimbursement of actual transportation expense for each visit and on Day 42 they will receive another 20,000 Riel plus 4,000 Riel for every day of their stay at the hospital or study facility in the first week.

# Literature References

Adjuik M, Agnamey P, Babiker A et al. Amodiaquine-artesunate versus amodiaquine for uncomplicated Plasmodium falciparum malaria in African children: a randomised, multicentre trial. Lancet 2002 Apr 20;359(9315):1365-72.

Breman JG. The ears of the hippopotamus: manifestations, determinants, and estimates of the malaria burden. Am J Trop Med Hyg. 2001 Jan-Feb;64(1-2 Suppl):1-11.

Brewer TG, Peggins JO, Grate SJ et al. Neurotoxicity in animals due to arteether and artemether. Trans R Soc Trop Med Hyg 1994 Jun;88 Suppl 1:S33-6.

Bunnag D, Viravan C, Looareesuwan S et al. Clinical trial of artesunate and artemether on multidrug resistant falciparum malaria in Thailand: a preliminary report. SE Asian J Trop Med Publ Health 1991; 22:380-85.

Bustos MD, Gay F, Diquet B. In-vitro tests on Philippine isolates of Plasmodium falciparum against four standard antimalarials and four qinghaosu derivatives. Bull World Health Organ. 1994;72(5):729-35.

Campos S, de la Cerda P, Rivera A. Fatal artesunate toxicity in a child. Journal of Pediatric Infectious Diseases. 2008:3:1.

Central Intelligence Agency, World fact book/Cambodia, 28 Feb, 2008. website: https://www.cia.gov/library/publications/the-world-factbook/geos/cb.html#top retrieved on 04 March 4, 2008.

Denis MB, Tsuyuoka R, Poravuth Y, Narann TS, Seila S, Lim C, Incardona S, Lim P, Sem R, Socheat D, Christophel EM, Ringwald P. Surveillance of the efficacy of artesunate and mefloquine combination for the treatment of uncomplicated falciparum malaria in Cambodia. Trop Med Int Health. 2006 Sep;11(9):1360-6.

Denis MB, Tsuyuoka R, Lim P, Lindegardh N, Yi P, Top SN, Socheat D, Fandeur T, Annerberg A, Christophel EM, Ringwald P. Efficacy of artemether-lumefantrine for the treatment of uncomplicated falciparum malaria in northwest Cambodia. Trop Med Int Health. 2006 Dec;11(12):1800-7.

Doherty JF, Sadiq AD, Bayo L, Alloueche A et al. A randomized safety and tolerability trial of artesunate plus sulfadoxine--pyrimethamine versus sulfadoxine-pyrimethamine alone for the treatment of uncomplicated malaria in Gambian children. Trans R Soc Trop Med Hyg. 1999 Sep-Oct;93(5):543-6.

Dondorp A, Nosten F, Stepniewska K, Day N, White N; South East Asian Quinine Artesunate Malaria Trial (SEAQUAMAT) group. Artesunate versus quinine for treatment of severe falciparum malaria: a randomised trial. Lancet. 2005 Aug 27-Sep 2;366(9487):717-25.

Dupont WD and Plummer WD: PS power and sample size program. Controlled Clin Trials,1997;18:274.

Genovese RF, Newman DB, Brewer TG. Behavioral and neural toxicity of the artemisinin antimalarial, arteether, but not artesunate and artelinate, in rats. Pharmacol Biochem Behav 2000 Sep;67(1):37-44.

Gogtay NJ, Kadam VS, Karnad DR, Kanbur A, Kamtekar KD, Kshirsagar NA. Probable resistance to parenteral artemether in Plasmodium falciparum: case reports from Mumbai (Bombay), India. Ann Trop Med Parasitol. 2000 Jul;94(5):519-20.

Hien TT, White NJ. Qinghaosu. Lancet 1993:341:603-8

Investigator Brochure for IV Artesunate for Severe Malaria, IND 64,769, ver 2.0

Karbwang J, Na-Bangchang K. Clinical pharmakology of artemisinin compounds. In: Karbwang J, Wernsdorfer WH, eds. Clinical pharmakology of antimalarials. Bangkok: Faculty of Tropical Medicine, 1993: 263-304.

Li GQ, Guo XB, Fu LC et al. Clinical trials of artemisinin and its derivatives in the treatment of malaria in China. Trans Roy Soc Trop Med Hyg 1994; 88: suppl. 1, 5-6.

Li GQ, Fu YX and Bian WX. Comparison on treatment of falciparum malaria with different courses of artesunate tablet (Chinese). Chung Kuo Chung Hsi I Chieh Ho Tsa Chih 1997; 17: 143-4.

Looareesuwan S. Overveiw of clinical studies on artemisinin derivatives in Thailand. Trans Roy Soc Trop Med Hyg 1994; 88: Suppl. 1, 88-90.

Looaresuwan S, Vanijanonta S, Viravan C, et al. Randomised trial of mefloquine-tetracycline and quinine-tetracycline for acute complicated falciparum malaria. Acta Tropica 1994; 57:47-53.

Looareesuwan S, Wilairatana P, Vanijanonta S, Pitisuttithum P, Ratanapong Y, Andrial M. Monotherapy with sodium artesunate for uncomplicated falciparum malaria in Thailand: a comparison of 5- and 7-day regimens. Acta Trop 1997 Sep 30;67(3):197-205.

Luxemburger C, Brockman A, Silamut K, Nosten F, van Vugt M, Gimenez F, Chongsuphajaisiddhi T, White NJ. Two patients with falciparum malaria and poor in vivo responses to artesunate. Trans R Soc Trop Med Hyg. 1998 Nov-Dec;92(6):668-9.

McGready R, Cho T, Cho JJ et al. Artemisinin derivatives in the treatment of falciparum malaria in pregnancy. Trans R Soc Trop Med Hyg 1998 Jul-Aug;92(4):430-3.

McGready R, Cho T, Keo NK et al. Artemisinin antimalarials in pregnancy: a prospective treatment study of 539 episodes of multidrug-resistant Plasmodium falciparum.Clin Infect Dis. 2001 Dec 15;33(12):2009-16.

Meshnick SR. Artemisinin: mechanisms of action, resistance and toxicity. Int J Parasitol. 2002 Dec 4;32(13):1655-60.

Noedl H. Artemisinin resistance: how can we find it? Trends Parasitol. 2005 Jul 18

Noedl H, Attlmayr B, Wernsdorfer WH, Kollaritsch H, Miller RS. A histidine-rich protein 2-based malaria drug sensitivity assay for field use. Am J Trop Med Hyg. 2004 Dec;71(6):711-4.

Noedl H, Wernsdorfer WH, Miller RS, Wongsrichanalai C. Histidine-rich protein II: a novel approach to malaria drug sensitivity testing. Antimicrob Agents Chemother. 2002 Jun;46(6):1658-64.

Noedl H, Wernsdorfer WH, Krudsood S, Wilairatana P, Viriyavejakul P, Kollaritsch H, Wiedermann G, Looareesuwan S. *In vivo-in vitro* model for the assessment of clinically relevant antimalarial cross-resistance. Am J Trop Med Hyg. 2001 Dec;65(6):696-9.

Nontprasert A, Pukrittayakamee S, Dondorp AM, Clemens R, Looareesuwan S, White NJ. Neuropathologic toxicity of artemisinin derivatives in a mouse model. Am J Trop Med Hyg 2002 Oct;67(4):423-9

Pickard AL, Wongsrichanalai C, Purfield A, Kamwendo D, Emery K, Zalewski C, Kawamoto F, Miller RS, Meshnick SR. Resistance to antimalarials in Southeast Asia and genetic polymorphisms in pfmdr1. Antimicrob Agents Chemother. 2003 Aug;47(8):2418-23.

Price R, Van Vugt M, Phaipun et al. Adverse effects in patients with acute falciparum malaria treated with artemesinin derivatives. Am J Trop Med Hyg 1999; 60:547-55.

Purfield A, Nelson A, Laoboonchai A, Congpuong K, McDaniel P, Miller RS, Welch K, Wongsrichanalai C, Meshnick SR. A new method for detection of pfmdr1 mutations in Plasmodium falciparum DNA using real-time PCR. Malar J. 2004 May 7;3:9.

Ringwald P, Bickii J, Basco LK. In vitro activity of dihydroartemisinin against clinical isolates of Plasmodium falciparum in Yaounde, Cameroon. Am J Trop Med Hyg 1999 Aug;61(2):187-92.

Sahr F, Willoughby VR, Gbakima AA, Bockarie MJ. Apparent drug failure following artesunate treatment of Plasmodium falciparum malaria in Freetown, Sierra Leone: four case reports. Ann Trop Med Parasitol. 2001 Jul;95(5):445-9.

Teja-Isavadharm P, Watt G, Eamsila C et al. Comparative pharmacokinetics-effect kinetics of oral artesunate in healthy volunteers and patients with uncomplicated malaria. Am J Trop Med Hyg. 2001 Dec;65(6):717-21.

Thimasarn K, Jatapadma S, Vijaykadga S, Sirichaisinthop J, Wongsrichanalai C. Epidemiology of Malaria in Thailand. J Travel Med. 1995 Jun 1;2(2):59-65.

Vijaykadga S, Rojanawatsirivej C, Cholpol S, Phoungmanee D, Nakavej A, Wongsrichanalai C. In vivo sensitivity monitoring of mefloquine monotherapy and artesunate-mefloquine combinations for the treatment of uncomplicated falciparum malaria in Thailand in 2003. Trop Med Int Health. 2006 Feb;11(2):211-9.

von Seidlein L, Milligan P, Pinder M et al. Efficacy of artesunate plus pyrimethamine-sulphadoxine for uncomplicated malaria in Gambian children: a double-blind, randomised, controlled trial. Lancet 2000 Jan 29;355(9201):352-7.

Wongsrichanalai C, Wimonwattrawatee T, Sookto P et al. In vitro sensitivity of Plasmodium falciparum to artesunate in Thailand. Bull WHO 1999;77:392-8.

Wongsrichanalai C, Wimonwattrawatee T, Sookto P et al. In vitro sensitivity of Plasmodium falciparum to artesunate in Thailand. Bull WHO 1999;77:392-8.

Wongsrichanalai C, Pickard AL, Wernsdorfer WH, Meshnick SR. Epidemiology of drug-resistant malaria. Lancet Infect Dis. 2002 Apr;2(4):209-18.

Wongsrichanalai C, Sirichaisinthop J, Karwacki JJ, Congpuong K, Miller RS, Pang L, Thimasarn K. Drug resistant malaria on the Thai-Myanmar and Thai-Cambodian borders. Southeast Asian J Trop Med Public Health. 2001 Mar;32(1):41-9.

Wongsrichanalai C, Wimonwattrawatee T, Sookto P, Laoboonchai A, Heppner DG, Kyle DE, Wernsdorfer WH. In vitro sensitivity of Plasmodium falciparum to artesunate in Thailand. Bull World Health Organ. 1999;77(5):392-8.

Wongsrichanalai C, Webster HK, Wimonwattrawatee T, Sookto P, Chuanak N, Thimasarn K, Wernsdorfer WH. Emergence of multidrug-resistant Plasmodium falciparum in Thailand: in vitro tracking. Am J Trop Med Hyg. 1992 Jul;47(1):112-6.

World Health Organization. Severe falciparum malaria. Trans R Soc Trop Med Hyg 2000; 94,Suppl 1:1-90.

World Health Organization. Assessment and Monitoring of Antimalarial Drug Efficacy for the Treatment of Uncomplicated Falciparum Malaria. WHO/HTM/RBM/2003.50. Geneva, 2003.

World Health Organization. World Malaria report 2005. Last accessed 24 Feb 2006. http://rbm.who.int/wmr2005/html/a1_1.htm

**Appendix A: Schedule of Procedures/Evaluations**

| **Day of Study** | **0** | **1** | **2** | **3** | **4** | **5** | **6** | **14, 21, 28 35 and 42** | **Failure** |
| --- | --- | --- | --- | --- | --- | --- | --- | --- | --- |
| Informed Consent | X |  |  |  |  |  |  |  |  |
| Eligibility Criteria | X |  |  |  |  |  |  |  |  |
| Demographics | X |  |  |  |  |  |  |  |  |
| Medical History | X |  |  |  |  |  |  |  |  |
| Physical Exam* | X | X | X | X | X | X | X | X | X |
| Prior/Concomitant Medication * | X | X | X | X | X | X | X | X | X |
| Vital Signs* | X | X | X | X | X | X | X | X | X |
| Adverse Events* | X | X | X | X | X | X | X | X | X |
| Clinical Signs & Symptoms* | X | X | X | X | X | X | X | X | X |
| Microscopy:  *Blood Smear********* | X | X | X | X | X | X | X | X | X |
| Clinical Laboratory:  *Urine collection* (all) and pregnancy test (females) | X |  |  |  |  |  |  |  |  |
| Clinical Laboratory:  *Hematology,*  *ALT*  *Glucose**** | X |  |  | X |  |  | X | X” | X |
| PK series | X^ |  |  |  |  |  | X^^ |  |  |
| Bioassay | X |  |  |  |  |  |  |  | X |
| PCR# | X | X | X | X | X | X | X | X | X |
| Total RNA | X |  |  |  |  |  |  |  | X |
| *In Vitro* Culture | X |  |  |  |  |  |  |  | X |
| Antimalarial Drug treatment | X | X | X | X | X | X | X |  | X |

* Daily for 7 days, or until parasite clearance, whichever is longer; and on Days 14, 21, 28, 35 and 42

** On Day 0 at 0, 2, 4, 6, 8, 12, 18 and 24 hours, then daily (up to 4 times per day) until aparasitemic on at least 2 successive smears (blood sample is from blood draw except finger prick is at 12 and 18 hours)..

*** Glucose only Day 0 or when clinically warranted

^ Drug levels on Day 0 (just before and 15, 30, 60 (±10 minutes), 2, 4, 6 and 8 hours (±30 minutes) after drug intake).

^^ Drug levels on Day 6 (just before and 2, 4 and 6 hours (±30 minutes) after drug intake).

# PCR sample 8 ml on admission and day of failure; all other samples 3-4 drops blood taken with malaria blood smear

“ CBC and ALT to be collected on Day 14 only

**APPENDIX B: Roles and Responsibilities**

Dr. Mark Fukuda and Dr. Duong Socheat and Dr. Youry Se: Responsible for all aspects of study to include: Protocol and consent form design. Supervision and monitoring of field research staff, protocol compliance and QA/QC plan execution, and completion of AE documentation, clinical aspects of the study; perform all duties in accordance with GCP Guidelines; Oversees execution and supervision of all clinical work. Ensures timely and accurate reporting of AEs and SAEs to IRBs and AFRIMS management.

Dr. Chanthap Lon : Responsible for all aspects of study to include: Protocol and consent form design. Supervision and monitoring of field research staff, protocol compliance and QA/QC plan execution, and completion of AE documentation, clinical aspects of the study; perform all duties in accordance with GCP Guidelines; report AEs and SAE to IRBs.

Dr Sok Peou, Dr Sea Darapiseth and: Protocol and consent form design. Responsible for multiple aspects of the study to include: supervision and monitoring of research staff, protocol compliance and QA/QC plan execution, and completion of AE documentation, clinical and scientific aspects of the study and annual and final study reports and publication. Report SAEs to IRBs, RCQ and USAMMDA. Perform all duties in accordance with GCP Guidelines. Liaison with local investigators.

Dr. Delia Bethell, Dr. Bryan Smith and Dr David Saunders; Protocol and consent form design, execution and supervision of clinical work, scientific aspects of the study and final study reports and publication. Report AEs and SAEs to the PI; monitoring of staff, protocol compliance and will perform all duties in accordance with GCP Guidelines.

Mr. Ses Sarim: Responsible for execution and supervision of all clinical work.

Dr. Kurt Schaecher, Dr. Paktiya Teja-Isavadharm and Wiriya Rutvisuttinunt: Protocol design, supervision and monitoring of pharmacology, molecular and *in vitro* testing of study specimens. Perform all duties in accordance with guidelines.

Clinical Research Coordinator (CRC): Ms Sabaithip Sriwichai. Responsible for attending team meetings regarding research protocol; responsible for ensuring enrolled subjects meet eligibility criteria; ensuring informed consent process; responsible for protocol compliance, that all AEs are documented and reported, that out-patient follow-up appointments are kept, reporting data trends to the PI, establishing practices to ensure the quality of the data, and ensuring conduct of trial follows GCP Guidelines.

Laboratory Coordinator: Mr. Sittidech Surasri and Mr. Montri Arsanok. Responsible for attending team meetings regarding research laboratory procedures; responsible for ensuring any procedure compliance with protocol, SOPs, and SSPs.

Dr. Koy Lenin, Medical Monitor. Responsible for monitoring the study subjects from a medical perspective, reviewing and reporting all serious and unexpected adverse events, ensuring medical care for any such events should they occur, and reporting serious events to higher headquarters. He will perform on-site visits approximately every 3 months and as needed.

Mrs. Mali Ittiverakul, Monitor. Responsible for regular monitoring to ensure the human subject protection, study procedures, laboratory, and data collection processes are of high quality and meet GCP/ICH and regulatory guidelines and contact IRBs.

**Appendix C:**

**Charter of the Safety Monitoring Committee** for the study**,** "*Artemisinin Resistance in Cambodia 2*", to be conducted by the Armed Forces Research Institute of Medical Sciences (AFRIMS), Bangkok, Thailand, WRAIR Study Number 1396, *HSRRB Log Number A-14479*.

Version 1.0

Date: 21 April 2008

Institutions: Armed Forces Research Institute of Medical Sciences (AFRIMS)

Dept. of Immunology and Medicine

315/6 Rajvithi Road, Bangkok 10400, Thailand

Tel. 66-2-644-5775; Fax 66-2-644-4784

National Center for Parasitology, Entomology and Malaria Control

#372, Monivong Blvd., Phnom Penh, Cambodia

Tel.: +855 23 211 926; Fax.: +855 23 996 202

Principal Investigators: LTC Mark M. Fukuda, M.D.

Dept. of Immunology and Medicine

AFRIMS, Bangkok

Tel. 66-2-644-5775, Fax 66-2-644-4784

Email: Mark.Fukuda@afrims.org

*Duong Socheat, M.D.*

*National Center for Parasitology, Entomology and Malaria Control*

*Office: #372, Monivong Blvd.*

*Phnom Penh, Cambodia*

*P.O. box 1062*

*Tel.: 855 23 211 926, Fax: 855 23 211 926*

*Email: socheatd@cnm.gov.kh*

*Youry Se, M.D., M.P.H.M.*

*Armed Forces Research Institute of Medical Sciences (AFRIMS)*

*No. 2. Kim Yl Sung Boulevard*

*Khan Tuol Kork, Phnom Penh, Cambodia*

*Tel: 855 (0) 12 992-029*

*Email: youry@online.com.kh*

SMC Chairperson: To be appointed

Tel. Fax

Email:

**Abbreviations used in this Charter**

AE Adverse event

AFRIMS Armed Forces Research Institute of Medical Sciences

FCT Fever clearance time

GCT Gametocyte clearance time

IRB Institutional Review Board

PCT Parasite clearance time

PI Principal Investigator

SAE Serious adverse event

SMC Safety Monitoring Committee

**Table of contents**

[**1. Role of the SMC**](../ARC2%20ver%201%205%2023%20April.doc) [75](#__RefHeading___Toc83551831)

[**1.1 General**](../ARC2%20ver%201%205%2023%20April.doc) [**75**](#__RefHeading___Toc83551832)

[**1.2 Specific**](../ARC2%20ver%201%205%2023%20April.doc) [**75**](#__RefHeading___Toc83551833)

[**2. Responsibilities**](../ARC2%20ver%201%205%2023%20April.doc) [77](#__RefHeading___Toc83551834)

[**2.1 Principal Investigator**](../ARC2%20ver%201%205%2023%20April.doc) [**77**](#__RefHeading___Toc83551835)

[**2.2 Medical Monitor**](../ARC2%20ver%201%205%2023%20April.doc) [**78**](#__RefHeading___Toc83551836)

[**3. Composition of SMC**](../ARC2%20ver%201%205%2023%20April.doc) [78](#__RefHeading___Toc83551839)

[**4. Procedures for SMC Meetings**](../ARC2%20ver%201%205%2023%20April.doc) [79](#__RefHeading___Toc83551840)

[**4.1 Meeting quorum**](../ARC2%20ver%201%205%2023%20April.doc) [**79**](#__RefHeading___Toc83551841)

[**4.2 Meeting format**](../ARC2%20ver%201%205%2023%20April.doc) [**79**](#__RefHeading___Toc83551842)

[**4.3 Schedule of review of data**](../ARC2%20ver%201%205%2023%20April.doc) [**79**](#__RefHeading___Toc83551843)

[**4.4 Meeting minutes**](../ARC2%20ver%201%205%2023%20April.doc) [**80**](#__RefHeading___Toc83551844)

[**5. Reporting of adverse events per protocol**](../ARC2%20ver%201%205%2023%20April.doc) [80](#__RefHeading___Toc83551845)

[**5.1 AE**](../ARC2%20ver%201%205%2023%20April.doc)

[**6. Procedure for reporting SMC recommendations to the Sponsor**](../ARC2%20ver%201%205%2023%20April.doc) [80](#__RefHeading___Toc83551847)

[**7. Confidentiality**](../ARC2%20ver%201%205%2023%20April.doc) [80](#__RefHeading___Toc83551848)

[**8. Conflict of interest**](../ARC2%20ver%201%205%2023%20April.doc) [80](#__RefHeading___Toc83551849)

**9. Signature Page………………………………………………………………………**[**81**](../ARC2%20ver%201%205%2023%20April.doc)

[**Appendix 1:** 0](#__RefHeading___Toc83551850)

[**Outline of contents for Safety Reports for the SMC**](../ARC2%20ver%201%205%2023%20April.doc) [82](#__RefHeading___Toc83551851)

**1. Role of the SMC**

**1.1 General**

The SMC will provide independent advice on safe and ethical conduct of the trial. The SMC will provide recommendations about stopping, continuing or modifying the trial based on review of the safety data during the study, bearing in mind its overall responsibility to protect the ethical and safety interest of the subjects recruited into this study.

The SMC should remain respectful of the laws and practices governing research and medical practice in Cambodia. In addition, SMC should be aware of the approval status of the IRBs approving the study (The U.S. Army Human Use Research Committee of the Walter Reed Army Institute of Research, the Human Subjects Research Review Board of the USAMRMC, the National Committee for Health Research- Cambodia, and the Research Ethics Review Committee of the World Health Organization).

Internationally acceptable ethical principles and guidelines as described in ICH GCP should guide the SMC.

The SMC functions as an independent board and should be free of undue influences and conflicts of interest in providing advice and making recommendations.

**1.2 Specific**

Once it develops and spreads, resistance to artemisinin derivatives, currently the most essential antimalarial drugs for the treatment of *Plasmodium falciparum* malaria, could very well be the most devastating event in the history of malaria control in the 21^st^ century. There is an urgent need for early detection and to investigate recent reports of treatment failures with advanced artemisinin combination therapies as well as artesunate monotherapy along Cambodia’s western borders.

Recent data indicate reduced overall sensitivity of *P. falciparum* to artemisinin derivatives both *in vivo* as well as *in vitro* along the Cambodian-Thai border. In our recent study, “Artemisinin Resistance in Cambodia” (WRAIR #1296, HSRRB A-13922) conducted at the same study site in 2006/7, individual *P. falciparum* isolates were detected that were highly suggestive of resistance to artemisinins. The study, which compared an experimental regimen of 4 mg/Kg of oral artesunate for 7 days (28 mg/Kg total dose) versus a standard comparator regimen of oral quinine and tetracycline for 7 days, found 4/60 patients in the artesunate arm who had re-emergence of *P. falciparum* parasites during 28 days of follow-up This study also found that a worrying 22% of subjects in the artesunate arm were still parasitemic after 72 hours, and indeed, the median parasite clearance time (PCT) in the 4 patients who later recrudesced was 97.6 hours compared to 52.2 hours in the patients who were cured. Malaria parasites collected from the two subjects who were ultimately found to meet all of the *a priori* criteria for artemisinin resistance were also found to have a reduced susceptibility to DHA *in vitro* with IC_50_s of 14.0 and 14.4 nM as compared to a mean value of 3.34 nM for from patients who were cured. This project is a follow-up study to the ARC1 study. The aim of this project is to better determine whether regimens with increased artesunate doses can overcome the problem of reduced drug sensitivity to artemisinins and to determine whether these experimental regimens are safe and well tolerated.

The primary objectives of the study are:

- To determine the impact of varying doses of artesunate on treatment outcome and whether higher doses of artesunate can overcome the problem of compromised artemisinin sensitivity in the region.
- To determine the safety and tolerability of this previously untested experimental high dose (6 mg/Kg/day x 7 days, total 42 mg/Kg) artesunate monotherapy regimen.

The secondary objectives of the study are:

- To validate treatment response parameters (PCT, FCT, GCT and the proportion of patients still parasitemic on Day 3) for their role in predicting failures.
- To further evaluate the current malaria *in vitro* drug susceptibility in this area.
- To validate potential genetic markers of artemisinin resistance and the role of identification of low level parasitemia by PCR

The specific responsibilities of the SMC include:

1. Review charter; make any recommendations for changes; agree and sign.

2. Review the study protocol with special attention to safety monitoring procedures and make recommendations for changes as necessary. No subjects may be enrolled until the IRB approved protocol is also approved by the SMC chairperson.

3. The SMC chairperson will review any study intervention-related events as determined by the Principal Investigator and Medical Monitor, serious adverse events (SAEs) as needed and confer with other members as necessary.

4. The SMC will review all SAEs and systemic grade 3 adverse events (AEs), whether related or not to study interventions.

5. Protocol defined stopping or holding rules should be reviewed and agreed upon by the SMC chairperson.

6. Minutes of SMC meetings should describe the outcome and recommendations made by the committee and will be used to communicate to the study team and be available as a report to Ethical Review Boards/Committees

**2. Responsibilities**

**2.1 Principal Investigator**

The Principal Investigator must report any serious adverse event which occurs at any time during the trial to the medical monitor, the local Ethics, U.S. Army and World Health Organization ethical review committees, and to the SMC Chairman within 24 hours, using electronic, fax, and telephone or other appropriate means.

The Principal Investigator will make every effort to explain each adverse event and assess its causal relationship, if any, to the study drugs.

A written report will be prepared by the Principal Investigator that will detail:

- Report date of adverse event
- Volunteer’s code
- Subject’s date of birth and gender
- Investigator’s name
- Cohort of participation
- Dates of study-related activities
- Date of onset
- Full description of the event and the consequences for the volunteer’s health and his/her ongoing participation in the trial
- Signs/symptoms and severity
- Action taken, concomitant medication, including dose, route and duration of treatment and date of last dose
- Date of resolution or death
- Assessment of relatedness to study activities and of expectedness
- Specific recommendations for changes to the protocol and informed consent; and what steps, if any, need to be taken to assure the safety of other volunteers.
- The PI will inform the SMC of any amendment to the protocol, informed consent or any other revisions to relevant trial documents
- The PI will inform the SMC in writing when:
  - The study starts
  - The first 5 subjects in the high-dose cohort has been enrolled and completed their first 14 days of follow-up
  - The enrolment is complete
  - The study is analyzed
  - The study is complete
  - Any potential safety concerns that may arise in the course of the trial that were previously unreported

**2.2 Local Clinical Safety/Medical Monitor**

Department of Defense regulation 3216.2 mandates and describes the role of a medical monitor for all GTMR research studies involving human subjects as follows:

- For research involving more than minimal risk (as defined in 32 CFR 219.102(i), reference (c)) to subjects, an independent medical monitor shall be appointed by name. Medical monitors shall be physicians, dentists, psychologists, nurses, or other healthcare providers capable of overseeing the progress of research protocols, especially issues of individual subject/patient management and safety. Medical monitors shall be independent of the investigative team and shall possess sufficient educational and professional experience to serve as the subject/patient advocate.

- Depending on the nature of the study, the medical monitor may be assigned to assess one or more of the following phases of a research project: subject recruitment, subject enrollment, data collection, or data storage and analysis.

- At the discretion of the IRB, the medical monitor may be assigned to discuss research progress with the principal investigator, interview subjects, consult on individual cases, or evaluate adverse event reports. Medical monitors shall promptly report discrepancies or problems to the IRB. They shall have the authority to stop a research study in progress, remove individual subjects from a study, and take whatever steps are necessary to protect the safety and well-being of research subjects until the IRB can assess the medical monitor's report.

**2.3 Clinical trials Monitor**

A clinical trial Monitor will be assigned to conduct the monitoring and evaluation of the conduct of the trial to ensure adherence to ICH GCP guidelines. This will include pretrial, interim and trial close-out monitoring and evaluation visits to the trial site. The Clinical trials monitor will be appointed as per the protocol to conduct the monitoring and evaluation of the study.

**3. Composition of SMC**

SMC members will consist of the Chairman, a second AFRIMS physician familiar with malaria treatment and expected clinical course, and a third non-AFRIMS affiliated member with familiarity with malaria treatment, the artemisinins and drug development.

**4. Procedures for SMC Meetings**

**4.1 Meeting quorum**

A minimum or quorum of at least 2 out of three members is required at meetings or teleconferences. Decisions and recommendations of the SMC must have the consensus of all members. If the SMC chairperson is unable to attend a meeting or teleconference he may designate another member of the SMC to act as chairperson at that meeting.

The SMC may request the presence of an ad-hoc member, to act as a consultant or expert on a specific safety concern, should this arise in the course of the trial.

**4.2 Meeting format**

The SMC will receive, at a minimum all SAE reports and a quarterly grade 3 AE summary report for review. The meetings will consist of open and closed portions. During the open portion of the meeting, the Principal Investigator may make a brief presentation and be available for questions as requested by the SMC.

Only the SMC members should attend the closed portion of the meeting, unless the SMC decides otherwise.

**4.3 Schedule of review of safety data**

All SAEs, Grade 2 or 3 AEs, and clinical safety labs for the high-dose cohort will be reviewed by the SMC after the first 5 subjects are enrolled and have completed the first 14 days of treatment follow-up.

Following this initial rescheduled review, the SMC will be informed of any subject meeting the protocol defined Individual Halting Rules, and any SAEs or Grade 3 AEs. A full meeting of the SMC may be called at the Chairman’s discretion at any time. Any cohort, which is halted secondary to the protocol defined Cohort Halting Rules will similarly be referred for full SMC review.

The SMC may request any additional data to supplement the scheduled and unscheduled reports.

**4.4 Meeting minutes**

Summary minutes of the SMC meetings will be prepared and distributed in a timely manner for sign-off by the members. The minutes should reflect any decisions, rationale for recommendations and other relevant deliberations as appropriate.

**5. Reporting of adverse events per protocol**

Protocol Section 9 describes the definitions and reporting of adverse events. Any routine reports made to the IRBs should also be copied to the SMC Chairperson

**6. Procedure for reporting SMC recommendations**

The SMC chairperson will communicate the committee's decisions and recommendations with accompanying SMC meeting minutes in a timely manner. SMC recommendations will be communicated in writing to the PI who will forward it to the Medical Monitor and the IRBs.

**7. Confidentiality**

No confidentiality agreement will be required.

**8. Conflict of interest**

The SMC membership should be restricted to individuals free of apparent significant conflict of interest with the investigational team. The source of this conflict may be scientific, financial or regulatory in nature. Neither the study investigators nor individuals directly employed within the same Department should be members of the SMC. Membership should be for the duration of the project including time for generation of post-study final report.

Signature Page

I hereby endorse and agree with the terms of this charter for the Safety Monitoring Committee and its proposed oversight role as described herein.

__________________________ ________________

Dr Youry Se Date

Principal Investigator

__________________________ ________________

Dr. Mark M. Fukuda Date

Principal Investigator

__________________________ ________________

Dr. Duong Socheat Date

Principal Investigator

# Appendix 1: Outline of contents for Safety Reports for the SMC ( to be prepared by the Principal Investigator and Medical Monitor)

The Safety report to be submitted to the SMC should contain the following information in a tabular format.

1. Summary of all Adverse Events

Template

| Adverse Event | Total | Grade | | | |
| --- | --- | --- | --- | --- | --- |
|  |  | 0 | 1 | 2 | 3 |
|  |  |  |  |  |  |

2. Summary of laboratory investigation

3. Grade 3 adverse events summary.

Template

| ID# | Gender | Date | Grade 3 AE | Start date | Resolution date | Causality | Action taken | Comments |
| --- | --- | --- | --- | --- | --- | --- | --- | --- |
|  |  |  |  |  |  |  |  |  |
|  |  |  |  |  |  |  |  |  |

4. Summary of SAEs

Template

| ID# | Gender | Date | SAE  (grade) | Start date | Resolution date | Causality | Action taken | Comments |
| --- | --- | --- | --- | --- | --- | --- | --- | --- |
|  |  |  |  |  |  |  |  |  |
|  |  |  |  |  |  |  |  |  |
